# Absence of Ancient DNA in Sub-Fossil Insect Inclusions Preserved in ‘Anthropocene’ Colombian Copal

**DOI:** 10.1371/journal.pone.0073150

**Published:** 2013-09-11

**Authors:** David Penney, Caroline Wadsworth, Graeme Fox, Sandra L. Kennedy, Richard F. Preziosi, Terence A. Brown

**Affiliations:** 1 Faculty of Life Sciences, University of Manchester, Manchester, United Kingdom; 2 Manchester Institute of Biotechnology, University of Manchester, Manchester, United Kingdom; Natural History Museum of Denmark, University of Copenhagen, Denmark

## Abstract

Insects preserved in copal, the sub-fossilized resin precursor of amber, have potential value in molecular ecological studies of recently-extinct species and of extant species that have never been collected as living specimens. The objective of the work reported in this paper was therefore to determine if ancient DNA is present in insects preserved in copal. We prepared DNA libraries from two stingless bees (Apidae: Meliponini: *Trigonisca ameliae*) preserved in ‘Anthropocene’ Colombian copal, dated to ‘post-Bomb’ and 10,612±62 cal yr BP, respectively, and obtained sequence reads using the GS Junior 454 System. Read numbers were low, but were significantly higher for DNA extracts prepared from crushed insects compared with extracts obtained by a non-destructive method. The younger specimen yielded sequence reads up to 535 nucleotides in length, but searches of these sequences against the nucleotide database revealed very few significant matches. None of these hits was to stingless bees though one read of 97 nucleotides aligned with two non-contiguous segments of the mitochondrial cytochrome oxidase subunit I gene of the East Asia bumblebee *Bombus hypocrita*. The most significant hit was for 452 nucleotides of a 470-nucleotide read that aligned with part of the genome of the root-nodulating bacterium *Bradyrhizobium japonicum*. The other significant hits were to proteobacteria and an actinomycete. Searches directed specifically at Apidae nucleotide sequences only gave short and insignificant alignments. All of the reads from the older specimen appeared to be artefacts. We were therefore unable to obtain any convincing evidence for the preservation of ancient DNA in either of the two copal inclusions that we studied, and conclude that DNA is not preserved in this type of material. Our results raise further doubts about claims of DNA extraction from fossil insects in amber, many millions of years older than copal.

## Introduction

Museum entomology collections represent an unparalleled wealth of species biodiversity and distribution data for all insect groups from around the globe. This information is becoming increasingly accessible as more museums host searchable catalogues online. Such archived samples are irreplaceable, hence destructive sampling for attempted DNA extraction is usually discouraged by museum curators. Although there has been only limited success in obtaining DNA sequences from samples such as detached legs from air-dried insects [1], there has been promising progress in the development of non-destructive methods of DNA recovery from museum insects, including specimens from as far back as 1820 that have been pinned and dried in the traditional manner [2]–[6]. These developments have numerous and diverse implications, particularly as many entomology collections hold large series of individual species allowing for replicated data to be obtained for extended time periods. For extinct species, museum specimens represent ‘fossils on a pin’ [7], and sequence retrieval from these would aid positioning of the taxa within phylogenies. This would be particularly useful when investigating extinct, endemic island forms, not just to study their evolutionary relationships but also to elucidate their historical biogeographies in order to understand the cause of their insular isolation. An example would be the so-called dodo of the insect world, the St Helena giant earwig *Labidura herculeana*, which appears to have become extinct during the mid-twentieth century as a result of habitat destruction and the introduction of non-indigenous predators by humans [8]. Alternatively, fauna once common on islands may now be locally extinct but still exist in continental populations, such as the large tortoiseshell butterfly *Nymphalis polychloros* now considered extinct in Britain but present in Europe [9].

Another consideration is the difficulty in collecting insects in certain regions, due to funding constraints, armed conflict, local wildlife conservation laws, etc. It is not unheard of for entomologists to receive prison sentences for collecting insects without the appropriate permits [7], [10]. Some species may be so rare that there is no guarantee of collecting them during fieldwork. Another potential benefit of DNA data from museum insects would be generation of a baseline dataset for examining the molecular effects of past pollution events, such as atomic bomb tests and other forms of radiation or chemical exposure, as recently demonstrated for butterflies following the Fukushima nuclear accident [11].

Similar information might be obtained from sub-fossil insects in copal, the sub-fossilized resin precursor of amber, renowned for preserving insects with life-like fidelity [12], [13]. Most palaeontologists consider copal to be too young to be of interest because many of the inclusions belong to extant species [14], [15], but this material has potential value at many levels, including molecular palaeobiology [16], [17]. Some of the species found in copal are thought to be extant but have not been collected as living specimens, and at least some of the taxa that have been formally described are considered extinct (e.g. the orchid bee described from Colombian copal [18]). Sub-fossil copal inclusions therefore represent the only source of genetic information for these extinct and extant but elusive species.

The objective of the work reported in this paper was to determine if ancient DNA (aDNA) is present in insects preserved in copal. Intuitively, one might imagine that the complete and rapid engulfment in resin, resulting in almost instantaneous demise, might promote the preservation of DNA in a resin entombed insect. Within this protective environment, DNA preservation might be better than in an air-dried museum specimen. This rationale has been used to explain the remarkable preservation of DNA in million-year-old insects present in amber inclusions [19]–[24]. However, these amber DNA sequences have been questioned [25], with doubts regarding their authenticity arising because the results were obtained after DNA amplification by the polymerase chain reaction (PCR), which will preferentially amplify any modern, undamaged DNA molecules that contaminate an extract of partially degraded ancient ones [26]. This approach, which has also been used with insects from museum collections [2]–[6] and archaeological deposits [27], can therefore give false positive results that might be mistaken for genuine aDNA sequences if careful authentication procedures are not followed. In other areas of aDNA research, many of the limitations of the conventional PCR approach to sequence retrieval have been sidestepped by switching to ‘next generation’ sequencing methods [28]. These methods are ideal for aDNA because they provide sequences for all the DNA molecules in an extract, regardless of their length, and are less likely to give preference to contaminating modern molecules. We therefore applied next generation sequencing to extracts prepared from two stingless bee inclusions from Colombian copal, dated to ‘post-Bomb’ and 10,612±62 cal yr BP, respectively.

## Materials and Methods

All manipulations up to and including preparation of sequencing libraries were carried out within the aDNA facility at the University of Manchester, which comprises a suite of independent, physically isolated laboratories, each with an ultrafiltered air supply maintaining positive displacement pressure and a managed access system. All surfaces within the laboratories were periodically sterilised by UV irradiation and cleaned with 30% bleach and 70% ethanol, and all utensils and equipment were treated with DNA-Away (Molecular Bioproducts) before and after use. Items such as test tubes were UV irradiated (254 nm, 120,000 µJ cm^-2^ for ×5 min, with 180° rotation between the two exposures) before use. Aqueous solutions we similarly irradiated for 15 min. Personnel wore protective clothing including forensic suits, face masks, hair nets, goggles and two pairs of sterile gloves at all times. Specimen preparation and DNA extraction were carried out in a Class II biological safety cabinet in one laboratory within the facility, and library preparation in a second, physically-isolated laboratory. Modern insect DNA had never been handled in the building within which these laboratories are located.

The specimens were two stingless bees (Apidae: Meliponini: *Trigonisca ameliae* [29]) in ‘Anthropocene’ Colombian copal (taken from the research collection of DP, University of Manchester), dated to ‘post-Bomb’ (<60 years in age) and 10,612±62 cal yr BP, respectively (NSF–Arizona AMS Facility). Due to the destructive nature of the research nothing remained of either specimen, so no repository data are available, although voucher specimens (holotype and paratypes) of the species used are held in the Palaeontology Department of the Natural History Museum, London under the repository number: NHM II 3059 [29]. Each specimen was trimmed to a small workable size using a saw (Hi-Tech Diamond) and then further shaved down using a scalpel into a small cube c.4 mm^3^ and 0.4 g, taking care not to expose the inclusion. The surface of the cube was cleaned with 30% bleach, followed by absolute alcohol and DNA-Away. The remaining copal was removed by dissolution in chloroform [29]. The sample was first placed in 5 ml chloroform for 15 min to dissolve off the surface layer of the copal, and then transferred to another 5 ml chloroform and incubated at 40^°^C for 48 h to 8 days to dissolve the remaining ‘resin’.

Each insect was removed from the dissolved copal and rinsed in 2 ml absolute alcohol followed by 2 ml UV-irradiated HPLC grade water. DNA extraction was immediately carried out. For non-destructive DNA extraction, the insect was incubated in 1 ml extraction buffer (100 mM Tris-HCl pH 8.0, 100 mM NaCl, 3 mM CaCl_2_, 40 mM dithiothreitol, 2% sodium dodecyl sulphate, 250 µg ml^–1^ proteinase K) for 20 h at 55°C with gentle agitation [4]. Each insect was then transferred to 1 ml fresh extraction buffer and crushed until no large pieces remained. These destructed samples were then incubated in the extraction buffer for 20 h at 55°C with gentle agitation. DNA was then isolated from both the non-destructive and destructive extracts using a QiaQuick PCR purification kit (Qiagen).

Sequencing was carried out with the GS Junior 454 System (Roche). The non-destructive and destructive extracts for each specimen were barcoded and pooled and two libraries prepared, one for each specimen. The GS FLX Rapid Library MID Adaptors Kit was used in accordance with the manufacturer's instructions, except that the DNA fragmentation step was omitted, due to the anticipated short lengths of any aDNA molecules in the extracts. The library was eluted in 30 µl TE buffer (10 mm Tris-HCl pH 8.0, 1 mm EDTA) and this entire amount used for emulsion amplification with the Titanium emPCR kit, again following the manufacturer's instructions, including the specific recommendations for short-length amplicon libraries. As enrichment was very low, three emulsions were prepared for each library and these combined before loading on to the sequencing chip. Sequencing was carried out with the Titanium sequencing kit using the preset filters, except for changing the vfTrimBackMinimumLength parameter from 84 flows to 20 flows so that short reads would not be excluded. All the test reads passed the standard quality controls and control reads were of good quality. As the test read numbers were very low, duplicates were not removed prior to downstream processing. Sequence reads were converted into FASTA format and searched using MEGAN4 [30] and BLAST [31] against the NCBI nucleotide database and against a database of NCBI entries described as Apidae (dated May 2012). For MEGAN4, standard LCA parameters were used (Min support 5, Min Score 50, Top percentage 10, Win score 0, Min complexity 0.44) and for BLAST the standard blastn parameters were used (word size 11, DUST filter on, expect value 10, match/mismatch score 2,–3, gap costs existence 5 extension 2).

## Results

As we have previously reported [29], incubation with chloroform using the described procedure results in dissolution of the copal matrix, leaving the ‘resin’ as a viscous fraction floating on the surface of the solvent. Each intact insect was released from its copal matrix and positioned immediately below this fraction, visibly resembling a recent entomological specimen preserved in alcohol.

Sequencing results are summarised in Table 1. Read numbers were low, and could not be increased by adjusting the filter parameters. Significantly more reads were obtained after destruction of the specimen compared with extracts prepared by the non-destructive method. For specimen 1, the younger of the two, sequences obtained by the destructive method were also longer on average (destructive, 100.70 nucleotides; non-destructive, 49.33 nucleotides). MEGAN4 searches of these sequences against the complete nucleotide database revealed no significant matches, and BLAST searches gave only very few hits. Setting the maximum Expect (E) value at 10^–6^, two hits were obtained for the sequences from the non-destructive extract, and eight for the destructive library (Table 2). None of these hits was to stingless bees though one read of 76 nucleotides aligned with two non-contiguous segments of the mitochondrial cytochrome oxidase subunit I gene of the East Asia bumblebee *Bombus hypocrita*. The most significant hit, with an E value of 5×10^–98^ was for 452 nucleotides of a 459-nucleotide read that aligned with part of the genome of the root-nodulating bacterium *Bradyrhizobium japonicum*. The other hits with E values <10^–6^ were to the proteobacteria taxa *Burkholderia*, *Xanthomonas*, *Mesorhizobium*, *Thiobacillus*, *Ralstonia* and *Stenotrophomonas*, and the actinomycete *Gordonia polyisoprenivorans*. BLAST searches of the Apidae nucleotide database gave four additional hits with E values <10^–6^, but the longest of these alignments was only 29 bp and none involved the full length of a sequence read.

**Table 1:** Summary of DNA sequencing results.

Specimen	Age	Preparation method	Number of reads^a^	Number of unique reads	Length of sequences (nucleotides)
1	<60 years	Non-destructive	30	30	25–100 (ave. 49.33)
		Destructive	460	460	24–536 (ave. 100.70)
2	10,612±62 cal yr BP	Non-destructive	54	28	34–44 (ave. 39.44)
		Destructive	1335	103	33–47 (ave. 40.36)

a The anticipated performance of the GS Junior 454 System with control modern DNA is c.100,000 reads per run.

**Table 2:** Sequence reads from specimen 1 giving BLAST hits with an E value ≤10^–6^.

Library/database	Read length (nucleotides)	Read ID	E value	Matches	Database entry
Non-destructive extraction, complete database	76	HRA5KJN01BI264	8×10^–19^	51/51 and 31/31^a^	*Bombus hypocrita* mitochondrial cytochrome oxidase subunit I gene
	43	HRA5KJN01ANAS1	2×10^–6^	33/34	*Burkholderia* sp. genome
Non-destructive extraction, Apidae database	76	HRA5KJN01BI264	8×10^–19^	51/51 and 31/31^a^	*Bombus hypocrita* mitochondrial cytochrome oxidase subunit I gene
Destructive extraction, complete database	459	HRA5KJN01BDMRJ	5×10^–98^	385/452	*Bradyrhizobium japonicum* genome
	98	HRA5KJN01BP4B0	3×10^–16^	59/63	*Xanthomonas oryzae*, *X. campestris* genomes
	53	HRA5KJN01A8GD4	2×10^–15^	45/45	*Gordonia polyisoprenivorans* genome
	178	HRA5KJN01AXXA2	10^–13^	49/51^b^	*Mesorhizobium loti* genome
	178	HRA5KJN01AXXA2	10^–13^	55/59^b^	*Thiobacillus denitrificans* genome
	157	HRA5KJN01B26IX	6×10^–12^	55/60 or 58/64^c^	*Ralstonia solanacearum* genome
	53	HRA5KJN01A73N7	2×10^–9^	35/35	*Burkholderia* sp. genome
	38	HRA5KJN01BZ4HK	9×10^–8^	32/32	*Stenotrophomonas maltophilia* genome
Destructive extraction, Apidae database	45	HRA5KJN01A1IJM	2×10^–07^	18/18	*Bombus terrestris* mRNA sequence
	516	HRA5KJN01BVPD4	9×10^–06^	17/17	*Ceratina propinqua* elongation factor 1 alpha gene
	40	HRA5KJN01BPOO5	8×10^–06^	16/16	*Apis mellifera* proteasome activator complex subunit gene
	46	HRA5KJN01ASAM2	1×10^–06^	28/29	*Apis mellifera* genome

See Table S1 for sequence reads.

a Two overlapping segments of this read gave hits to non-contiguous regions of the *Bombus hypocrita* gene.

b The same sequence gave hits with identical E values to both *Mesorhizobium loti* and *Thiobacillus denitrificans*.

c The alignment is slightly different for different strains of *Ralstonia solanacearum.*

Specimen 2, the older of two inclusions, yielded 54 and 1335 sequences from the non-destructive and destructive extracts, respectively, but both of these sets comprised multiple copies of similar sequences, and we assume that they resulted from artefacts generated during library construction.

## Discussion

We were unable to obtain any convincing evidence for the preservation of endogenous DNA in either of the two copal inclusions that we studied. All the reads obtained from the two libraries prepared from the older specimen, dated to 10,612±62 cal yr BP, appeared to be artefacts. The younger specimen, which was ‘post-Bomb’ (<60 years in age), gave sequences, but other than one read which partially aligned with a bumble bee mitochondrial DNA sequence, none of these had convincing matches with taxa related to stingless bees.

We do not believe that our negative results, from two sequencing libraries prepared from four extracts, can be ascribed entirely to technical incompetence. In previous and current projects we have successfully prepared aDNA libraries for next generation sequencing from *Mycobacterium tuberculosis* in human bone [32] and archaeological plant remains [33], and one of us (GF) routinely prepares and sequences libraries for the Junior 454 System from modern samples on a contract basis. The two copal specimens were extracted and sequenced at different times, with approximately six months elapsing between the two experiments. During this period the same personnel used an identical workflow for three separate sequencing runs with DNA prepared from two archaeological bones, obtaining 36,570, 32,271 and 95,655 reads, respectively, the last of these close to the sequencing system's anticipated performance of 100,000 reads for shotgun sequencing of modern DNA. Neither do we believe that the specimen preparation and DNA extraction methods could have resulted in a complete loss of an endogenous DNA fraction that was originally present in the specimen while it was contained in its copal matrix. Release of the insect from the copal simply involved incubating in chloroform, which would not be expected to degrade DNA, and once released the insects were subjected to a standard procedure for DNA extraction that has been shown to be applicable to air-dried specimens [4], [5]. We therefore conclude that our failure to obtain sequence reads was because the copal specimens contained no preserved DNA.

With the older specimen the absence of aDNA is incontrovertible because the only reads that we obtained were artefacts. For the younger inclusion it might be possible to argue that the reads derived from highly degraded aDNA from the insect and its microflora at the time of death. Although the vast majority of the reads gave very low significance scores in BLAST searches against the complete nucleotide database as well as a database made up of Apidae sequences, a case could be made for some of these being insect or microfloral in origin. As well as the few reads that gave BLAST matches with a E value <10^–6^, which aligned with sequences from bumble bee (one read) and common environmental bacterial taxa (nine reads), others gave lower significance scores that might be interpreted as poor quality aDNA sequences. For example, one 61-nucleotide read obtained from the non-destructive library could be interpreted as 23 nucleotides of bee DNA (hit to *Apis florea*) followed by 21 nucleotides of insect gut bacterium (hit to *Sebaldella termitidis*). Similar chimeric interpretations could be made for other reads, as many of these contained at least one short segment that aligned with an Apidae sequence. However, in most cases, the Apidae alignment was less than 20 bp, and few were longer than 30 bp, which is hardly the basis for a convincing argument bearing in mind the overall lengths of the sequence reads.

Our conclusion that DNA is not preserved in insect inclusions in copal contrasts with the reports of successful DNA amplification from dried and pinned museum specimens [2]–[6]. Although one of these papers [4] described failure to retrieve any DNA from their oldest specimen, dated at 94 years, others [3], [5] reported success from museum specimens collected as far back 1820, substantially older than our younger specimen. These previous studies focussed on arthropods and beetles rather than bees, and it is unclear if the insects had been preserved in liquids such as ethyl alcohol or formalin (as is common for entomological samples collected in the field) prior to being pinned and dried. Whether these taxonomic and preservation factors have a significant impact on long-term DNA integrity is unknown.

DNA degradation is influenced by factors such as oxygen and water content, ambient temperature and time since death of the organism [34]. We have a poor understanding of the physiochemical processes affecting insects preserved in resins and their subsequent diagenetic transformation into fossils in amber, and hence little information on which to base predictions about DNA degradation. Preservation in amber is often referred to as a type of mummification resulting from rapid fixation and dehydration of specimens trapped in the original resin secretion. It would seem likely that diagenetic events, including overburden pressure and heat generated through tectonic processes and orogenesis over millions of years, would minimise the likelihood of DNA preservation in amber specimens. However, the young age of the copal in our study means that it had not been subjected to such extreme processes. Certainly, the preservation and fossilization processes are not uniform for all copal and amber deposits, nor for all inclusions from a single deposit. For example, the recent application of X-ray computed tomography (CT) has shown that in some instances internal organs are preserved in amber specimens 50 million years in age [35], whereas digital dissection of a spider preserved in amber of a similar age revealed nothing substantial preserved internally [36]. Unfortunately, it is rarely possible to draw any conclusions about the degree of internal preservation using traditional light microscopy. Clearly, the better preserved a copal specimen is internally, then the more likely it is that DNA will survive. A recent study investigating the effects of CT scanning on aDNA recovery from c.100-year-old bird footpads has demonstrated that this technique has negligible impact on DNA integrity [37]. Hence, future studies may benefit from identifying specimens with preserved internal morphology prior to attempting aDNA extraction, assuming the X-ray energy levels required to scan the copal are maintained below a threshold at which DNA is damaged.

Theoretical and empirical data indicate that aDNA fragments might be present in well preserved geological material up to at least 100,000 years in age, and suggest that some material up to one million years might yield sequence data [38]. Copal inclusions fall at the lower end of this age range, but according to our results do not contain preserved DNA. This raises further doubts about claims of DNA extraction from fossil insects in amber, many millions of years older than copal. Amber is renowned for its remarkable preservation of insects and other inclusions [39], including in some cases at the subcellular level [40]. Reports of aDNA extraction from Tertiary and Cretaceous ambers [19]–[24] therefore held some attraction, but proved impossible to replicate independently [25], and are now regarded by many as an example of the problems caused by a failure to prevent contamination with modern DNA [38], a challenge that provided difficulties in all areas of aDNA research during the 1990s [26]. Counter-claims for the existence of aDNA in amber specimens focus on the possibility that the resin provides a protected environment for DNA preservation. Our inability to detect aDNA in copal specimens, despite using sensitive next generation methods, suggest that there is no protected environment in this type of material, and that DNA survival in resin inclusions is no better, and perhaps worse, than that in air-dried museum insects.

## Supporting Information

Table S1 **Sequence reads.**
(DOC)
