# Supplementary material for: Absence of Ancient DNA in Sub-Fossil Insect Inclusions Preserved in ‘Anthropocene’ Colombian Copal
Source: PLoS One. 2013 Sep 11;8(9):e73150. doi: 10.1371/journal.pone.0073150 (PMC3770633; doi:10.1371/journal.pone.0073150)
Supplement: Table S1 — Sequence reads. (DOC) [file pone.0073150.s001.doc]

**Absence of Ancient DNA in Sub-Fossil Insect Inclusions Preserved in ‘Anthropocene’ Colombian Copal**

**David Penney, Caroline Wadsworth, Graeme Fox, Sandra L. Kennedy, Richard F. Preziosi, Terence A. Brown**

**SUPPORTING INFORMATION**

**Table S1: Sequence reads**

**SPECIMEN 1 NON-DESTRUCTIVE PREPARATION**

>HRA5KJN01A0POS

ATATTGACAAATTCGAACAAGGCTTGATTCGAATTTGTCAATATA

>HRA5KJN01AB5AJ

GATCTTTCTGTCATAAACATTAAATTTGGGAATTGAGTATTTATGACAGAAAGATCA

>HRA5KJN01BI264

ATTGTAATTGCTCCTGCTAATACTGGTAATGATAAAATTAATAAAATTACCAGTATTAGCAGGAGCAATTACAATA

>HRA5KJN01AH0E6

GTAGATATGTCCTCATGTTTACCCTCTTCCCTTCATCCTACTAGTACATGGTGTATTGGTAGGATGAAGGGAAGAGGGTAAACATGAGGACATATCTACA

>HRA5KJN01BJRNP

GTCGGGACGCAACAGATAAACTCCGCTCGGACCAACAATTGACATGGCGAGGCCGTTGTCCGAGCGGAGTTTATCTGTTGCGTCCCGACA

>HRA5KJN01A5JZE

TATATATATATTTATATATATATATATTTATATATAGTATATATATTTATATATATATAA

>HRA5KJN01BNG8T

ATATATATATATAAATATATATATATA

>HRA5KJN01BIF0Y

GTAATATTTAGTTTGTTTCTGAACTATTTCAAAGTTCAGAAACAAACTAAATATTACA

>HRA5KJN01A4A97

GGCTTCTCTCTTATCGATGCATTAAACACCTTAATGCATCGATAAGAGAGAAGCCA

>HRA5KJN01A8DVA

ATTGTTAATGATTAATCGAAAATAAATATAGGACGTTATTTTCGATTAATCATTAACAATA

>HRA5KJN01AUN32

GTGCTCAGTGTCACCACGACACTGAGCACA

>HRA5KJN01A9IKF

ATACTGCACCTATATTTTTTAGCGAATGTTTGAAAATATAGGTGCAGTATA

>HRA5KJN01ALJIP

CTGCGTGTCTCCGACGACTACGTAGA

>HRA5KJN01ARE6B

ATTTTTTTCAAAATTATTTTTCAGAATTTTTTTCAAAAATAATTTTGAAAAAATA

>HRA5KJN01ANAS1

GTTCAATCCGCGCGAGTTCCGCGTGATCGCCGCGGATTGAACA

>HRA5KJN01ADEG5

CGATCTGCGTGTCGCGTCTCTCAGGCACACAG

>HRA5KJN01AIHML

CGATCTGCGTGGTCGGCGTTCAAGGCACACAGGGGATAGG

>HRA5KJN01ATL0U

ATATATATATATAATATATATATATA

>HRA5KJN01AKW3G

CGTACGTCGTGTCGTCGGTACGGGCGTCTACTACGGAAGGTACGACACGAGGGATAGG

>HRA5KJN01AADJG

AGTTCAATTGAAGTTAGTCACATTTTAAAATTATTTAAATGTGACTAACTTCAATTGAACTA

>HRA5KJN01BJ4M7

ATATATATATATACATATATATATA

>HRA5KJN01BBORH

ATAGTTATAAATCCTGAATTAAATGAATTTAATTCAGGATTTATAACTATA

>HRA5KJN01ARLDW

CTGCGTGTCTCCGACGACTACGTAGA

>HRA5KJN01AL46D

ATATTGACAAATTCGAACAAGGCTTGATTCGAATTTGTCAATATA

>HRA5KJN01A7D7Q

AGGTTCATGTACTTGCCAAAGTACATGAACCTA

>HRA5KJN01APK6A

ATATATATAAATATATATATATATATAATATATATATATATTTATATATATA

>HRA5KJN01AHHTU

CGATCTGCGTGGTCGGCGTCCAAGGCACACAGGGGATAGG

>HRA5KJN01A5NLO

CGATCTGCGTGGTCGGCGTTCAAGGCACACAGGGGATAGG

>HRA5KJN01BYINZ

AGATTTGTACCTGTATAGTAATTGTTCACCTGCAAATATTGCAGGTGAACAATTACTATACAGGTACAAATCTA

>HRA5KJN01AU3C5

ATATAAATTATATATAATATAATTTTATATACGATCTGCGT

**SPECIMEN 1 DESTRUCTIVE PREPARATION**

>HRA5KJN01BR7QQ

CTGCGTGTCTCCGACGACTACGTAG

>HRA5KJN01AW7O0

TTCCAGATACTTAACAATCTCCTCAGGGCGGCTATCGCCTAAACCATGTTGCAGGTAGTTAATAGCATCAAAGATTGCAATGCGATCATGCTTATTACTACGGGGCTGGTTAATAACCG

>HRA5KJN01BNW0P

CCGCAACTCGCGACCTCCGCGGGCGGGCT

>HRA5KJN01AIZYG

GCAGCACTGGCACAGGCTTCCAGCAGCAAGGCAGCAGGCGCAATCGCCTGCTGCCTTGCTGCTGGAAGCCTGTGCCAGTGCTGC

>HRA5KJN01A7ZX7

CACAGTGCGTGGTCGGCGTTCAAGGCACACAGGGGATAGG

>HRA5KJN01AJ8ZV

GAACCTGCGCATGGATAGGCGCAGGTTC

>HRA5KJN01A377H

ATACTTATGATTTAGAGGATATGTTAGGAGAAGTTAGCATACAATCGGCAGACGAAGTAGATGGTAAACATTTAGAAGGTTTGTATGCAGTTGTAGATTGTGACGGTATTAAT

>HRA5KJN01A975G

AGAAATGCAATCTGACCCTTAACAGCATTAATCGCTTCTTGTGCTGCTGTTAAGGGTCAGATTGCATTTCT

>HRA5KJN01BF3D5

GCATTGTGTCAGTATATAGCACGGCTCATAAGTATATACTGACACAATGC

>HRA5KJN01AN9DX

AGGATCTTGCGGGCTTTCTCGGACGCATCGTTATACCACGCATCCGCTTCATCGCTCTTGATGTCCTCGGGAGCTTCCCATGCAAACCACTGCGCGCCGGCGGGAGTGATGCCGGTGGAGTGGAAACTCGCCAGCGTGGAAATGCAGTCGATGAGCGTGGTATAACGATGCGTCCGAGAAAGCCCGCAAGATCCT

>HRA5KJN01BDA4G

AAATTACCTCACAAAATGCAAAAGTTTTCTAGAGTTGACTACCATCGAATTATCGTGTAGTCATAGGTTTTGCATTTTGTGAGGTAATTT

>HRA5KJN01AHYK4

CACCCTCGATCACGGGGTCAGTGGCCGATGTGAACACCTCAGAGCTGCCGTCGAGGTACTTGATGGTGACAGTCATGCGGGGTCCTTGTGCGAAAGGTCTTGATGGCCACTGACCCCGTGATCGAGGGTG

>HRA5KJN01BB8Y2

CGGCAAGTCGATGCGTGTTGTTCTAATTGGTCATGAGGGCATCTCCACTGAGCAGAACAACACGCATCGACTTGCCG

>HRA5KJN01BV2EX

ATTGACGTTGTTTACGGTTAGTTTTGGAATAACCGTAAACAACGTCAAT

>HRA5KJN01BE8D8

TGGGGCGAACAGCAAGTTGCCGATGGTCTCGATAGCACCACTGCCCATCTTGAACAACGGTGAGAGTGGGTTGTCCCCCGCTGCTGCGATGGTGGAGTTGGGGTGGAGACCATCGGCAACTTGCTGTTCGCCCCA

>HRA5KJN01AA3GZ

GCCGTTCGACCAGATCGCCGGCGGCCCGCACGCGCAGGAAGTCGGCGCCGACGATGGCGTGGGCGGCGCGGTCTAGCCGGCGGTCTGGTCGAACGGC

>HRA5KJN01BKLA1

TCATGTTTGCTTTACATCCAATGGGTATTCTTACATGACCTCAGATGAATGTCTTTCCATTAGTCTGAGGTCATGTAAGAATACCCATTGGATGTAAAGCAAACATGA

>HRA5KJN01BGYPQ

ATCATAGTCGGTCTTCCGGCTGTCTACATGTGGAAGACCGACTATGAT

>HRA5KJN01ANM2A

GCGAACTCGATGTCGCGCTTCAGCGACTTGATCATCTTGTCGAGCTGGTACGCTTTTTCATCATCGCGGCCAGCGTGCTTCACAGCCATCTGAGTACCAGTCACGATGCTGGTCTTGGTCAGAATCTGGAGGCGGTTGGCCTTGCGAGCGGTCGCGGTGTGTGTCGCCGCAGCAGCATCGTCGCCTTCGACCGCGTAGTTAGTACCAGCGGCGTCGAGGGCATCGGTCTGCCATTCATGGTTAGTGGCGATGGCCTTGACCCGAGGAAGCGCCTGCATGAGCGGGCATTCCAT

>HRA5KJN01BU9PT

AGTAGCACCAGTTGGACCTACAGGACCCTGCACTGTTGAGTTTGCTCCAGTTGCTCCAGTAGGACCAATAAGACCATTTGCTCCATTTGCTCCAGTAGCACCAGTTGGACCTACAGGACCCTGCACTGTTGAGTTTGCTCCAGTTGCTCCAGTGGGACCTGGAGGTCCAGGTACCGTT

>HRA5KJN01A4RQE

GTTCAGCTTTGGAATAACTGCCGCCCTGATTGCCCAACTCGC

>HRA5KJN01AXNGY

ACGCTTATTCAAGAAAAATAGAACTTGAATAAGCGT

>HRA5KJN01AE1CT

CGTAGTTGCCCAAGTAGTTAACCACGCCCGTGCACCTGGCAGCGTTGCGGCGGGCCACGGTGC

>HRA5KJN01A4EHA

CACGATCACCAGCTTCGACAGCATCCGGGTGGAAGCTGGTGATCGTG

>HRA5KJN01BKBWT

ACATTGCTGTTAGTAGGTAATCAGGCAGCAGGTGAGGCATCAACTCAAATGCTGCCTGATTACCTACTAACAGCAATGT

>HRA5KJN01A613E

GTTTCCGAGGATCTTCAGTGGCTCCCTGAAAGGGTAGCCACTGAAGATCCTCGGAAAC

>HRA5KJN01BXUEL

TTTCGGATTGCATTATCATCGAACTCGATCTTGATACGA

>HRA5KJN01A1IJ4

GAGTCACTTTCTGGAGCCACTCCGAGAACTCTGGAGCCATGTTGCAAATCTGCCCCTTTTTGTTTTTTTGTCTTGTTCGGTCGAATCCGGAACTTCCAGAACATGGCTCCAGAGTTCTCGGAGTGGCTCCAGAAAGTGACTC

>HRA5KJN01BDZMN

GGATGTCCTATGGCTTTTTTCATCCAATCCGCCAACGCTCGTTGTAAGCCGAATAGCGCAATGGGTAGGAGCCATTCCATCCTCAATCAATATAAATGCTACGGCATTTAATTCTGCTACAGGCGCAATCACAACTTCTGCAACTGCTGTTGGATCTGTAATGGTTGGTGATTTTATTGGCCCAACTGCTGTTGGTCCTTACGCAA

>HRA5KJN01BCRDU

GATCTCGTCCTCATCGAAAACCGCGACGGCCCCGATGAGGACGAGATC

>HRA5KJN01A70BW

TCTGCCGTCTGCGTCTACGGTAGATATGGTACCTCGTCAACGAGCGGCGGCGGCCATGTATCTGAAACTACGTGGCGGTACCAACAGTTTTGGCCAAATGGCCTTACCACGCTCGGTACAAGGGCTACGACCATCGCTTCCACTTCAGCTGAAATTGGTCGTCAAACGGATGCTTTTGTACCGTTGTTGACATGGGCGAACAATGATAATGTTGCATTCAAGACGAATGCGAATGTTGTTCATTCGCCGCTTCTTGGCGTTCGTTTGGCCGATACTGTTCCATGTCTCATGGTAGTAGTCGACGAGGTACATATCTACCGTAGACGCAGACGGCAGA

>HRA5KJN01B1BKV

TTCCTCGTCTTATTGATATGGGTGTTGATCCGTTCCTTATCAATAAGACGAGGAA

>HRA5KJN01AG5ND

ATAAGCTGAACAACGGCGAAGCGTTCGATGGTTATAAGCTTGTTGAGGGTCGCTCAAGTCGCGCATGGATAAATGAAGAAGAAACGGCGAAAGTATTATCTGAAAAGCTGTCAGCCGATGAGATGTTTAAGCATACGCTAATCAGCCCTGCCGCAGCCGAAAAACTGTTAAAGACTCAAAAGTCTATACTTGAAGGTCTAGTTATGAAATCCGAAGGCAAGCCCACGCTAGTACAATCGTGTGATAAACGCGATGCAATAAACAAAGCAAATGTGTTTGACGCACTGGATTAAAGCGTTTATTAT

>HRA5KJN01BK3BM

GTTGGTGACGCGTTGATGTAAGTTTAGGTTGAACTAAACGCGCAAGTCAAGCAAAAGCTAAACTATTTTCTAGTCGCTGAACTTTTATGCAAAAGAAAACCCGCCGAAGCGGGTTAGCGAGGTCGCCTTGAAATCAAGGAGACCTAGCTAACCATTCACGGTTTAGTAAGTCCTTGACGACGCAGTTTAGTTTGAACTAAACTGCGGGAATGAAGAAAACTCTCAAAACATGGCTTGAAGAAGAACGCGGGCGCGCAGCAGCGCTGGCCGAAGTTCTGCACGTGACTCCAAGCGCCATTACGCAGGCGATGAATGGAGAAATCCGAGTCCCGCCGT

>HRA5KJN01AJLEM

AATTCGGTCGCGCGCTCGATTGCCATCGCGCGACCGAATT

>HRA5KJN01BDNGY

CTGCATGGCCGAGATGCCGATTACACCCGTCATGGGCATCTCGGCCATGCAG

>HRA5KJN01B146U

TGGCGCAGTTCCTGGATCACGCGCTCTTCGGCGGCGTGGGCGTCGGAGCCGTCGGCCACGGACTGGTCGAGCGTGTCCAGGAACTGCGCCA

>HRA5KJN01A4LIP

GCTGTATCCGATGCTAGATAATCGGATACAGC

>HRA5KJN01A2Z8N

ATCCAATCAGGTCTCCAGTGAAATGGTTCGGGCCGAGTCTCGAAAACACTGAACGTCGGTGTTTTCGTTTCCTTTACTCTCATGAAAGTCCAATAACATGGAAGCATTGATTTTGGCGTTGCTGGGTCCGATCTTGGCGAAATGTCTGGATCAAGTTTCATCTGAAACCCCGTCCACACAGTGCGTA

>HRA5KJN01ALTC1

AGCATCCGGGCATTCAGGTTCACCGAGACGCGACGATGACGTTCGTGCGCCTGGCGTCGCGCTTCGGCCTCACGCCGCTCGACCGCGTGCGGATCGTCCCGGCCAAAAAGGAAGAGGAAGATCC

>HRA5KJN01AAQ0C

TTCAGCGCGGCACAGCCCTGCTCCAGCGCAATCAGCGAGCTGGCGCTGGAGCAGGGCTGTGCCGCGCTGAA

>HRA5KJN01ANC8S

AATCAATCACTTATGATGACATAGTTCTCAAAGCGATTGCGTTGGGGTTCTCATAACCTCGACCGCGAAGAAAGAGTTGTTCTTGAAGCGCGTGCGCAAACTTCGACAACTGGTTCAAGCGGCGGCTATGTGGTTCCTGTTACTTTGGCTTCTGAAATCGAGAAGGCAATGTTGGCAAC

>HRA5KJN01BM1GJ

CAGTAACTTTGAAAATTTGAATCGCTCTCATGAATCGGTGCTGAAAGCGCGAGCTCAAATTTTCAAAGTTACTG

>HRA5KJN01BBFR9

GGCTCGACCGCGCTGTCGCACCAGATCCTGATCGACAGCGCGGTCGAGCC

>HRA5KJN01BFD5B

GCCTCGTCAGCAAAGTAACTATCAAAGTTCTTTGCTGACGAGGC

>HRA5KJN01BDT01

GCGATCGATCGCCTTGCCGACCGTCTGCCGGAGAGAGTGGCCGGCAAGGCGATCGATCGC

>HRA5KJN01BTVDK

AGCCTTGATCTGCTCCGTCTCGCGGGTCATCTGTGGTTTCGGCGTTGGAACTTCTGCACATCGGCCTGCATTTCTGCGGTGTGTTTTTGCGCGTCGGCCTGGATCTTGATTTCAGCGACTTGCACCTCAATGGGTTTTTGCGGTGGCTGTGGCGGTGCCAGGCTCGGGTCTTGCACAAAGTTCTGCACATCCTTGAAGCCTGCGTTTTCAATGATCTTGCTGGCGCTGTGGTACAGGTGTTTTGGTGTAACCAGACCGATTGCCATGCCTTTTTCCTGAAGCTGCATGATTGCCATGAGCTGCT

>HRA5KJN01BUN5D

GCAACTGGTTTCGCGTCCAGTTGTCATTCATCTGGCTTGTGATGCCTTGCGCCTGCTGATTCAAGTACGGGTTTTGGCTCTGCTGTCCAGAGCCGAGCCCGAGATTTTCAGTCTGCGGCGATCCAGCGGGACTTTGTGCAACGCCAGATGAATGACAACTGGACGCGAAACCAGTTGC

>HRA5KJN01A7D6E

CCCATCTCTAGACGTGTTGCGTCATCATGAGCAGGCACAGGCG

>HRA5KJN01BKZQJ

GCTGATTTATGACATCATTTCAAGGTCATAAATCAGC

>HRA5KJN01BADTQ

CACAGTGCGTGGTCGGTCTTTCAAGGCACACAGGGGATAGG

>HRA5KJN01BVPD4

GTTCAACCGCACTTTGGACGTCTTTTACCTCTCCCACATAGCAGCCGGCTTTTGGCGTTTCTGTAACGGTCTCGACGAGTGGCCCGAGTTTGACTTTGACGCCTTTTATGCCTAACACTACCAGCTCGTCGACCAGGACGCGTAGAAACAATACTATTCGCCCGGCCTGCTCGCACAACGCGCATCCGCCCGTTTCTACCGCCTCCCGGACCTCCTCCACCTACCGGATTCGGACGGACCTTTCGGCCTGGCGCGCAACAAAACCACGGCCGGCGGCCATATTACCAAACTGCCCCGGTAGGCGCACAACGTCTTCCGCACCATCATGGCACAGCCGACCCTCCCCTTCGCCACTATAACGCGGCTGGCGCTGTCAACTTTCCGCCAAACCATCGCGCGTTTACGCAGCGACCACCCCCAAGTCCAGCCCTATTCGGAGACGCAGGCGCGCTTCTAGCTCGACCACGAGCACGAGCCCAGCACCGAGGAACGCCTCGCCCCAAGCCGGGTAGGCGT

>HRA5KJN01A5ZVB

AGGGACACGACTTCTCTTACCGCGGAATGAGTGGCACGCGGGCGAGCAGCGTCTTCCGGAGCATCGCACTTGCTCTTCTTCACCGGTACCGACACCATCCCAGCAATCGAATACCTGGAAGAGTACTACGATGGTCAGGGGCTCGTCGGTGGCAGTGTTGCTGCTACAGAACACTCATTCCGCGGTAAGAGAAGTCGTGTCCCT

>HRA5KJN01BMA8Z

GGAACTTATCATTCAAATACCATACTATCTTATTTCTCT

>HRA5KJN01A5543

ATGGTCACTTCCTGGTGGAACAGTAGAACCTTTTGGAAACACCTTTAGACGCTTTCATTCGTGAAGTAAAAGAAGAAATGTCAATTGATATTACCTATGGTGAGTGTTTTTTGAGATTAATAGTTAAAGAAAAAGAAGGTGATTGTGAAATTTTTTATTATGATGTTGAAAGTTCTAAATTTAATGAAGAAGATATAATTC

>HRA5KJN01BDVZR

CACAGGCCACAAGCAAAGTACAAGCAGCATTTAACGCGTGCTTGTACTTTGCTTGTGGCCTGTG

>HRA5KJN01AZR4P

CTCCCAGCGCAATCATTTCAGCTTGGGTTCTGGTATGCGGTCCGGCCTTGCGTGCTACCATCCGGTCCGCATACCAGAACCCAAGCTGAAATGATTGCGCTGGGAG

>HRA5KJN01AFP9H

CTCGGCCTCGCCCAGGGCAAGGGCCTTGCCCTGGGCGAGGCCGAG

>HRA5KJN01AAEUO

AAAAGCAGAACTCGCGCATAAAAATGGATTCAAACTTGGCCAATTAGCCAAGATAAATGAAGCCAGAAATGCCTCTGACCCTAAGAATATTGACCAGGCTCAGCCTGCAGTAGCATCAGGGAAACAGTCGCTTTATGATGTGTGGGCGAATGCTGGTAAAGGCTAAGCTATCCTAAGCGGGAGAAACTTAACTTTAAACCACTTTAAATAAAATGAGCCAAGTAGCAGGAATTCCCTCTGCCGGTGTCCAAACCAGCGAGAATTATGCTCTGATCAGTGGATATGATATCTACGACAGAGATTTTTACAAGAAACTGATAAAGAAGGTGCCGCGTGCGGCTGCTTTACAGTGGATGCGTGTAGTTAAAGGCTACATGTCAAAAAGAAAAG

>HRA5KJN01AJ2NL

CTACCTATGAGCCTAAAAAGAAGTTTCACACTTCTAAAGCCTTAGCTTCTATGCGAGAAACTGCCTTTGGGTCTGCAGCCTATTTTGACACCAAACGTACCCTTGAGTCGGCTGAAAGGCTGCGGGA

>HRA5KJN01BOPDN

CATTCAGCAGATTTTGGGTGCTTTCGATATTCACCCAAATCTGCTGAATG

>HRA5KJN01AP3N2

CGTACGACGTCGTCGTCGGTACGGGCCGTCCTACTACGGGAAAGGTACGGACACAGGGATAG

>HRA5KJN01BDY53

CCGAAATTCCTAACCCCTGATAGATGTTATTCAGGACCGGCTCGTACTTCTGACTTCCTAGGAACTTATACACCTGACTGTTTGACTCAGTGAATTTAAGATCAGGGCCCCATACTAAATCAAAGGTGCCACCCCCCACATTACTAGCTAA

>HRA5KJN01AK280

GGGCCGTATCGGCACGCCGCCCGGCATCAAGGGACAACACTGTCACTGACGCCGGGCGGCGTGCCGATACGGCCC

>HRA5KJN01A0TDW

AAGATGTTGCTTTTATGCCCGATGGAGCCTTGCGAGTTAACGACAGTAGCGCAAGGCTCCATCGGGCATAAAAGCAACATCTT

>HRA5KJN01AGSXD

CGTACTACTCGACGGTCGTACCGACGTACGGGTACTACGGAGGCACACAGGGATAG

>HRA5KJN01ANW7E

CGTACGTACGTCGACGTCGTACCGACGCGGTACTACGAAGGGACACGACAGGGATAG

>HRA5KJN01ADJQ6

ACTTGATGCCGGACCAGAGTCCGGCATCAAGT

>HRA5KJN01AY22W

GGCTGCCACGGTCGACCGGGGTTGGTGCCGACAAACGCCCGCCACAGCGAGGCGATCCGTTCGTCGGCACCAACCCCGGTCGACCGTGGCAGCC

>HRA5KJN01BA967

GCGTGGGTGATCGCGCCGCTCGCGGCCCGCATGCGTGCGGCCTACCCGGAGCTCGCGATCGAGATCTCGCTCGATGATGCGTTCGTCGACATCATCGCTGCCGGGTTCGATGCCGGGGTGCGCCTCGGAGAC

>HRA5KJN01BW65Q

GACAAGCTGATGGCACATTGTTTACTGCTTCTGATCGTGCCATCAGCTTGTC

>HRA5KJN01BCFBN

TGATGCGTTCCCATCGCGTGTTCTTCCGCCTCTATGATACACTCGATGGGAACGCATCA

>HRA5KJN01ASZ22

GTCGATCAAAGATTTATATAAAAGTTCATGGGCGAAGATTTAGTTTTAAAACATTTATATAAATCTTTGATCGAC

>HRA5KJN01ARI8C

GCACTGTTTTATATACTACGACTTCCGCTGATGCCGTTGTTATAAACAGTGC

>HRA5KJN01BVM9F

GTCGCCCGTTGAATGAGTTGTTATGTACCATTAGGAGGAATTATGTTAGCTGACGAATTAAACGGGCGAC

>HRA5KJN01ASD0W

AGCTCGAATGGCAAATCGTGGTACACGACAAGGTGTGGCGAGCACGTAGCCTAGTTGTTACTGACATGCAAAATCGTGGTACAGGTGGACGAGACTTGCCTTGGGCAGAAGTTTTTGAGACTTGTTATAACAACATCTACCCTTCTGTTACTGACGCCATTAAGTC

>HRA5KJN01ACEYA

ATTCTATTCGGAAGAGGGCGTACCTTGAAGCACCCGCTGCAGCGTGGCCAGCTTCTCCTTTTTCTTCTTCCCCGTTAACCCTGCGTAGATTCCGCCGCGCGCGTTGGCAGCGTT

>HRA5KJN01AFTUT

ATTAATTAACATTAACAAATTATTCTCACGTTTAGGAATATTTTGTTAATGTTAATTAAT

>HRA5KJN01AYTGK

GTTCCCTCAGCTTGATCCAAAGTCAACAAACTTTAATCCAAAGTTTTTTGAAGCGGTCAAGAACCAAGTCATCACCCAAATGATGAACGGGGAAAAGGACTATGAAAAAGCAGCTGCACAAGTTGCAACATGGTTCCCACTCCAAACGGCTCAACCTGTCCAGGATACGAAGAAAGAAGAACAACTGCGTCAATCGTCTGCTGGAACCCCTTCGAGTAGTCAAGGTAAGACTGCACCATCTGCACAAAAAACAGTGGATAGCGATCTTGTCAGACGTACTCAACTTGGTGATAGTTCAGCTTTGCTTGAGCGATTAACTCGAGCAGGTTACTAATTAAAGGAATACTATGCCAGTCGGTAAAGATACATACGTTGGTTCAAGCGAAATGCGTGAGTCGCTATTAGCGATCATTCGTGACGTTTTCTCCAAAC

>HRA5KJN01A3DJV

TGTCTTGGACGCGGGCCATGTCTTTCACAAAGCCCGCGTCCAAGACA

>HRA5KJN01AA9E3

TGCAATGGCTGGAACCATCACAATGTACGATAGACGACTTTAAAATACAACAGAAATGAACATCAAGTATCTCAAAAGGGTTCTGATCCCGCAGCGGGAGTTGGAAATCAAGAACGGAGACAATCTCTCAACACGCGACCCCATCTATGTGGTCTATTCCCTGATCGACCAGTATGTGAGCGGACATTCGGATTATTCCCCGACCACCAACTACCAAGGGAAGCCCCCTGAATCGGGCTATTTGTACCGGCACGACAGCTACTCTGAAACAGTACCATTCTATATGAACGAGGATGGCTCAGACTTGGAACTTGACGAGGATGATGCGTATTCAGAAGATTCCGCAGAGGAGGTCACCCGTCTTTGGGTTGATCGCCTAGTAGCCATTTTTCCTCACCTCCAAGGCGGCATACGACTACATGCAGTATCAGGGGCACAACCTCACCAACCCCATACGTTTACGTCCATTGCGCGGGTTTACAGGAACTATGAGATGGACATGCTGTCCC

>HRA5KJN01AW330

ACTGCGAGCGCATTATTTGCGCTCGCAGT

>HRA5KJN01AW0YX

TAGGACATGGAAGTCCGATGAATGCTATTGAAGAAAATCAGTTTGTAAGTGGTTTTAGAGAGTTAGCCAATACACTTCCGCAACCGAACGCTATTTTCTTCAATAGCATTCATCGGACTTCCATGTCCTA

>HRA5KJN01AX3FN

ACATCAAATTTAGCAGTTCGCAGCTAAATTTGATGT

>HRA5KJN01BZU0C

CAACGCGGATGCGCTGCACGCGCAATTCAACATCGTCCTGGGTGGCCGCAACAGACGAGGCGACCAACTTATCAATGTCCTTCGAACCAATGGCGATCTGCGCACCGAACTTGCGGCGCACGACGTCGTTCCAGGACAATGTCGAGCGGATGCGGTTCTTCATAGCGTCCACGCCGCCGCCAAGCTGCGCCATGAACTGCGCGTGCAGCGCATCCGCGTTG

>HRA5KJN01AIMWJ

ACGCAACTATCAGCCGCGTCGGTGGCCATGCTGACGACGCGGCTGATAGTTGCGT

>HRA5KJN01A7X86

AGATGTATTAATTGTAGATACCACAAACGGTGTTTTAGGAATAAATAAATCT

>HRA5KJN01BEENV

ATGTCAAGTGTCACTGTGCCAGTTTGACATTAGATGGGGGAGATGCTATACGTGCATGGGTGGGTGGGCAGGCCCGCGTTAGAAGTCCTAGTCCGCAGTCTGGCCTCACTCCCTCCCTTCCCTCTCCGTTGCTTACATTCCCCCATCTAATGTCAAACTGGCACAGTGACACTTGACAT

>HRA5KJN01BNKEK

AGTCAATGTTCGTAGAACGTTTCAATCGAACTTTAAAAGGGCGTATGTGGCGACGATTCACTGAAGAAAATACTCGTCGCTGGGTCGATATGCTTCCAGGACTCATGTCTGACTATAATAACTCAAAGCATAATTCAATAAAAATGACCCCTACAG

>HRA5KJN01B2S9B

TGTGATGTTCCGTCCACAAGAGATTATTCCTGACATTACTATTGACCTTAACTAAGGAGAACTAACATGACATTTGATCCGACAATCAACCGTATTCCGTTTGGTTTGCTTAGTGAAGACGAACAGACTGC

>HRA5KJN01BQ8B6

GCCTCCTTTCTATAAGATTGCTTCTGCTATGAAACAAGCT

>HRA5KJN01AQZT8

AGCTATTGCCCTGTTTAACGCCGCCTGGCGTTAAACAGGGCAATAGCT

>HRA5KJN01A3S3G

TGGCATGGGCGATGGCTCAGACCTCACCAACACGCTAAAGGCAACGGCATTCAAGTCTGGAACGCAAGTCACAGACGCTCAAATGACGGCGCTTCTGGTGGTGAGGTCTGAGCCATCGCCCATGCCA

>HRA5KJN01BINWM

CTGCGGCGATGCGACGCACGGGTGGCAACACCATCGCCATTGCCACCCGTGCGTCGCATCGCCGCAG

>HRA5KJN01AVSXN

ACTCACCTATGACGCTGCGCCCATGCTCGAAGCTGCGCACGCTGCTCGGGTGGCGTCAGAGGGTCATCGGTGAGT

>HRA5KJN01AGWFF

CCTGGTGGGTGCCGCAGCCGAACGGCTGCGGCACCCACCAGG

>HRA5KJN01AO17Z

CAGGAAGTCCTCTACGGAGGTGCAGCAGGTGGTGGTAAGTCCGTATGGCCTCATAGCTGATCCGATGCGGTACTTTACGAACGAGAATTTCGTCGGTCTTATTCTTCGTCGTACAAATGATGAACTTCGTGAGATCGTATCGAAGTCTCAGATGCTCTACCCTCGTATCTTCAAAGGTGCGAAGTACCACTCAGTCGACAAGGAATGGAGATTTCCGTCAGGAGCCCGTCTCTGGATGACCTACCTCGAACGAGACGACGACGTCATGCGTTATCACGGTCAGGCATTCAGCTACATCGGCTTCGACGAATTGACCCAGCATCCTACTCCCTAAGCTTGGGACTTCATGCGCTCTCGACTCCGTTCTAC

>HRA5KJN01AO5WS

CTGTTGATAAAACGACACTTTGTTCAAAGTCTTTAAGCCAAGTTGATTTACCCGCCCCTGAAGGCCCAACCATCAATGTCAAAACCCCTTGCTTATATTTAGGCGTATCAAAATCCCCCGCAGCAACT

>HRA5KJN01BMO5H

GCACAGCTACCCTGCATTTTGCCCGAAAGCTTCCAGCTCTTTTTGTCAGCACCTTTAGTGCCAGTACTGCCCGAAAGGTTCAAAACGATAGGCTTTAGTTTTGTACCCACAAATATCCGGTTGCCGTT

>HRA5KJN01B1SDW

TTGGCACCGGTGGCCACCTTCGCGTTGAGGATGTCCTGCACCGACTTGAAAACGCGAAGGTGGCCACCGGTGCCAA

>HRA5KJN01BDZ4A

AATGAATTTTTAAACAGTGGCGCAAAAGGTTCTGTTTGTAAAATTTTAACCTACTTTAAAAAGCCAACACTCCGACTGGAAAATATTGATAATTATTACGTTCCTTTTATCCATGAGTTAAACGAAAAACGACTGCTGGAAATTATGATTATCCATTTTACGCAGACCCAACACGCAAAATAAACGAATCAAATGTAATTTTAC

>HRA5KJN01BP4B0

CGAAATCCGCGACCTGGAGACCGCCGAAATCGCCATCAAGGCCGCGCAGACCGGTCACATGGTCGCGGATTTCGGCGGTCTCCAGGTCGCGGATTTCG

>HRA5KJN01A8W3H

GATCCGCGGCCTCGACGTCGAGGCCGCGGATC

>HRA5KJN01ADGD9

AGGCGACCCGGGCCGGCTACCCGTTCGGGGCTTTGTGGGCGCGGGTCGCCT

>HRA5KJN01A9OMR

CTTCCTGCTTGGCTTCGTTGAGCCTGCCCCATTACAGGTATTGGAGTGCCATTCCTAATCGGCGGGAATACACCAGCCCTAGCGCCTTCAAAACGAGTCGGGACAGGTGGTAATAGCGCTCCATTAGTTGATGTTAATGGGGCAGGCTCAACGAAGCCAAGCAGGAAG

>HRA5KJN01BBVJD

ATAGTATAGCGGCCGAAGTGTGCCAATCCCCGGCCCGAAGTGCGTGCGCAATATCGCCATGAGGGTCGCGCGCGTCGCCGTTGTTTGCTTCCGCAATGCCTGACCGCGCGAAAGGAATGCCAAAGCTGTTGCGCAAGACGTAGAGAGTGCCGCCGGGGTCCACCACGAAAGCAGACTGGTCCATGTAAACACGGCGGGTGCGGCAATCCATGAGGACGCCAGCCCCGCTTGTGGCTGGGGTGAT

>HRA5KJN01BTIBX

GAACATACCTCACTACCAATACGGATATAACCACTCGCAGGATAATCTAGTGCGCCTACGCCTGTTGGTGTCAATGTTGCCGTTGTTGCGCTGTTGGTTATGTCTGCGTTAATTTTGCCGACACTTGGTAGTGAGGTATGTTC

>HRA5KJN01BHO1P

ATCTCCTTGGCGACATGCTCCAGCGCCAGTTGCCAAGGAGAT

>HRA5KJN01AW32V

CGTACGACGTCGTCGTCGTACGGCGACTACTACGGAAGGTACGACACAGGGATAG

>HRA5KJN01A0PX6

ACCTACCAGCGGCCGAAACAGACCCGGGCGCAGGCGAACAAGGATCTCCTCGAGTCGGGGATGATCGACCAGTTCGGCATGATCACCTAACCAGGAACAAGGACCACCCATGTCTAC

>HRA5KJN01AF24L

ACTGCGAGCGCATTATTTGCGCTCGCAGT

>HRA5KJN01BIJZU

CTGTAATGGAGGGCTTTTTGGTATAGTTGATATATGACAATCAAAAAGCCCTCCATTACAG

>HRA5KJN01AP38I

CGTACGACGTCGTCGTCGTACGGCGTCTACTACGGAAGGTACGACACAGGGATAG

>HRA5KJN01BXZNQ

ACCCAATAGGTTTAGATTTTATGGTTATATTAGTGGGTGCACCTGGTGCAGGGGTGCATTTAATGCAGGGGGTGCACCAAATGCACCCATGTGCGCGGTTTCTGCGTGATTTTCAGCCTGTTTTTGACGCTGTTTTGACGGTTTTGCGCGCTCTGTTTGCCATGTG

>HRA5KJN01AOY2Y

CTTGAGCTCCAGAGCATAGGCAGCGGCCACAAAGGTGGCCGCTGCCTATGCTCTGGAGCTCAAG

>HRA5KJN01BK9QY

ATAAGATCATGATAGGTCGCTTTTATTTTCGAAGCAGAAATAAGTGACCTATCATGATCTTAT

>HRA5KJN01BYIKB

CTGCAGGTTGGCCACGACCAGCGTGCCTCGCTGGTCGTGGCCAACCTGCAG

>HRA5KJN01BCX2U

CAGCACCACAGAGGCCACTTGCAACTCTGGCACTCGCGGCCAAGTCGTGATTGTCCAGGGAGG

>HRA5KJN01ALWSF

AAAAGAATTGATATCTTTATTTATAAATTTACAATTTATAAATAAAGATATCAATTCTTTT

>HRA5KJN01AB57B

GGGCCGTATCGGCACGCCGCCCGGCATCAAGGGACAACACTGTCACTGACGCCGGGCGGCGTGCCGATACGGCCC

>HRA5KJN01BPOO5

GCAAAGGAAATTTTCTTTAATATCCAAAATTTTCCTTTGC

>HRA5KJN01B0JP1

AAAGTTGTTCTTCATCGACTTCAGGTAGCTTGGGGTCGTAATGCGAGAACAACTTT

>HRA5KJN01AJ444

TAATGATGACAGTAGCAGTTTTGATATTTCATGCAAAACTGCTACTGTCATCATTA

>HRA5KJN01A8GD4

GGGCGGTGCCGTCCGGCGCGCCGGTCGACGGTGACCCGCTGACGGCACCGCCC

>HRA5KJN01B02D7

AGATTCACGTCACGGGTGGCCCGGAAACCGCGAAGGGGCTTGCCTTCGCGGTTTCCGGGCCACCCGTGACGTGAATCT

>HRA5KJN01BKKZX

ACAACATCGTGACGGTGACGCGTGCCGGTGGATCCGCGCAGCGGGTAGACAACAGCGCCGCGTCACCGTCACGATGTTGT

>HRA5KJN01BUXX8

AGCTTCTGGTATATTAATAATTGGCGGTATTGTACAATTATTAATATACCAGAAGCT

>HRA5KJN01BEBZ5

AGAAAACAAGGTGCCGCATACTTCCTAGCGACGCATTTGCTTGTAAAGGCACCTTGTTTTCTGCACAGTGCGT

>HRA5KJN01AMEVT

GGTTTATTCCAGCTTTCAGGGTTCGGCACAGAAACCCACTTGATATTTTCAGAGTTCATTGGTGCTCTCCGGCGGCTTTGGCGATTGCGGCGCGGGCTTTGTCTGCTAAATGGCCGGTCCAGTCATAACAAG

>HRA5KJN01AEBOQ

GTACACGCGTCGGATTGCTGAGTTGGAAAACGAAGTGTCACGGCTGCAAATGGAGCTGGACGCATCGTGTAACGCCGAGGAAATGCGACAGGTCCGCGAGCGCGTGTAC

>HRA5KJN01BBL3U

TTTTCGAAGACAATGACGTAACCAATGATTTCGTCATTGTCTTCGAAAA

>HRA5KJN01A2NLX

GATGGCTTGGTCAAGCTCCATGGCGGAACCAAGCCATC

>HRA5KJN01BGPT6

CCTGAGCGCCATGCCGAGGGCGCTCAGGATATAGTGCGT

>HRA5KJN01BBPID

CCTGACCGGCAACTATCGCGCCGGGTTCACCGTGCTCGCGGTGCTGGCCGGGATCGGGTCGCTGTTCTTCATG

>HRA5KJN01BUX5P

GGCAGCAGGCGTTGCTTCCTCCCGCGGCAAGCGAGTTCTCGTCTTGGAACGCAATGTAAAGATGGGAGGAAGCAACGCCTGCTGCC

>HRA5KJN01AW8OM

GGGGTGCTCACCAAGCCGTTTGATCCAACTATCCTCGCCAATCAAGTCAAAGCGATTCTCGAGGGCTGATTCTAAGATGCAGGCCTGGTCGCAGACTTTCGGTCAACTGAAAGAACAATATATTCGTCGTTCTTCAGAGCGCATTGGCAAGATTGTCGAGGATAGTTGGATCAAACGGCTTGGTGAGCACCCC

>HRA5KJN01BE12S

GAACAAATTATTTAAATTTTATTAATTGAATAAATAATAATAAAATTTAAATAATTTGTTC

>HRA5KJN01A15PX

ATCCACATATAAGCTTCTTCTAATTTGGTATGTACCAAAGCTTTAGCTCGTGGTGATTGAATTTGTGCATCAATCGTTTCTTCTAACTTCATGAGCGTATTTTTAAAATCCGCTTGAAGTCCATTTGCTTGATCGTCGTACTTTACATAATCGTATCTGTTTCCCATTAGTTCATCTCCATTTTTTATATTTAGCTACACAATCGGGACAATCACATTGAGCGTTCTCACCGTTTTCTAACATACTTAA

>HRA5KJN01AB19O

TCAGCTCGGCAATCTTCCGCTCCTTCGCGGCACGCTGTTGCTTCACATCTGTTGGCTCCTTCGGCTTGAACGAGTCGCTGATGCGCTCGGGTCCGGTGGCCGGCACGGGGTCTGCGGGGCGCTGAAGCATCGGCGGGACCATGGCGGGGGCGGAAGATTGCCGAGCTGA

>HRA5KJN01A9FF2

CCCACATTGACGTCTACCGCCTGCTGGAGATGTTTGGCGTCAATGTGGG

>HRA5KJN01B0DZW

CACGATCATCGCCCTGCGGGAGTGGCTCGAGATCCGGCCGGAGGTCGACACGCCGGCGCTGTTCGTCGGGCGCAAGGGCAGCAGGCTGGGACGGGACGCGGCCTACAAGATGATCCAGCGCCTGGCGAAAGCCGGCGGCGTGGAGGGCA

>HRA5KJN01AH68O

ACTCAAGGTTCGATCGGTTGGCTGGTAGACCATTCCAGCGGCGGATTGTGTCGTCATCCGATAGCCCTAGTCTCTTTTGCTCGGCGGTCAGGAATTTTACCAGGTAGGCACTGGCTCACTGGTAGTCCTCGGCACAGGCACGAGTTACTCCTGGGTGTATCCCTATTCGGATCTGGAAACATCCGTACGCTGGGTAGGGGGT

>HRA5KJN01AQGTV

GCTGGACGGCATCGCAGCCGACCTTGACGATGCCGTCCAGC

>HRA5KJN01A88KC

GATACATATACATATATATACATACATACAGTACATATATATATATATATGTATGTATGTATGTATATATATGTATATGTATC

>HRA5KJN01BA85T

GAGCCTTGTATTGTGTTCGGGAGCGGAAGTCAGACCAGGGACTACACCTATGTCTCGGACACAGTACAAGGCTC

>HRA5KJN01BERWZ

CACGAGTCGTCGTCGGTACGGGCGTCTACTACGGAAGGTACGACACGAGGGAGTAG

>HRA5KJN01AH93H

AGGCTGGGCTGAAGGAGCTCATCGGCTGGCACCCCACACTGCCTCTCTTCGGTGTCCCGTGGCGTGAGTCCTGGACAGAGCCCGGGGATCCCATGAAGGCCCTGGAGGGGATGCACACCGGAGCGATCGAGCCCGGGGTTCCCGTGTGGGACGCCAGCCGATGAGCTCCTTCAGCCCAGCCT

>HRA5KJN01ATYHP

CACGTAGTCGTCGGTTCGGGTCCGGTCCGTTCCTTCCTACGAACGGACCACCAACGGGAAGGGGTAAGGTAGG

>HRA5KJN01AHYDT

GTCGATCAAAGATTTATATAAAAGTTCATGGGCGAAGATTTAGTTTTAAAACATTTATATAAATCTTTGATCGAC

>HRA5KJN01AVKGY

AAGCAGCCAAACCGAAAAAAGAAGCGGACGTCAAGACCACCGAAAAGGAGGCATAAGCACCGCCCAATAGTCCCCATAAAAAGCCCACACACACAGTGC

>HRA5KJN01A3CQ5

TTAATAAGCTAAAATTAATTAATAATTAATTTTAGCTTATTAA

>HRA5KJN01ANC8I

GACTTCCTGGAGCAGGCGGTCCCAGAGGAGGTCCGACCACTCTGGGACCGCCTGCTCCAGGAAGTC

>HRA5KJN01B1152

CACAGTGTCGTCGGTCGTCGTCTCTACGACGACGACGACGGAGGTAG

>HRA5KJN01BV5W3

CGTACGACGTGTCGTCGGTACGGGCGTCTACTACGGGAAGGTACGACACAGGGGATAGG

>HRA5KJN01BCBX2

ATGCGACAATTGCGTCGACCACGGGATAAACCTCGTGGTCGACGCAATTGTCGCAT

>HRA5KJN01BHKT3

GTCTTAGCATCTGCAATTTTCTCAATATGCTTTTGTTTATTTCTCTATTT

>HRA5KJN01AQQQO

GGCGGCTGCGCTGGTTGGTCCCGACGATCCAGCGCAGCCGCC

>HRA5KJN01B221T

AATCACAGAAGTTGTACGTGTTTCCGATTATTGAAACCTCAATAATCGGAAACACGTACAACTTCTGTGATT

>HRA5KJN01BF58H

GACGTATCCTGTATGAATTGTATCGCTTGGCAAAGGTGGCCGATGCAGTCACTCACGATGTTGTTGGAAGACAGGCCGAGCACAAAGGTAAAGGCATTGTCGGGGCTGCGCGTCACCTTGGTCATATAAAGCAGGTTGGGGATCACGGCCTCGCCG

>HRA5KJN01BJ7E3

AGCCAGAATTCATTCCGACACAATACTTTTTACGCAGGGGCTCGATGCTTGGGAATCCTTGTCCCTGCGTAAAAGTATTGTGTCGGAATGAATTCTGGCT

>HRA5KJN01B1CT1

CTTCCTGCTTGGCTTCGTTGAGCCTGCCCCATTACAGGTATTGGAGTGCCATTCCTAATCGGCGGGAATACACCAGCCCTAGCGCCTTCAAAACGAGTCGGGACAGGTGGTAATAGCGCTCCATTAGTTGATGTTAATGGGGCAGGCTCAACGAAGCCAAGCAGGAAG

>HRA5KJN01BH4PS

CGTACGACGTCGTCGTCGTACGGGCCGTACCTACCTACGGGAAAGGTACGGACACAGGGATAGG

>HRA5KJN01AVNRQ

AACTTATCATTCTTTTCCTGCCCAAGTAATGGCCCAACAAGGTCTATGAATCCCTTAACATCAGCGGCCTGGAATGGGTTACCATCTAGCTTATTGCCAGCTCCACGGAATTGTAGGAATGCCATAGCCTGCTTCATAAACTGAAACTGTTGCTGTGGTCCGGCAGTACCT

>HRA5KJN01BZ4HK

GGTGAGCCTGCAGCCGAACTCCGGCGCGCAGGCTCACC

>HRA5KJN01A1MRH

TTGTAATTATCGTTGAGAATTATTTATTCTCAACGATAATTACAA

>HRA5KJN01BKH00

ATTCACGTCTCACGTCCGCCCTCGCTCATGCGGACGTGAGACGTGAAT

>HRA5KJN01BXJPO

AACGCAAGCGTTCCGGTAACCGCGGCCCACAACCACAGACTCAAACGGCGTCCTAATAAGGGTCCTCAAGTAAAGCGCCCGTATGCAAAGCGCGTTGATGAGGTGAGTGCTGGCGGTTACCGGAACGCTTGCGTT

>HRA5KJN01AYI99

CACAGTGCGTGGTCGGCGTCTCTCAAGGCACACGAGGGGATAGG

>HRA5KJN01AGJOM

GGTCCGATTTCAGGGACGGGACCTTCTCCATGAGGTTCTTGAAATCGGGACC

>HRA5KJN01BJ5AP

AGATTTATTGACTCCGTACCTTATGGTCTCGTTTGATGGTAAGATTGTAAATGACAGTAAGGTACGGAGTCAATAAATCT

>HRA5KJN01BLSWE

CGGGTATCGCCCCACGGGGTCGGGTGGCGATACCCG

>HRA5KJN01A4K5H

ATAGACCAGCTCGGCAGAGATCAGCAGCCGGTCGCCGCAGAAAATACCCGCCGTGAACAGGCACGACGAATTGCCGAGCTGGTCTAT

>HRA5KJN01AHT31

ATGTCAAGTGTCACTGTGCCAGTTTGACATTAGATGGGGGAGATGCTATACGTGCATGGGTGGGTGGGCAGGCCCGCGTTAGAAGTCCTAGTCCGCAGTCTGGCCTCACTCCCTCCCTTCCCTCTCCGTTGCTTACATTCCCCCATCTAATGTCAAACTGGCACAGTGACACTTGACAT

>HRA5KJN01AQWM2

CGTCCCCGAACCGCTCCGCGCCCTGGAACTCGTAGCTCCAGGGCGCGGAGCGGTTCGGGGACG

>HRA5KJN01ABJUW

TGACGCTCACCACGCAGGGAGTGATCTATGGGCCAGACCAGACCTTCGCCACCACTGGTCCACCCTTGGCCATCACGGGCTCCTATGCTGTGGCTGGTGTTAGCAGCTTAGCGCTGGATGGCCCATAGATCACTCCCTGCGTGGTGAGCGTCA

>HRA5KJN01BFXFJ

CTGGGCCAGCGTGGCGATTGCGTCCAAGCCGCTGACCGTGCGCGTCATAGCTGCGCTTGGCAAGATCAAGCTGGTCTTG

>HRA5KJN01B2KY9

GATTGGATACTCGATCCTTTCTCTGGAAGTGCAACCAGTGGTGTTGCACTTCCAGAGAAAGGATCGAGTATCCAATC

>HRA5KJN01B1R6X

GCTGATGGCGGTCTAACCAGATGAACTCGCCATCAGC

>HRA5KJN01BN8OQ

ACACGAGTGTCGTCGGTCGGTCGTCTCTAGACGACACACGAGGTAG

>HRA5KJN01A48BY

ACACCATAGGTAGTGTGGGAGTGGATGCTAAACCCTATAGGTAGTGTTCACAATGCGATTTTTGGCTTTTCAGACTTTCATTGATCTGGATCAGACGCAGGGGTTTTGACTATCGCACCTACCGGATGCGCGGTGGTGCCGAGAGCTGC

>HRA5KJN01A8CXY

CGGTGCTCGACCGTGGCGCGGAGTCAAACGCGATAGCTGGGAGGGCGGGCATCGCGTGCCGTTTCTCGTCCGCTGGCCCGGCAAAGTGAAGGCCAGCACGACCAGCGCGCAGCTCACGAGTCTCACCGACGTGATGGCGACGGTCGCGGCGATCATCGGCGCGAAGCTCCCGGACAACTCCGCTGAAGACAGCTTTAGTTTGCTACCCGNGCCACGGTCGAGCACCG

>HRA5KJN01AFOD6

GAACTGTTACCCCAAGGCAAGATGCAGCGTTCGATAGCTGCAATTCAGACCAAGATTTAAGCCAAAAACCCCATGAGATGCCCCTACCCCATGCCCTATCGATAAACGCAGCTAAAAACCGGGTTTCCAGTGCCGTGCCGGGTCGATTTTCAGGCTATCGGAATG

>HRA5KJN01B2GPT

TCTCCTTCAGCGTCTAAGCATCCTGTAATGATGCTGTGCTCATCTGTAGGATGCTTAGACGCTGAAGGAGA

>HRA5KJN01BU7MX

ACGCACTGCCGTAGTACTGCCAGCTCCAGGTGAAATTCGACAGCTTGACAAACCGGGCCGGGTGTCCCCAGAGCGTCGCGTCGTTGACCGTGTTGACCATCGCCTCGACGGTCGGGAGGTTGAGGGCGATCACGTTCTGTTCGATTAC

>HRA5KJN01A5TUA

TTTCGGTTCGTTTCCAAATTAGAGGACGAGAGTTTATTGTCGTAGCATATCCTTTATTTCCACTATGAGAATATCCATAAATTTCCAATATATCCCATTGTTCCCATCCGATTGTTAGGAATGTCAAGTCTTTATTGTAGTCAAAATTCATACAAGAGCTTGAATCTTCTACGTCATATAGAGCAAGTTTTTCATTTACTATTTGTTCAACCT

>HRA5KJN01AXQAA

GCGTCGATACGAAGTATCGACGC

>HRA5KJN01AKKWG

GGCACCACGGCCAGTGCCACGAGCTACACAGCGCAGATCCCCGCCGGCGGGTATTACGAAGTGCCGTTCTCGTATAGTGGCGCGATCGATGGGATTTGGGCATCAGCCACGGGCAATGCTCGCATCACGGAGCTGACGTAAATGCCTCTCTATGCACCCTCCATAGCAAACGACGGCGCGAATGGCGTGCTGTGTAGCTCGTGGCACTGGCCGTGGTGCC

>HRA5KJN01A8PG1

GTGCGGTCAGGTTCGGGATACGCCATGACTTCAGCCAGTTCCTGCCGTATAGCTTGAAGTCATGGCGTATCCCGAACCTGACCGCAC

>HRA5KJN01A323T

AGCTAAACGCTTGTAGTAGTGTGATCGTGTACACTCGGCGAATACTACAAGCGTTTAGCT

>HRA5KJN01BERTS

CCGACAACCGTCGAGACGATCCGGGCGACCATCGACGTCACAAAGTTGCGGGCCGCCCGGATCGTCTCGACGGTTGTCGGGCACAGTGCGT

>HRA5KJN01AS3J6

GTGGCAATCCATTTGCGTTTTAATTTCCGCTCGTTCTTGAAAAGCGCCTGATCGTTCTCATGCCTCGCGTCCAGTTGTGCGATCATTAGCTTATTAGTTTCGTTCATAGCCTCATTAGCTTTAAT

>HRA5KJN01ABFO9

GGCCGACCCGGAAGAGTTCATCACGGGCGCGGCGCCAGGCTTCATCCACCTC

>HRA5KJN01AD5RK

TACCTTGCGACGCTGCTGCGCTCGCAAGGTA

>HRA5KJN01A7ONB

AGGGTGCGAGGTCGTTCGGCTCGCACCCT

>HRA5KJN01BG5Y7

CACTCTTAATCACGATCATTCGCAAATATAATATAAA

>HRA5KJN01ASAM2

ATCAAATTATATGTAAATTTATTATTAAATTTACATATAATTTGAT

>HRA5KJN01ATGQT

GATTCCACGCGCCGCGCACGCGTCGCGTGGAATC

>HRA5KJN01BTZE7

ATAACGGGTTCATATCCTAATGTGATGGGATTGCCTACGCATTTAGTGTTTGATTTTCTTAAAAACTTTTAATCTTGCTCATCTGTTAAAATATAAGTTTAAAGTGGCTGTGATGCGATCGTTTGTACTTTAATCATTTAAAAAAGTAAAAATATCGGCAGCTAACGTGTAAATTCTGTAACGTATTAAGGGATT

>HRA5KJN01ALVK0

CTACGACGTCGTCGTCGTACGGCGTCTACTACGGGAAGGTACGACACAGGGATAG

>HRA5KJN01BQC0F

TCTTTTTCTTTCTCGAATGAAATTAAATCTTTACCAGTAAAGATTTAATTTCATTCGAGAAAGAAAAAGA

>HRA5KJN01A67MB

CCCGCTCCACCGGCGTGAGCTCCGCCTTGCTGCGCTGCGCGGCCTGCTCGAACATCTCGCTTTTGGCGAGCTCACGCCGGTGGAGCGGG

>HRA5KJN01AGZVS

GGCGCTCTTGGTGGCGTCGTCGGCGTCGTCTCCAAGAGCGCC

>HRA5KJN01BHB7U

CCAGGTCGTTCATAGCCCGCTGCACATCTGTTTCATCGCGCGCCAGGTCGTATGCCACGCACGACAATTTCGGCCATGGCCGGGCGGTATCGCAGCACCATTCGGCATAGGTCAGGATGGCGTGATGAACGGGGCGGATTTTCA

>HRA5KJN01BEXZC

ACACAGTGCGTGGTCGGCGTCTCCAGGACGCACAGGGAGTAG

>HRA5KJN01A3M8Y

CCGAGAAGAACTTGAAGTCGGCGAGGAGGTCAGCGGAAGCGAACAAGGTCATCGGACCCAGCCCTGCGCAAAGGCGATCGCGGCGGTCGCCCGACG

>HRA5KJN01AM4XC

CAATTAATGCTGTAAACAATTTCACAGGATTAGGTAATGCTCTTGAGATTGTAAGATACAAATTGACGTTAGCAAGAAATGAAGGTGTTGCTTATAGTGGTGCAATGTTAAGCGCGTTTAATGTACTAGGTGCAGCAGTAACTACTTTTGCTGCAGTAAGTATTGCTGGCCTCACTGTAGCATTAACTGGTCTTGCTATTGGTTATTATAAAGTAATG

>HRA5KJN01B03GN

GATGGCTTGGTCAAGCTCCATGGCGGAACCAAGCCATC

>HRA5KJN01AP4DP

TGCACGATGTTGGTCGAATTCGTGAAGCCATCCGCGAAGATTGATGAAAACCCAATTACCTGAATGGGCG

>HRA5KJN01ANJ7L

TGTTGTTCAAAAAGATCGTCTGTTTTGGCAAACTCCGCTGTGTTCCCGCGCACCCCAGAAGCATACTGCTTGGCAGCAAGAACAGCACTAGCAACTGCATTTTGTACCAACTCCTTCTTTGCTTCTTTATGTTCGATAGCAGTTGCATATTGGTTGGCTTCCTTTTGTTCACGATTAGCTGTTTTAATCTCTTTAATCGCGTCCATTATCTTTGGCAAGGCTTGCAACAGCGCCAATATTACTGAGATAATAGTACCAATCATATACGGTTATTGTTGTATCGTACCGC

>HRA5KJN01A3Z30

ATCAGGTCGAATTTGTACTGCGTGTAATCGGCGAGTAACGCCGGTCTTGGCAATCGGCCAGTCCATCGTGATACGGGTCGTCGTGACCCCATCCACGTTGATCTCGGCATCGGCGCTGGTGACGGTGAACAGCAGCGTGTCTTCCAGGTCGCGCACTTGGCATTTGAGGGCGAAGCCCGTCCAGTCGTCCTCAT

>HRA5KJN01BS2WL

CGCAGCGCGACATCTTGAGCGGTAGGGACAAGCCCTCGCCGTGGCCCTACCGCTCAAGATGTCGCGCTGCG

>HRA5KJN01BTL3I

TCCTCTGTGGAGAGCGCGACCGGCTTGGATGTGGCAGCAGCGGAGCGCTCTCCACAGAGGA

>HRA5KJN01ANWV1

ACTCATCATTATTGGCGGCGATAACGGTGCGGGTAAAACCACTATCGCACTTAACATCGTGGCTCATTATGTGGACGAACGGGTTCCGGTTTTGTTGTGGAGCGGTGAACAGCGTGTAGAAAAAATCCGTAATTGGTTTGAACGCATCGTGGCAGGGGAAT

>HRA5KJN01ARSPF

ATCGTCAAGCCAGCGTCTTACATTGATGTTTTGCATCAATGTAAGACGCTGGCTTGACGAT

>HRA5KJN01B1B81

CTCTTGGAGCGATGGAATATGCAGGACATTCAACG

>HRA5KJN01BLZHW

CGCCTGCAAGTTTGTAGCGCGAATCAAGGCGTTGCGGCTGTCCAAGGCCAAAATCGTGTTGGCAGGCAGCGGGCCACCCTCAGCAG

>HRA5KJN01AYCYP

CACAGTGCGTGGTCGGCGTCTCTCAAGGCACNNCAGGGGATAGG

>HRA5KJN01BHRBB

CGTACGCGTGTCGTCGGTACGGGCGTCTACTACGGGAAGGTACGACACAGGGGATAGG

>HRA5KJN01AITQM

GTAGTACTACACCTACCAGGGGTAGGTGTAGTACTAC

>HRA5KJN01BCK2R

GGGGTAATAGCGGTGGCGCATCGTGTAGGAGGCTGACACTTGACAACACCGTCGAAGCGGTCAACCGTCCTGCAATCCGGGGAGTTTTTTTGTTTTCGCCTCGGACTAGCGGGGCCAGGGG

>HRA5KJN01ATSVQ

AGCGTGTTGACAGACCCGACCATGGGTGGCGTCAGTGCGGGCTTCGCATTTTTGGG

>HRA5KJN01BOIZM

TTGGTGTAAATGTTATGACTCTGCACAAATCAAAAGGTTTGGAATTTAAACATGTTGTTATTGGTCACATGAATCAGGAAACTTTTGATTTGTGCAGAGTCATAACATTTACACCAA

>HRA5KJN01AFHVB

ACTGCTTTACACCCGGCATGAGACCCAGTTGGAGTGGGCCGAGCCGGGTGTAAAGCAGT

>HRA5KJN01A77N2

GTTCCAGCCAACACCAGAGTCGGTGTCACAAGGATCGCAATCGAAGTCATTTTCATTTCATATGCTTGGATTTTTTTACCCTAGATATTCAGGGGTACGACCGATCCTTGTGACACCGACTCTGGTGTTGGCTGGAAC

>HRA5KJN01AXXA2

AGGTTTCGGCGCCAAGTCCACCGAGAAGCTGTTTGCGTCGATAGATTCACGCCGCAGGATTGCGCTTGATCGCTTCATCTACGCGCTCGGCATCCGCCACGTCGGCGAGACGACGGCGCGCGATCTCGCCAAGGCGCTCGGCAGCATCGAGGCGTTCCGCGCCGGCGTCGAGGCGGCC

>HRA5KJN01AKUED

ACCGTGAGTGTCATGCGCCGCGCGGTCCATACCTTGAAAGGCGCCGCCGCAATGACGGGCGTCAATCTGTTGGCTCAGGGAGCCCA

>HRA5KJN01AXZ2E

NTCGNGTCGTCGTCGACGGTCTCTACAGGGACTACGACAGGAGTAG

>HRA5KJN01BOVMQ

GAACCTGCTCCGTGGCCGAGAATCGCAGGCATCGACTTGGCTCCGGTGGCAATCTTGGCGTCGTGAATCGACTTGACGGTCTCGAAAGTGTTGGGAACGTCGTTAGAGC

>HRA5KJN01B2DQV

ACGACAGTGCGTACGTAACGTCGCTACTNTAAGTACACACAGGATAG

>HRA5KJN01B26IX

CGATGCGCACCCGATGGCGGTCATGACCGGCCTGGTCGGCGCGATGAGCGCGTTCTATCCGGATTCGATCAATCTTGCCGATGCCCGTCAACGCGAGATTTCCGCCATCCGCCTCATTGCCAAGCTGCCGACACTGACCGCCATCGGGTGCGCATCG

>HRA5KJN01BLGRM

AACTGATTGCGGTTGAAAAGCACGAATTGGCTCGGAGGGGCCGCCGATCAACTCGTAGATGGAATCTACGTTTTCGTAGCTGTGCAGGTCCATCACAGCATGTATCCCGCGGCTATCGATCAGCGACTTAATAGCCAAGGGCTCGGCCT

>HRA5KJN01AI6XR

AGCAAACGAGCCATAACGGCCAGCACCAGACATGGCCGAGTTGACCCGATTGCCGATGCGAGCGGCATTCACGTCAAGCATCTCGTTCAGGTGCGGGTTGTTGCCCGCCGTTATGGCTCGTTTGCT

>HRA5KJN01BWIN4

ATGATACGCTTCTGAATCTCCACCGACTCGATGTCAGCAAACGCCTTGGAGAACGCGGGCTCGATGTTCAGCTTTTGCATCAGCTTCATCGTCATGGTGGCGATTCAGAAGCGTATCAT

>HRA5KJN01AO2KA

TCCAGCAGTCGAGTCTACCTTGGCATTCCAACCAAATCGAACGATCCAGCATTTACCGGACAGCCAATTACCAACGGCTCGTATGTATTCCCGTGTCGGCTTACCGACTCCAGTCGCTTGCCTGTGCACACGCTCAAGGTGCCTTGCTGGTTGCATCTCTCCAGAGAAAACAACTACGCGCTCTCCCTGCTCCATTAGCC

>HRA5KJN01BELSX

CGTACGACGTGTCGTCGTACGGCGTCTACTACGGAAGGTACGACACAGGGATAG

>HRA5KJN01BGLPE

TTAGAATTATTTTATGAATTAATATAAAAATTTTTATTTATTATTATTAATATTTTTAAATTTATTTTTAATTCATAAAATAATTCTAA

>HRA5KJN01A8C1C

CAACGCTGGCACCGTCACCATCCCATGCAAACCCGGTGATGTGATCG

>HRA5KJN01A4RAB

ACCTTTGTATAATGTACTGCCAACTATCTAAGGCATTGGCATTGTAGGGGCTGTTCGGCAGTACATTATACAAAGGT

>HRA5KJN01A4CN9

AAATTCATTTAACCACTCCATGTTTTTGGCAACGCTTTTTCTATAACGTTGGCCCTTTGGCATTCTATGTTTTACTGCATGACAATTTGCACAAAGAACATGGAGTGGTTAAATGAATTT

>HRA5KJN01BPKII

CGCGAGCAAACAACGTAATGTTTGCTCGCG

>HRA5KJN01AM0O4

AGGCCCAGACCTGTTCGCCTGCCAGATGCGGCAGGCGAACAGGTCTGGGCCT

>HRA5KJN01A32CF

TAAACCAAGATACAGGAATAATCAAATATGATTATTCCTGTAGTCTTGGTTTA

>HRA5KJN01A9SMY

ACCTTTGTATAATGTACTGCCAACTATCTAAGGCATTGGCATTGTAGGGGCTGTTCGGCAGTACATTATACAAAGGT

>HRA5KJN01A8Z0R

CTGCGCGAACTAGGAGTGGCTGGTTTGTCATTCATAGGGAAACCAGCCACTCCTAGTTCGCGCAG

>HRA5KJN01BHRHS

CGTACGACGTGTCGTCGTACGGCGTCTACTACGGAAGGTACGACACAGGGATAG

>HRA5KJN01ASDSH

ACTCATGGCAGTTATTGCCATGAGT

>HRA5KJN01AJPMK

GTCATCGTAAATATTTACGATGAC

>HRA5KJN01BYTBX

CACAGTACGTGGTCGGCGTCTCTAGAGCACAGGAGGTAG

>HRA5KJN01BAD0O

GTAACGGTAGGGTTCGATCCGTCAAGTGCTTCCGAGATTCCAGTTGCTGCACCTCAACCCACACCAGTACAGCAACCAGTTCGGGTAGCACTTGACGGATCGAACCCTACCGTTAC

>HRA5KJN01AJ8ZG

CCCGAGACGGTGATCCCGCCGATGCCGAGCAGGAAGCCCGAGAGCGGCCCTACGTTCTCGGGTGCCACGCTGACGTTGCCGAGCCACGGGATCACCGTCTCGGG

>HRA5KJN01AM08X

ACACTAGTCGTCGGTCGGTCCGGTCGTCTCTAACGGACGACACCACGGGAGGTAGG

>HRA5KJN01AE797

CCAAAGCCCAGCTCAATAAGCTACTTTGGATGAAGCAGCTTATTGCGCTGGGCTTTGG

>HRA5KJN01BWUZ2

AGGCAATGTGACTGTGGCAAGCACTATGGATATGGGAATTATTCGTGACCTCTTCACCAACGTGATTGCAGCGAGTGAAATGCTCAACACAGACAAAGTGTTTAGAGAAAAATTATTGCGACGAAACAACAATTGTTTCCGTTTCAAATTGGTAGCAAAGGCCAGCTACAAGAATGGTATAAAGATTTTGACGATGTAGACCCGCAT

>HRA5KJN01APNW8

GTGTATCTTGCACACCTCCCAAAATAACAATAGGCGCGGGACTTACACATATTAATTTGTACACGCGATTTCTAGAAGCACCACACTGGTACCATCTCATTCGCCACTGGTAAACGCCCAACTGGCTAAACTCCAATAAATCGGCAGAATGAAAGCGCACAGTGCGT

>HRA5KJN01BX68T

AAGTCCTGTACTTACAGCTGTAACAGGAGTTGTGTGTATAGTATA

>HRA5KJN01AAWYJ

CCTACCGGGACCTCTACTGGAACCACGTGTTCGCCGACGATCCGCGCCGCGGCGAACACGTGGTTCCAGTAGAGGTCCCGGTAGG

>HRA5KJN01BGSF9

ATCAAGTGTACTCCCAAGTGGAACACGCAGCACTGAATCAAGCGATGGAACGAATTTACGCAATTACCGAATCCAGCCCAATGAATTAAAGCGTACAGGCAGT

>HRA5KJN01BT111

GGCTGGTGTACCCTATCTTCAATGATGGGTTCATTGAAGATAGGGTACACCAGCC

>HRA5KJN01A4RD5

CACAGTGCGTGGTCGGCGTTCAAGGCACACAGGGGATAGG

>HRA5KJN01BTE9B

ATACACTCGCTGCGCTCACCTATGCTGTCAATCTGCTGGTGAGCGCAGCGAGTGTAT

>HRA5KJN01BGJPV

ACCCGAACACAAAACAGCTTGGTGATGTGTTCGGGT

>HRA5KJN01BE1MC

ATAAACGATGGGGTAATGATAGGGAGCCTATGGGGAGCCTATCATTACCCCATCGTTTAT

>HRA5KJN01AUN0S

ATACTCTTTAGCGTGCGAGATTACATTTTTAAATAAATCTTCTGGTTTCATTTAAAAATGTAATCTCGCACGCTAAAGAGTAT

>HRA5KJN01ANXP7

GGGGAAAGACCGGCGACCGCTTCTCTGAATGTCTTCCACCTTGGGAGACCATAGCGCCCCTCCGAATCATGGGTGGGGGACATTCAGAGAAGCGGTCGCCGGTCTTTCCCC

>HRA5KJN01BMSE6

GGAAGATTGATGTGATTTTAGCTAGGTGGAAAATCACATCAATCTTCC

>HRA5KJN01BRXOL

CGTACGACGTCGTCGTCGTACGGCGACTACTACGGAAGGTACGACACAGGGATAG

>HRA5KJN01AXXZ7

GCCGGTCTTGCTGCTGTGTGCGCTGCGGACAAACCGAACTTCGCTGTGATAGGTTGCTTCGATGCTCATATCAGCGCCTCCTGCTTTGGCGCTAGATCGCTTGAGCTAGTTCCGTTGAAGCCGAACATGTCGCCCATGCGCTGCGCGTTTTCGATGCGCTCGCAGGCAATGTCGAAATACTTGGGCTCGCGCTCGATGCCGATGAACTGGCGGCCCATCTGCACAGCAGCAACCCCAGTTGTGCCAGAGCCCATGAAGGGGTCAAGGATGGTTTGGGGTGTGTTTTTGCACTGCTCAATGCACCACGACATAACTGCACAGTGCGT

>HRA5KJN01BTY0P

CCACAGTAGTGACTTCCATCTTCGTCTTGTTGGACTGAGACTTCTCGTAAAGCTTCTCGAAAGTATGGACTTCGAACAGCTTTACCATATCTTCCTCGTGGAATTTGATCGTAGTCTGGTTTGATCT

>HRA5KJN01BEUY4

CACAGTGCGTGGTCGGCGTCTCAGACGCACAGGGAGTAG

>HRA5KJN01BAEPJ

TATGGAGAAGATGTTTGGCAAGGCAATAGCCCGACAGCTAAAAGAATAAACGCACAAAGATTTCCGCTTATTGAGAAAGCGCAAAAAGGCGAACTATC

>HRA5KJN01BNWC7

GGCACCGCTGTACTAGGGTCCGATATGAAGTACTCCAGGATCAGCGGTGCC

>HRA5KJN01A1IJM

AGATGATATTTTACACAAAGATGCAGGTTGTGTAAAATATCATCT

>HRA5KJN01AOX7E

GTCCAACCGACGCTCTACTGCGCGCCGCAAGGGACGGTCGGCGACGGGGCGAAGGAGCTCCTTCGCCGGC

>HRA5KJN01A092C

CTTCAACGAGGGTAAGCTGCGCATTCTTCTCGGCAGCTTACCCTCGTTGAAG

>HRA5KJN01A2NOR

GAGGAAGTGTTGGCAAGTGTACTTCGGTTGCCAACACTTCCTC

>HRA5KJN01AGJKW

GTTGCGCTTCGCGGTGCGTCCCGTCGCGCGTGCGGTCGACCTCGCGAGCGTCGGGTTGATCGCACGCACCGCGAAGCGCAAC

>HRA5KJN01BAHL7

CTGCGTGTCTCCGACGACTACGTAG

>HRA5KJN01ACUP7

GTCGCGTCGTCGTCGTACGGTCGTCTACTAGGGACGTACGACACGAGGTAG

>HRA5KJN01ARURU

ATCAGGATGCCATCAACAAGGGCATCCTGAT

>HRA5KJN01A6SW1

ATAAGCTGAACAACGGCGAAGCGTTCGATGGTTATAAGCTTGTTGAGGGTCGCTCAAGTCGCGCATGGATAAATGAAGAAGAAACGGCGAAAGTATTATCTGAAAAGCTGTCAGCCGATGAGATGTTTAAGCATACGCTAATCAGCCCTGCCGCAGCCGAAAAACTGTTAAAGACTCAAAAGTCTATACTTGAAGGTCTAGTTATGAAATCCGAAGGCAAGCCCACGCTAGTACAATCGTGTGATAAACGCGATGCAATAAACAAAGCAAATGTGTTTGACGCACTGGATTAAAGCGTTTATTAT

>HRA5KJN01BRFVH

GCAGACGTGGGGCAAGGCCAGGGCCAACGTCTGC

>HRA5KJN01A9PDE

TGCCAATCCACAAGCCACGCAGCGCATGTTGGATTGGCA

>HRA5KJN01BV3EJ

AATGGCGTTGGCGAGCGAGGTGGCCACGCGTGCCAACGCCATT

>HRA5KJN01AK69U

AGCGATTGATCGAGATGGTCAATCGCT

>HRA5KJN01BIDJH

TGATGCGTTCCCATCGCGTGTTCTTCCGCCTCTATGATACACTCGATGGGAACGCATCA

>HRA5KJN01BND1N

CGAACCGTGTCCGCATTGAGTTCATCCCATTCGGACACGGTTCG

>HRA5KJN01AJLT8

CGTGGAATGCTTATTCTGACAATAGTGGTGGGTGTTGGTTTGCACTTAGATCTGACGCACAAGGCGCAGTGCAAGCAACTTTATCGACGATACCAACGACCTTTGCAAGCATTACAACCAACAACCCAAACCCTAGGGGTGATGCAAATTGGTTTTTAAGATCGGATCAAGTGGCCGGAACAGGCGCTACCCACACGGGTGGTAATAACATCAATTCATCG

>HRA5KJN01A3C6S

TTTGACCCGTTCTTCTATGAGCGACTTGCCAAGACTGGCGACAGCGTGAAGGGCGAAATTGTGGGCGAGTATGGCTTCGTGGTTGCGAATGGTTCT

>HRA5KJN01AGAJ6

GATCGTAGCTGATCTGTGGATTGCTGCTATACACATACCGATCTC

>HRA5KJN01BC6UM

GGCACTACCGGCTTCAGCGGCCGGTAGTGCC

>HRA5KJN01BN3M7

CGCCTTCGCGACGGCCTGAGAGTAGGTCAGATCGGCATCGGCAACTCGTTGTTGCCAACGAACTGTGGTGCCACAAAGACCTACTCTCAGGCCGTCGCGAAGGCG

>HRA5KJN01BU3H3

GTTACATTGGGCGGAGCTTGAGTAAATACTGATGGCAGCTGTTGTAGCACCGCCGTCCACTGACTAGACTTAGCGTTTTTCTCTACTCAAGGCACACAGGGGATAGG

>HRA5KJN01BEYUD

AGCAGGATGGCGCCGGGGTGCGACGCCAGGATGCCCCAGACGTAGTCCAAGGTGATCTGGACGTTGCTGGTCATCTCTTCGTCGATGTCGATGAGGTAGCCGTCGACCATCTCGGTCAGGTTCGGGTAGCCCAGAGCCAGGGCCTTGTTGTTCAGGGCCCAGGCTCTGGGCTACCCGAACCTGACCGAGATGGT

>HRA5KJN01ANWTM

GGGAACCTGCTCGAATCCTTCCTCTTTGTCAAAGGGTTCACGCTAGTGAAGTTGCGCTAACTCCACTGCGTGATGATGAATACATTGATCAAATCGTCGTTCGTTTTGCTGGGCGTGGATTAATCGTATTACGGGTAACACAGATTAACGGTGTACCTCGTTTACATATCGATGAGGTTGATGCTGCTAGTGGAAACACCTTGAAACATCGTGTTACAAATGATGTAGATTTAGTCGATCTAATGGATGAAACTCCATTTGCAAGTGGAATGCTTTCTACAGAAGGGGAAGGATTCGAGCAGGTTCCC

>HRA5KJN01AU6UN

GACAATTGAATCCATCTCGGCCTGCGTCTTACCGATCTGCAGCAAGAACACTTCAAAATCGAGGCGGGCCGAGATGGATTCAATTGTC

>HRA5KJN01A0PL6

GGAGCGACGGCTGGCCTACAGCGATCTTATAAGGTGCATCGCGCGCGACTAATCATCGCTTCGCGTGGCGATTTCCTCGTCTCGATAGTGCGGATGAATTATCGCGCGCCGGGGAAGCCTTGACGCTGTAGGCCAGCCGTCGCTCC

>HRA5KJN01AR45K

GGACACACTCAATGACCGCTACGGCTTCGAGTGTGTCC

>HRA5KJN01AC3WE

CTCCAAACAATTTATTAGAATTTTTAGGAGCTGTGCTGACACTTCCTCGAGTGGCAGATTCTTTATTTCTGCAATCTTTTTGACCACTTCTACCACATAAGCTGGCTCATTTC

>HRA5KJN01AQCXR

AGCCGTCAATTTCAGCGGTAGCAAACCAACTTTCTTCGTGGCTAGTATCCCCGTCAAAATCTTCGTGGT

>HRA5KJN01BYFR4

GTGCCAATAATCGAACATACTCCCTGCCATTGAGTGTAAACTGATTCGGCTAATGTTAGAATATTCATTTAGATGCGGTTTTGTATGCTTTACAATGTTTATAAAAGATGGCTGAAATTTCCCCATCTGTGGCCTCAATCAATCCCTGTTCTATCCTGCCCATGTCGTAGTTTAAACCTTCGCAATCCATGACTATTAGGCTGTCTTTTATCACCATGTCGGGTGCCAGGATTTGCCCATTTTTAAAATACTCAACATTTACGGGCTTTTCCTGCTCCAGTACCATTTCTTCAAGTGGGTAATATTTCGCTGGGGCTGGTTTGGGATCGT

>HRA5KJN01BI5Q8

TGACTTTGTAATAGGATTTTGCACTGGAAATGGCAAAATCCTATTACAAAGTCA

>HRA5KJN01AX55Y

GCTCTTTGATTAGCGGCAAAATCAATTTGTGGTTGTTGTACTACCAAAGGAGCATAATTTGTTGGTTCAAAAACACTTGCTACAGGTTCTCTTTGCGAATCTC

>HRA5KJN01ARH93

AGCTCTAGCAGCTCTACTTTCCTAGAGAAAGTAGAGCTGCTAGAGCT

>HRA5KJN01ALSTW

CATAGGCTCGGGCGTCGTCGATCGCCTGCGCGAGCTGCAAGCCGAGCGCGTAATACATCGAGACGTAGAAATAAGAGGCGTGCAGTTTGGCTCTGCAAACTCAGACGCGAAAGACGCAGCGCGATACGTCAACGTGAAAGCGCGCATGTATGGGCTGCTAGCGGAGGACTTAAAGAACGGGCTCCAGTTACCTCCGCCGCCTATGTC

>HRA5KJN01BQWQN

TCATGTTTGCTTTACATCCAATGGGTATTCTTACATGACCTCAGATGAATGTCTTTCCATTAGTCTGAGGTCATGTAAGAATACCCATTGGATGTAAAGCAAACATGA

>HRA5KJN01ATCG1

GAGAACAGCAGCTCGTCTGCTGTTTCTC

>HRA5KJN01BBDH2

TTCTCTTAATTTGCGTTCGGTTTCAGTTTCTTTGGTTTCTATTTTTTCCGCCTTATCATCTCTAGCAATCCAACCACTAACTGAAACCGAACGCAAATTAAGAGAA

>HRA5KJN01AI9GR

TAACTGGGATGGAATAAGTGTAGAAAATCTAATGGTATATGCGACCAAAAAAATCGTTCCGTCAAGGTCAGATTTTATTAATATATACGGCGCAATGCCATATACCCGTTACAAATACGCTGAGTGTGTAAATGGTCAGTTTTACTTTTGGGGGCGTAATAAAACCGGGATGGGCGGCAATGGAA

>HRA5KJN01BZQWI

GGCTGGCGAGCCCAAGCCGCAGCGCCTTGGTCGCTTGCATAGATAGCCTTCCTGTTCGTGCGGCCCGCTTGCTCAGCCTTCATGCGTTCGTTCTGCGCGACAGTCAGCGCCGCGACGAGCGCATCGCGCTGTGATCTGCCCGCTAGCTCGGCTTGAATGGCGTTGGTGTAGCGATACCAACCCCATGCCTGCGAGCCACCTGCGCCTATTGCGATGCCGCATAGGAGGAGCAGGACTACGCCGAGTGACCTATCCATGACTTCCCGATAAACGGTACTTGTGCGTTACTACTAACGGTGTATAGTTCACCCATCGGCAGGGCAAACAGGGGAGCGAAATGAACG

>HRA5KJN01BYWBB

CCCGTTGGCTCACACTCGCGCAACGAACAAAATCGCGAGTGTGAGCCAACGGG

>HRA5KJN01BEVNA

CGGCATCTTTTGCATTTGACATAGGTATTGCCCGGATGCGGCTTTGCAAATAGGCTTATCGCTTCCGCTTCACCCCCGCAGAACGGGCACGGCTTCAATTCCGGCGCGCTCATGCGTCACCCCCTTTCCGGGCGAGGGCGGCAGACACGGGCGGCAACCCGTGGACTTGATC

>HRA5KJN01BS6IF

CGGAGTTCCGCAACTTCGTCAGACGCGCGATCCGGCGCGTCTGACGAAGTTGCGGAACTCCG

>HRA5KJN01B26MZ

CGTACGACGTGTCGTCGTACGGGCGTCTACTACGGAAGGTACGACACAGGGGATAGG

>HRA5KJN01B0JYA

CTTTGTGCCATTGGAGCCGACGATCCTTGCCATTCGGCGAATTGACCGAGCGTGCTCAGATGGTGAAGGCCGATATCTACGAAATCA

>HRA5KJN01A9CTC

CGATCTGCTTGCCGTTCGCGATTGCGCCGCCCTTGCCTGCCGCCGTGATGCCGAACGGCAAGCAGATCG

>HRA5KJN01AQPD2

CGCTGCTCGACCACGTCGGACGTGACGGCGTAGTCGAGCAGCG

>HRA5KJN01BUIAQ

TTGAATGTTCAACTATTTTAATGTTAATATACCGCTGGAGATTAACATTAAAATAGTTGAACATTCAA

>HRA5KJN01ARSN1

CATTGATGTGTTGAAATAGCATCGGTTTGCCATTCTTTTCTTTCGCTGACTGCCAGCTCTCTAACTCCAGAAGCCCAAACTTTTTAATCGATACAAGACA

>HRA5KJN01AZN5K

GGCTAAGTTCGTAGACAAACAAGGGAACGTCTATTACCTTGTTTGTCTACGAACTTAGCC

>HRA5KJN01BFBSZ

CCGCATGAAGGCCGAGCGCGCAGCGCCGGCCTTCATGCGG

>HRA5KJN01B167D

AGGTAAGAACTTTTGCCGCCCGACCTATACGGAATACTATGACAGACTAATGATTGACATTTGCAACAAGCATGGCAAAAGTCATCGTTACCGTTGGACGTACATTGAGGCCGATAAATATTCAGGGGCTGGTCGCAGAATGAGTACGAAAGCAAAATCAGAATTAAAGATTATTAAGTCGGGGCTTATTCCG

>HRA5KJN01A6B7I

CAGTGTCTTACTGGTGCATGTAACCATCTCTTTAACTGCTGGAACTCCAGCAGTTAAAGAGATGGTTACATGCACCAGTAAGACACTG

>HRA5KJN01A35C1

GATGACACAAAATTTATCCTTCCAAATTTTGTGTCATC

>HRA5KJN01ADZQQ

CCCGTGCGCCGTAGACGATCCGCGTGAAGAAATCGCGGCCCAGATCGTCAATGTCAAACCAGAATTCGCCCGATGGGAAGGCCAAAGCTTGGGTCAGAAAATCCTGCGCCTCATAATGTGTTTTGGTGATCAGGGGCGCGATTTCTTCACGCGGATCGTCTACGGCGCACGGG

>HRA5KJN01ALJ7Q

AAGCATGGGGATCGGCGCGTTAGAAGCGGGTAACGCCGAAGTCGCCGATCCCCCATGCTT

>HRA5KJN01ABGCQ

CTTCACTTCCGCTTCGTGCGCGGCTTTAGCAGCATCGTCACGCCGTTTTTGCTCGGCATTGAGATACACAGTCAAGGCATCTCGAATGGTCTTTACGGTAGCGTCTGCGGCCTTGATGGCAGGTTTGAATTTAGCGTCGATGCTGCTAAAGCCGCGCACGAAGCGGAAGTGAAG

>HRA5KJN01ARCJP

CGTACGACGTCGTCGTCGTACGGGCGTACTACTACGGGAAAGGTACGACACAGGGGATAGG

>HRA5KJN01BYVY6

GCTCCGCTGACGCGCATGGCCAACCCCACCCGATGGAGGCAGAGGCCGCCGAACACCGCTGATTGCCGGGAGCGGCGCAGGATGCGGCGGATCTGGCATTCGGGTGGGGGTTGGCCATGCGCGTCAGCGGAGC

>HRA5KJN01BA3UI

ACGCTCTACGGCGATGGGGTATTTTCTGAAGGAATCCGGCGTGCAGCACGCGACATAACGAACAATACAGAGGAAAATACCCCATCGCCGTAGAGCGT

>HRA5KJN01AQNMW

CTCAGGTACAGGAGTCGCTTTAATACTGAAGTTCCAGTCGTTCGCAGGCAGCAGTATGTCACCCATACGCGCACTAGCAGCATCAACGAACGGTCGGGTAATATTAAGAAACTCTACGCACTGGCTATCTGACGCATTCAGCTTGGTTTGAACGAGGCCGCCAGTATCGGAACGCGGCTTGATGTACTGAACGACATTGCGATTCAGATCATCAATGCCTTCGTAGTATTCTTCATCTTCTTTCCAT

>HRA5KJN01BPHMR

GTGAGCGTGAACCCTGGTGCGTACACGGAGGGGGCGGTGTCGAGCTCGCCGAGGTGGTTGACGCGGTAGAAGCTCATGACCCCGCCCCCTCCGTGTACGCACCAGGGTTCACGCTCAC

>HRA5KJN01A6PLD

AAAGCCTTATGCCTACATCCTTGACCACGTAAACAATATCACCCGACGGACTTCCTACCGCTCCCCGCCACTGGACACTATCTTGTAAGGTAAAATCGAAGAAACCTACTGAGAAGTCACTGCGAACCTGCGAACGCTGTTTCCTCGCCCACGATCCTGCGCCCTGCTGCCCTATGTGTGGCTACGAATATCCGAAGAAGGAGAAAGCCCTGACACGTATTGAGAGCGTCGGTGGCGAGTTAGTTCGTATTGAGGAAACCAAAGAAGAACGGGCGGTGGCAATGCGCGATGCTCGGTCGATGGTGGAACTGATTGCATTTGCGAAATCACGGGGGGTATAAATCCCCGGCATTTTGGGCCAGAAAGGTTTTACGGAGGACGCAGTTACCTTGGAGCAATGCCCCTAATACCACACTAACACTATGGACAGCGAGACACTATCACTAATTGAAACCGCAAAGAAAGCAGCGGACGCAATCGGCGAGTTTGCCGATAACGTCGTCATCTTGTCTCTTCCCGAGACGCGGACGGCAACC

>HRA5KJN01BHEBZ

TTTCTCTGCCAAATTGGTTTACAATTCGTTCATCGTCTTTAAAACTATCCCACAAGGTTAATATAAGCCTTACAATAGCATCTTTCCATTGCCAATTGAAGTAATACTCATTTGCGGTGTTCTGTAATAAAACGGCTTCCATATTGCTTGCAATCACTTCATCTGTTAAAGTAGGCAATACTTCGGCTTTGTTTTCTTCCCAAATCT

>HRA5KJN01BLVGN

CACAGTGCGTGGTCGGCGTTTCCAAGGCACACAGGGGATAGG

>HRA5KJN01BOVK2

TAGCAAGCTACTCGTGACACAGCGCAATGCGTTGCGTGCCAGAGCGCCTGGATTCCTTGATAACAGCAGCACGACGGTAGTGCAGCAGCCAATTGCATTCCCCGAAAGAATGTCAGGCACCCAATGCGCAAGCGCAGAGAATCTTGCGCCATCGATGGGCGCGCTGAATCCAACCGCTGTGGCCGTGTCATTGCGTGGCCGTGAAGGCGGCGCTACGGCAGAA

>HRA5KJN01A8568

CACTAAGACCTGAAGAAATAAGAGCTAGAACCGAAGGTATTAGCTCTTATTTCTTCAGGTCTTAGTG

>HRA5KJN01BKLFJ

CACAGTGCGTGGTCGGCGTCTCTCAAGGCCACAGTGCGT

>HRA5KJN01AXKVO

ATAAACGATGGGGTAATGATAGGGAGCCTATGGGGAGCCTATCATTACCCCATCGTTTAT

>HRA5KJN01BAP7O

CGCTGGGTCGCGGCGGATGCTGCCTGCAAAGTTTACATCGGTGGTCGCTTTACGACTCCCGCCGCTGGCATCGCCGCGACCCAGCG

>HRA5KJN01A8GI1

GGCGCTGGTGTCGATTCCTGATCCAACGCTGCTCAAGCCACCGCGCGCCCGTCCCGGCAGTGCTACACCATCTGCGTCAGTCAGGGCAATATCTCCGGTCACAGTGCCGCCGCTGTAGCTGGTCCTGCCGTTGGCATCAACAGTCTTGCGAACCGCTCCGCCTGGGTTGCGCGCCAGTGTGGAGGCGTTGGAGAATTCGTCAACGGGTGCGGCTGTTGGTGTCGCGGTAGGCGCTGGAATGGCCGGATTTGT

>HRA5KJN01AN9AV

CACAGTGCGTGGTCGGCGTTCAAGGCACACAGGGGATAGG

>HRA5KJN01BML1A

ATCCATGATCGAATATTTTTGTGAGAAGATATATAAA

>HRA5KJN01BQZ31

ACCGCCGGTGCCTCCTGTCATCACCGGCGGT

>HRA5KJN01BDMRJ

ATCGCGGTGCTGGAGGAACGGCTCGCAGCGCTTGAGAACGGCGTCGGCGCGGTCTGCACCGCCAGCGGGATGGCGGCGCTGCATCTGGCGATTGCGACGCTTCTCAATGCCGGCGATCACATCGTGGCGTCCGCCTCGCTCTATGGCGGAACGATCAATCTTCTCACGCATACGCTGCCGCGCTTCGGCATCACCACGACCTTCGTCAAGCCGCGCGATCTTGACGGCATCCGCGCCGCGATCAAGCCAAACACCAAGCTCGTGATCGGCGAGACCATCGGCAATCCCGGTCTTGAAGTGCTCGACATTCCCGCTGTCGCGAAGGTCGCGCACGACGCCGGCATTCCGCTGCTGATCGACAACACATTTGCGACGCCTTACCTCTCCCGCCCGATCGAGCTTGGCNCGGACATCGTGATGAACTCGATCACCAAGTGGATCGGCGGCCACGGCATCGCC

>HRA5KJN01A6MDQ

ATTTTTGTTCCTGAATATGGTGATGGATTCAGGAACAAAAAATACGACAGTGCGT

>HRA5KJN01A3G08

CGAGCGCACCAGCGAGGCATCGGCCCGCACCCTGCGTTTCGGCTATGATGGCGACCGAGGACGCGCTGGTGCGCTCG

>HRA5KJN01AA7CB

CACTAGTGACGGTCGGTCGGCGTCGGTACTAGAGACACACGAGGGAGTAG

>HRA5KJN01BK96A

CCTTACTTTTGAAGTGGACAATCAAAAGTAAGG

>HRA5KJN01AL8BB

CTCTGCGGAAATCCTATACTGCGGGGGCGCGGTTGGCCTGAATATTCCCCCGACCAATTTCTCAACCACCAACTGCCCGGCGCTGGTGTATAGGATTTCCGCAGAG

>HRA5KJN01BF387

AGCGTCATCAAGCAAGAGCTAGAGGAGTAACCACCGGCGTCAAAGCCTACTACGCGTCGCACTGGGAGAAGACAGTCGGCTACCGCGACATCTCCAATCAATCGGGCGCAATCCAACTCTCTGGCAAAAAAGAGGGCGCATGGCAGCAATCGCGTATCTACTGCAAGCGCACGCGACGCGGCAAGGACCCCACTGTCGAAGCCGTGGGTGTGGGTGGCGCTATCACTGGTGGGCACTCTCTAGCTCTTGCTTGATGACGCT

>HRA5KJN01AT86Z

AGGTCAACGACGCCTACGTTGCACTACAAGAAGCAATGAAGCGAGAACGCATATGAAAGCCAACCCTGACCAACTCGAACGTATCGCGCTGGAGTCGTTGACCT

>HRA5KJN01B1K69

CAATTTAATTGAGATTAATTTGCAATTAATCTCAATTAAATTG

>HRA5KJN01AEVR2

TTAGCTTTGCGGCATGGATACTAGAGCGCAAGAGTGACTACCTACTGCGCTCTAGTATCCATGCCGCAAAGCTAA

>HRA5KJN01BCR0J

AGCTAAACGCTTGTAGTAGTGTGATCGTGTACACTCGGCAATACTACAAGCGTTTAGCT

>HRA5KJN01BTYSB

CCGCGAATGGATGCGCCACGACAGCGTAGGTGCTGCCGTGGCGCATCCATTCGCGG

>HRA5KJN01BIYZ1

ACCACGGGAAACTCGCATGAGCCAGTTTCAGATCCTCCGATCTTCCACCGATCCCACCATCTGCACTCGCCGCAGGTATGCGCAGCTTGCTCTGCCGGCGCTTGACGGCCAGTCCTGAACGCCGGGCAGTCCGCGCCGCCAGATGCAAGCGTTAGATTCCAGTCCGCTTTAAGGACTGTGACCGGCAGTAGCACCGGAAAGCCGCAGATCCCATAGTTGTATCCATCCCGTGGCTGACTCCACCATGCGCAGCCGCCGCACGTCTTGGCTTCGTTCATCATTCCTCCTTGTTACACT

>HRA5KJN01BQ6DH

CCGCAGTCCTGTATGCTCAGGCACATAGCCACCTTCAGGGTCTGGCACCTGAGCATACAGGACTGCGG

>HRA5KJN01BCB3Y

CGACGTACTGTCGTAGGTCGACGTACTACGGGTATAAGGGCACACAGGGGATAGG

>HRA5KJN01AJ8MM

CTCGACCCGGTCCGCCACGTTCATCGTCAACGACTGACGGTGGCGGACCGGGTCGAG

>HRA5KJN01AA84Y

TGCTCCGCCCTGCATCGCTCTTGGAGCA

>HRA5KJN01BA3IN

TCATGTTTGCTTTACATCCAATGGGTATTCTTACATGACCTCAGATGAATGTCTTTCCATTAGTCTGAGGTCATGTAAGAATACCCATTGGATGTAAAGCAAACATGA

>HRA5KJN01AHHQG

CCCGCTCCACCGGCGTGAGCTCCGCCTTGCTGCGCTGCGCGGCCTGCTCGAACATCTCGCTTTTGGCGAGCTCACGCCGGTGGAGCGGG

>HRA5KJN01BF684

ACTGATGGGCGGCGAGGGCAGCAAGTTCCTCAGGTTCCTCGGCGGACCGTGGGGCATCGCCATATCCACCGCCGCCCATCAGT

>HRA5KJN01AVPLD

ATTCTTTTTGACTGCGGCCCAGAGCAGCTCGGGGCTCCCCGAATTGCGCAGCTCGCAGTCAAAAAGAAT

>HRA5KJN01BVAYG

AGTTCATTGAAATGGAATACCGGCTGATCGTCTTCGTCCTGATGTTTCTCGGCTAATTCCATGTTGCGTAGGCACTGGCGGAAGTACGATTCCTTCAACTCACAGCCGATGCCCTTCCTGCCCATCACCACGGCCTGATACACTCCCGATCCGATGCCTAGAAACGGCTCAAGAAAACCTCGTCGCGGTTGCTCCACATTTTAATGCACC

>HRA5KJN01BX52M

CTGACGCTCTTGAGCTTGGCTTTCGGTAACGACTTGGCCAGACGACGTGGCGGCCAAAAGCTTCAGCTGTTGCTGGATCAGATCGCGCTTGGACGAAGCATTCAGATCACCGTTGGCGCCGACGGCACGTTCTTGGCCAAGTCGTTACCGAAAGCCAAGCTCAAGAGCGTCAG

>HRA5KJN01BREEM

GCCGTAATGCATGGTATCCCCGGTACTGATCGTGCGGTAGTAGATCGACTGGACGCATGGCAGGGATCACATGAGGCACAAATTGACATCAACATACCTGATGATGGTAGTACCGCTAGGAGTGTGGAAGAGTGG

>HRA5KJN01B1112

ACTTTATATGTATGAATATTATAAGTATATAAGTATATTGTATTTCTATTCTTATAATATTCATACATATAAAGT

>HRA5KJN01A3287

AAAAATTTGTACCGTCGATACGATTCGATCACGGGTCAAATTTTTACTACAGTGCGT

>HRA5KJN01AANSX

CGTACTCGTCGTACGGTCGGTACCGTACGTCGGTACTACGGACGACACACGAGGTAG

>HRA5KJN01AKKIB

TGGCGCAGTTCCTGGATCACGCGCTCTTCGGCGGCGTGGGCGTCGGAGCCGTCGGCCACGGACTGGTCGAGCGTGTCCAGGAACTGCGCCA

>HRA5KJN01AGWT5

CACGAGTGTCGTCGGTCGGTCGTCTCTAGACGACACACGGAGGTAG

>HRA5KJN01BPE2L

TCATAGCAGCACGAGTTGCAGCAACAGCGTCAATAACTCTGTCTTTTATCTCTTGAGCTTGCGTTTCATTTACGCCACCTACTTTAATCGTACCGATAGAACTTTTAAGTGCAGCGATCCGGAGTTTTAAGTGAGCTTCGTCTACTTCGTCAGCTTCAGCCTTCTGTTCAGTCAGTTGTGCAACACGAGCTTCAACGTCTTCCGGTGCAGCATAGCCACCAATAATAGAAGTTTCAGCGTCAGTTACTACAACTTTCTTAGCACGTCCGAGATATGAGTTATCTAGACT

>HRA5KJN01AU3F6

CATCCGCATGGTAGATGCGGTAACGCGGTCGCTTCAAGGCGACTGACTGCGGGCCCAGAGTTCAGTCTCTTCGGTGCGCCGAGC

>HRA5KJN01BF9HU

AGAGTCGTCCAAATACTTGAGCATTTGGACGACTCT

>HRA5KJN01AYJOD

CTGCGGTCACGCTTGACGGTCCATGTGGTCAGGCGCGATGAACCGTCAAGCGTGACCGCAG

>HRA5KJN01AFAWT

CGATTTGCTGAAGCATTGCGTGTATGCAATGCTTCAGCAAATCG

>HRA5KJN01BJ0WJ

AGAAAGGAGGATTACCGGGATATTGAACTTCTTAGCCATGGCCTTGAGTGATCGGCTGATGTTCGAGATTTCCCTGTTTGGATCGTTGCCGATTCCCTTCATGAGGCCATGGCTAAGAAGTTCAATATCCCGGTCATCCTCCTTTCT

>HRA5KJN01AXUEX

ATGCTCATCCGAGGAGGGTACTTTACGACCAGGCGATAGCCGTCGTGGTCGGTACTACTTATGGTAAGGTGAGAAGCCTTGAGGATTACCGAGGCAAGAAGATAAAGGAAGCAACTCCAGCAATGCCAGTGGTGGTTTCGGGTGTTCGTGAAGTACCCTCCTGGATGAGCAT

>HRA5KJN01A5KTM

AGCCGCACCATGGCACGCTGTCTGAGCCGTAAGCGATGCCGAGCCATGGTGCGGCT

>HRA5KJN01A0ZQG

GACGTGACCAAAAAGGTCGTTCGAATTTGGGGAATGTAACGTCTCAAGCGGACTACAGGGGATAGG

>HRA5KJN01BQQIU

CCAGGTGAAGCGAGGGCTTAATTCGGCCTACAAGGGCGGAGAACCTCGCTTCACCTGG

>HRA5KJN01BFHUY

CTTCACGCCAGAATCTGTGCAGAAGTTCGCACAGATTCTGGCGTGAAG

>HRA5KJN01B2TTM

ATTGCCGCGAATGCGATCACGGCAGCATCCGTGGCAATGGCTGCTGCCGTGATCGCATTCGCGGCAAT

>HRA5KJN01BJI9Q

AAGCAGAAAGACCACTACCATATCCACTATCCACACCGTTCCCAGCCATGATCGCAC

>HRA5KJN01BKKI7

GTTTCTCCATGGGCTAGTAAAGCTACAGAGTATCGCTCGTCAGTGCGGCCTTCAGATTTTACGTATTGAACGTGGTGTTCGATATGCATGGCAAAGTAAAAAATCACTCAACGCGGAACAAGAACGGCTGATATCTGTAGCTTTACTAGCCCATGGAGAAAC

>HRA5KJN01ANG1O

TATAAATCATGGGCAAGCCAGAAATCGGCTTCGGTGTTTATGTCGATTGAACGTTCAGTTGGCATGTAAAACACGCGGCTACCGAAATACCGATTTCTGGCTTGCCCATGATTTATA

>HRA5KJN01A8Z7C

CGTACGACGTGTCGTCGGTACGGGCCGTCTACTACGGGAAGGTACGACACAGGGGATAGG

>HRA5KJN01AYMNA

ATTCCCGTTCGCCTCTCTCAAGGATGGAGCCGCCCGTGCGAACTCTGAAAACTCTCGCGTATCTCGCCGTCGTCGCCTCGTCATTGCTGGTCGTGCAATCCGC

>HRA5KJN01BKXGL

GCTTTTGACATTCCGTAAGGTGCTACATCTACTAATAGGATAGGAGATGTAGCACCTTACGGAATGTCAAAAGC

>HRA5KJN01BJ2FS

CACAGTGCGTGGTCGGCGTTCAAGGCACACAGGGGATAGG

>HRA5KJN01BOPHK

AGGCAGAGGGTGGAAATCATTCGGTCCTGGAAGTTGACAACAACCTTCAATTTCCAGGACCGAATGATTTCCACCCTCTGCCT

>HRA5KJN01AUHQW

ATCGGGGCGGACACCACTTATCCACAGAACCTGTGGATAAGTGGTGTCCGCCCCGAT

>HRA5KJN01A4YK6

CCAACGGCATCCCGCTGCTGGGCGCCCTGGGCAGCGGGATGCCGTTGG

>HRA5KJN01A4YTF

TCGATTTCCACGAGTGGTGCGGCCCCAGTTTCTACACAGATGCGAACATGACGAAGGTCTATGACCCGGTGGACGAGCAAG

>HRA5KJN01AKKQ2

TGGGGCTTTCTCTTGGTCGCTCATATGTGGTTCTCCGTGGGGATAGTGTACCAATTTGTCCACTGTTTCAGTACACTATCCCAAGTAAGGTTTACGAGAAG

>HRA5KJN01AO5FB

GGGGATATAACGCCGAATATATTCGGCGTTATATCCCC

>HRA5KJN01AM8EQ

ATCGCATCCGAACCGACGGTAAATCGGTTCGGATGCGAT

>HRA5KJN01AXWIV

GGGGTTTTTATTGGACGCTGGATAACGCCCAATAAAAACCCC

>HRA5KJN01ACUNS

CAAGCGCGGCGCATCTTCTCCTGCAGCACGAGTAGATCGTCTTCCCGGCAATGCGTGTGCGGCCGGCGTCTGTGAATGGATCAACCAGCAGCGCCCGACCGGTGCGAGCCGCGCGAATGCCTGCCGGTATCGCCCCACCCATGCCGGCGCCGAGTGCCG

>HRA5KJN01AGR8Q

CTGTATATGCCAATTTCATCGAACACGAAAGTACGTTCAGCTCCGCTTATTTCTACACCGAAGTCATTTATGGTTGCAAACTGACCAGTTGGTTCATTTTTATTGATGAACGTAGTTATAATTATATTTGATTTTGTTCCAACTTCCTGCGAAACCACACCAGCACCTAGCGGGTCATCTTCGGCGACGGAACCACCACCGGTACGAATGTTTCCGGGTTCAGACGAACCGGGG

>HRA5KJN01ARR3O

TATGCCATATATGGCGCGCGGCTGGCTCTATCGAAAATGTTCGACATGAACTGGAATCACGAGCGGGTTAGAGATTATGATTGGTTTGAGCCAATCGCCAAAGCCGAAGTCTC

>HRA5KJN01AGF5Z

CCGCATGAAGGCCGAGCGCGCAGCGCCGGCCTTCATGCGG

>HRA5KJN01A74A0

CACAGTGCGTGGTCGGCGTTCCAAGGCACACAGGGGATAGG

>HRA5KJN01AXN0O

AGCTCTAGCAGCTCTACTTTCCTAGAGAAAGTAGAGCTGCTAGAGCT

>HRA5KJN01A55N7

GCCGACGGTGACGTATGCCTTCTTGCGGCAGTCAATGACCACGGCGACGTTTGTGGCCGCACTTGCTGCCAGTGTGGCGCTGCTGTTGAGGGATTGCGCAGAGTAAACGGGGCTTTGTGCCTGGGAGGTTTCCGCGACGAATAGAACCGCCGCGCCGATGAGTGTTAAGAATTTTTTCATGGTCTTATTTTCCTTTGGATTTC

>HRA5KJN01AVNER

GTTCGCCGGTACGCACGCGCTGGAAGCGATAGCGTACCGGCGAAC

>HRA5KJN01BYACA

AGGATGCGTCTTCGCCGATCGCCGTCGCCATGGCGAAGACGCATCCT

>HRA5KJN01BPU1V

AACGTACCCTCCATCGTCACCACCACCGGCAGTTTGTGGGGCGCGAGCATTGGACTGGTCATACCCGCCCTGCTCACTTGGCCCAGCGAATCGCCAACACTGCATGCTTGGGGCGCACTCGGCATTGCAGGCTTGCTTTGTACCGCCTTGGCCTATGTGTTGTATTTCCGGCTGATGACGCGCACGGGCCCCGCTCGCGCCATGACCGTGACGATGGAGGGTACGTT

>HRA5KJN01A1BQQ

GGATGTACCACTTGCGAAAATTTCTGCAAGTGGTACATCC

>HRA5KJN01AR676

CCACTACAACCCGGAGGTACAGCCATCCCGTGTTCCTATGCCACCCGTCATCCCTGAACAGCCTTGGATGTACCCTACGGGTTGTAGTGG

>HRA5KJN01A8AUA

TATCTCGGGGGACGCTTGAATGCGCGTGGGATTGAATTTTCCGACACACAACAAACAAAGACCTTGCTTGAAACTATTCAAGCCCCTCTCCGTCCTCAGG

>HRA5KJN01ACTYV

CACTGTCGAGGTCGCGTACTCGGTACTAGGANCACAGGGAGGTA

>HRA5KJN01A1FBM

GGCGTTAATGTAGTGGAAGAAGTACACCAAGTCTTGAATCAGGTCAAGCAGTTTACAGATGGTGTTCGAAGTGGTGAGCTTCTTCCACTACATTAACGCC

>HRA5KJN01AFZW3

ATTAATCGTATCTGTATTTAATGTATTATTACTGGTACGATTAAATACAGATACGATTAAT

>HRA5KJN01BU7P1

CCAAGCCCAAGTGGCATTGATATTCCTAAGAATCTCAATGCCACTTGGGCTTGG

>HRA5KJN01BBYEU

AAACGGCCCTCTCAATCTCCCTTGCTTGTGGTGGAATTTTTCCTCCTCCTATTGCGCTTAGATCGATTGTAGCAGATTTGTGAACGTTTTTTTTGTAGTTTGTTTGTTCCATTATTTATCGTCTTTTTGATCCCACTTAATGAGCGTTAAATACGCCGATACGTTTTTACTTAACAGGGGCTTCCAGTTGTGCATAGCGACCAATAGAGTACGTATTTTATCCACGTTGTACCCATTTTTAGCAAGGCTTAAATATTGCTCAATCGTAAACGGTTCTTTCATCTGTTCAACTCTATTTC

>HRA5KJN01BKH6B

GCTCGGCAATCCACTCGTGTACCTCCGGGTAGGCGGTGAAGTCCACCATCTCCACGGCTCGCTTCTGCTTTGGCTTGCGCCGCTTCTTCTCGTCGGCCACCTCAGGCCACCGCATCCACACAGTGC

>HRA5KJN01A738J

CTACGACGTGTCGTCGTACGGCGTCTACTACGGAAGGTACGACACAGGGATAG

>HRA5KJN01BV9JA

AGGCACCCCTTTGGCGTGCTTGCCCGTCGCTGGAACCCCAAGGCATCGTTCTCTTGTCTGCTCTCCGTATTCCGGGTGCAAGATGTGGCGTCGTTTCGTCCCGCAATATCTTGAGCAAGTCAGCCCGGTCTGAGTTCCCTGAGAGATCGCCCAAGCAAGCAAGCTCTTCCGCTTGCTGTACTCCGTGCTGCACAGCCCGCAGCGAAGCCGCAAGGAGAACCGATCTTCCCGCACAATCTCGGGCAGCAATCCAAAGGGGTGCCT

>HRA5KJN01BM67K

CTCCAAACAATTTATTAGAATTTTTTAGGAGCTGTGCTGACACTTCCTCGAGTGGCAGATTCTTTATTTCTGCAATCTTTTTGACCACTTCTACCACATAAGCTGGCTCATTTC

>HRA5KJN01BU9IG

AGATCAGCTGTGCATGGTGAGGACGGGCTCGAGCACGAGCCCGAAGCTGCGCAGCATGCCGAGCAGCTGATCT

>HRA5KJN01BX9S6

GCATTGATCGGCAGCACTGGCACTGCCGATCAATGC

>HRA5KJN01AFAJL

CAACAGCCCCACCTATGTCAACCGGCACTTTGTCGGTTGACATAGGTGGGGCTGTTG

>HRA5KJN01AOB1B

GACAAGGTGAGCGGATCGGCGGTCACCTTGTC

>HRA5KJN01AZKSV

AATCTTGTCAGGCAGGCTCCCCCTGCCAATCTGCCGTACGAAAGACTCAACGCTTTATAAGCAAATTGGAACTCTTGACGATAGCCTTGGGTCGTACCCTCTACACGTCCGTCACTTACCATTTCCCCTTGGCTCTGCTAGCACCGTTACTTTCCGAAAAATCGGTACAGATTAACATC

>HRA5KJN01A5N35

CATTATATGTTCTCGTGGGCATGCTACGACGTGACCACGAGAACATATAATG

>HRA5KJN01AVDXG

GTGTATCTTGCACACCTCCCAAAATAACAATAGGCGCGGGACTTACACATATTAATTTGTACACGCGATTTCTAGAAGCACCACACTGGTACCATCTCATTCGCCACTGGTAAACGCCCAACTGGCTAAACTCCAATAAATCGGCAGAATGAAAGCGCACAGTGCGT

>HRA5KJN01A73N7

TGGGAAGCGCAGTTCGGCGACTTCGTGAACGGCGCTTCCCAACACGAGTGCGT

>HRA5KJN01AYVU0

GTTTCGACGTGGCAAACGGCGGGCGTTATGACCGGATGGAACTGCCCGAAAGAACTGCTGGCCAAGCTTGCCAGCCTTGGTGCGAGCTACGCGGTCACCCTCTATGCGGCCGCCGTTTGCCACGTCGAAAC

>HRA5KJN01AW5AP

GGAAACATTTGATACCTACTATCAACGGATTTTAGATGTCATTTGTAAGCACATGGGATTTACCGAAAAACAAATACAGGAAAATCCGTTGATAGTAGGTATCAAATGTTTCC

>HRA5KJN01AXA7S

GGCCATCGAAGCCATGCGCCCGCGCTGGCGCGAGGAGACGCGCGGCTCGCATGGCTTCGATGGCC

>HRA5KJN01BLC9O

GCAGACGTGGGCAAGGCCAGGGCCAACGTCTGC

>HRA5KJN01BS5MF

CGTCCAGCATCGACCGGTACGCACCCTGCGTGTAATTCGACTGGTTATATCGGATGTACTT

>HRA5KJN01AW5C6

GTATATGAGGCGCAATTAACCAAAGTACCTCATTATTTCAGGGGACACATTATACCTACAAAGGATGAGATAATTAATTTTTTAAAACAGTTAAAACTTAATAATATGCAAAAGAATGAAATGATAAGAGAAATTATAATGGCTGTAAACGCTCAAGCGGACAGGACAAAGGGAACACCGTTCCAAACTAAACAAGCGGACGGCGAT

>HRA5KJN01AIGAL

ACACGAGTGTCGTCGGTCGGTCGTCTCTAGACGACACACGGAGGTAG

>HRA5KJN01BTFTP

GCCGCCGACGTGCCCGCCACGCTCGGCGGC

>HRA5KJN01BZEPO

GCGGTCGGACGCGATCAACGCGCCGCTGCTCGCGGCCGCG

>HRA5KJN01AWULF

GATTCATTAGGCTCTGATATATTGTAAAAAGATATATATA

>HRA5KJN01AXNGR

GAACGCAAAAATGTTGGTCCTGGCGGGATCCATGCTGCCCGCCAGGACAAACATTTTTGCGTTC

>HRA5KJN01BEF0P

ACAAAGTGGGTGTTGATGAACCCACTTTGT

>HRA5KJN01B14IS

GACCACGCCGCCAGCATCGCCGCCCGACTGATCGCACTCGGCGGCGTGGTC

>HRA5KJN01BN6XE

ACCACGCGACCGCGGCGGGGCTCTCATCGTCGCGCGCCCGCGCCTCCGCTTCCGCGGGCGACGCAACGTGCCGCGCGAACCAGCCGTGCATGTCGTGGGCTTTGGCGAGGT

>HRA5KJN01BOBZT

ATGGGCTTGGCCATCAGGCCATCGAGCTGGGTGGCCAAGCCCAT

>HRA5KJN01AY897

GTGCTGTCACAAAAATCCGCATTAATATCGAAACTTCGCAATCAAATAATCTTTGGCCAAGCGTTCGCGTTGACCAGTGGATGCAAGGTTCACGTTTGAGAGCGACTCCAATGCCTCCGTTGGAATGGATTTACTCACCCGCCAAACTTCACCATCTAGGAAACAAATCACAAGTTCATTCCCCAAAGTCCGTGATTTAATGACATCCCAACTCCTGCCGTTTTGACCGGAGCCCGCCCCAATGTCGCCCGATATTGACCAGTTCGCCCAACCATCCGGTACGAATACAAGCACAAGCAATTCCCCAGAATATTCACTAAGCTTTAGCCCAG

>HRA5KJN01BUEFA

ACCTTGTCAGCGCGGCCTGATGTGGGAACAGCGAATCAGGCCGCGCTGACAAGGT

>HRA5KJN01BYIY3

CTACGACGTGTCGTCGGTACGGCGTCTACTACGGAAGGTACGACACAGGGATAGG

>HRA5KJN01BB5C3

CCCAAGTTTGCGACGCGCCTTCTTCTCCTCTTTCGTCGACTCATTCAGGAGCAACTAGGCGAACCTTGCGCGATGCTCTTGGCGAGCGAAGACGAGTCGACGAAAGAGGAGAAGAAGGCGCGTCGCAAACTTGGG

>HRA5KJN01AS5UW

GCTGCTGTCTGCCCTGACAATGTTGTAAACACTCTACGCACCCTAGCTACATAGCCATTAAACGCCCCGTGACCAACGCCATAGCCGATAGTCAACCTACCATTCG

>HRA5KJN01BKOIL

GGTAAGGGAACAGAGTGGACGTGTCTTTGGTGCTTTTGTAACCGAAACTCTTCATATTCAAGGTGGGTACTATGGAAAC

>HRA5KJN01BDKD4

GGCAGCAGGCGTTGCTTCCTCCCGCGGCAAGCGAGTTCTCGTCTTGGAACGCAATGTAAAGATGGGAGGAAGCAACGCCTGCTGCC

>HRA5KJN01BXAQV

GCGTGAACCGCGCGTGCTGTTGCTCGATGAACCGTTCGCTGCGGTTGATCAGATGAGTCGCGAACGGCTCTATGAAGAACTCGCGCTGTTGCGCGCGGGTTTGAATATTCCGGTCGTGCTGGTCACACATTCTCTGCACGAGGCGCACCTGCTCTCCGATCGTATGGTGGTGCTGGCGGCGGGTGCCAGTCTGCAGGCGGGCAC

>HRA5KJN01ADKNO

CGCCAACCGTGTCATATCGTATAATTGGAGATGACGGCTCGCCGATCATCGGAGACGATATGACACGGTTGGCG

>HRA5KJN01ALPAI

GAGTTGTTCTGCCAGGAGTATCTGGTCGACCTCAACGCAACGCAGGCCGCGACACGTGCAGGCTATTCAGCGCGTCAGGCTGGGGTAATCGGATACTCCTGGCAGAACAACTC

>HRA5KJN01BZRDX

CAAAGGCAGAAGACGGGCATTTACCAGCCAATCCACAGCGGCGCCAGCTGCATTGCTAAGGCTTACTACAACCGGACCAATGGCAACGTTGGCGATCAGGATAAGGATGACAGCCAAAATACCAGCCGAGAAGGTGTTGTAGAGCATTTCAAAACCAACGGGAATTTTTTCTTCCAAGGCTTCATCAATTTTCTTCATGCACCAACCA

>HRA5KJN01BVZY1

GGCCAGTGCATTAAGTGCAAACAAACCACCGGCTTCGCCTGCTGTCTGCATTGCTACTTTTTTGAAATCTATTTTAGGTAATCCTTTCATAAAAAATTGTATGGGTTTAGTAAGATTGTTTTGTGAGTTAC

>HRA5KJN01AEO4M

CACAGTGCGTGGTCGGCGTCTCTAGACGCACAGGGAGTAG

>HRA5KJN01A4XF3

CACAGTGCGTCGTCGTCGTACGTCTACAACGACGGACTACGAGGGATAG

>HRA5KJN01A03GQ

GGAAACGATCACCGATCTCGTGTACCGAGAACGGTGATCGTTTCC

>HRA5KJN01BMXAD

ATCGCCACCATTAGTTACTGGGTTTAAAGCGCCTAGCGCAGCACCTTGCATAGCGCCGCGCACAACACCAGCACCTCCCCCGATAGGTGCAGTCATGGCTACATTTCCAGCCATACGAGCAGCATCAAATCCATCACGACCCGCTGAAGTTCTAGCGACACAGTGCGT

**SPECIMEN 2 NON-DESTRUCTIVE PREPARATION**

>HYQZ1MD01DHTYJ

GTGTGTCGTGGTCGTCGTCTCTCACGCACACAGGAGAG

>HYQZ1MD01C6GKO

GTGTGCGTGGTCGTCGTCTCTCAGCACACAGGAGAG

>HYQZ1MD01DO0U1

GTGTGTCGTGGTCGTCGTCTCTCTACGCACACAGGAGAG

>HYQZ1MD01AT2K6

GTGTGTCGTGGTCGTCGTCTCTCACGCACACAGGAGAG

>HYQZ1MD01BZH9I

GTGTGTCGTGGTCGTCGTCTCTCACGCACACAGGAGAG

>HYQZ1MD01C4UJH

GTGTGTCGTGGTCGTCGTCTCTCACGCACACAGGAGAG

>HYQZ1MD01CSC62

GTGTGTCGTGGTCGTCGTCTCTCACGCACACAGGAGAG

>HYQZ1MD01AWX3J

GTGTGTCGTGGTCGTCGTCTCTCACGCACACAGGAGAG

>HYQZ1MD01A5Y7P

GTGTGCGTGGTCGGTCGTCTCTCAACGGCACACAGGGGTAAA

>HYQZ1MD01AXL93

GTGTGTGCGTGGTCGGCGTCTCTCAAGCACACAGGGAAAGG

>HYQZ1MD01BZD32

GTGTGTGCGTGTCGCGTCTCTCAGCACACAGGAGAG

>HYQZ1MD01AYRK4

GTGTGTGCGTGGTCGGCGTCTCTCAAGCACACAGGGAAAGG

>HYQZ1MD01AB109

GTGTGCGTGGTCGGTCGTCTCTCAACGGCACACAGGGAAGG

>HYQZ1MD01AP4US

GTGTGCGTGGTCGGTCGTCTCTCAACGGCACACAGGGAAGG

>HYQZ1MD01B4IAT

GTGTGTGCGTGTCGGCGTCTCTCAAGGCACACAGGGAAAGG

>HYQZ1MD01DTAK7

GTGTGTGCGTGTCGCGTCTCTCAAGCACACAGGGG

>HYQZ1MD01C7GXW

TGTGTGCGTGGTCGGCGTCTCTCAAGGCACACAGGGAAGG

>HYQZ1MD01CBE37

GTGTGTGCGTGTCGCGTCTCTCAAGGCACACAGGGGAAAG

>HYQZ1MD01AM1NG

GTGTGTGCGTGTCGCGTCTCTCAAGCACACAGGGGAAAG

>HYQZ1MD01DFJDD

GTGTGCGTGGTCGGCGTCTCTCAAGGCACACAGGGGAAAGG

>HYQZ1MD01DU37D

GTGTGTGCGTGGTCGGCGTCTCTCAAGCACACAGGGAAGG

>HYQZ1MD01AJJQW

GTGTGCGTGGTCGGCGTCTCTCAAGGCACACAGGGGAAAGG

>HYQZ1MD01CQ9YG

GTGTGCGTGGTCGGCGTCTCTCAAGGCACACAGGGGAAAGG

>HYQZ1MD01BP3N7

GTGTGCGTGGTTGGCGTCTCTCAAGGCACACAGGGGAAAGG

>HYQZ1MD01CMUSX

GTGTGCGTGGTCGGCGTCTCTCAAGGCACACAGGGGAAAGG

>HYQZ1MD01DPZAZ

GTGTGTGCGTGGTCGGCGTCTCTCAAGCACACAGGG

>HYQZ1MD01B0V3G

GTGTGTGCGTGGTCGCGTCTCTCAAGGCACACAGGGGAAA

>HYQZ1MD01B37I3

GTGTGTGCGTGGTCGGCGTCTCTCAAGCACACAGGG

>HYQZ1MD01A8F8H

GTGTGCGTGGTCGGCGTCTCTCAAGGCACACAGGGGAAAGG

>HYQZ1MD01DFQ4A

GTGTGTGCGTGTCGGCGTCTCTCAAGCACACAGGGAAAGG

>HYQZ1MD01BOZCW

GTGTGTGCGTGTCGCGTCTCTCAAGCACACAGGG

>HYQZ1MD01BC1AB

GTGTGCGTGGTCGGCGTCTCTCAAGGCACACAGGGGAAAGG

>HYQZ1MD01DPSEY

GTGTGTGCGTGGTCGGCGTCTCTCAAGCACACAGGG

>HYQZ1MD01B7LWU

GTGTGCGTGGTCGGCGTCTCTCAAGGCACACAGGGGAAAGG

>HYQZ1MD01AIM7B

GTGTGTGCGTGTCGCGTCTCTCAAGGCACACAGGGGAAAGG

>HYQZ1MD01AM3ED

GTGTGTGCGTGGTCGGCGTCTCTCAAGCACACAGGGAAAGG

>HYQZ1MD01AL5LB

GTGTGCGTGGTCGGCGTCTCTCAAGGCACACAGGGGAAAGG

>HYQZ1MD01C8AF5

GTGTGCGTGGTCGGCGTCTCTCAGGCACACAGGGGAAAGG

>HYQZ1MD01DOT7K

GTGTGCGTGGTCGGCGTCTCTCAAGGCACACAGGGGAAA

>HYQZ1MD01DQ11B

GTGTGCGTGGTCGGCGTCTCTCAAGGCACACAGGGGGAAAAGG

>HYQZ1MD01CWBOD

GTGTGCGTGGTCGGTCGTCTCTCAACGGCACACAGGGAAGG

>HYQZ1MD01BQVJD

TGTGTGCGTGGTCGGCGTCTCTCAAGGCACACAGGGAAGG

>HYQZ1MD01DWLT9

GTGTGCGTGGTCGGCGTCTCTCAAGGCACACAGGGGAAAGG

>HYQZ1MD01CTE21

GTGTGCGTGGTCGGCGTCTCTCAAGGCACACAGGGGAAAGG

>HYQZ1MD01BCKXU

GTGTGCGTGGTCGGCGTCTCTCAAGGCACACAGGGGAAA

>HYQZ1MD01DEHEO

GTGTGTGCGTGTCGCGTCTCTCAAGCACACAGGGG

>HYQZ1MD01CWTL8

GTGTGCGTGGTCGGCGTCTCTCAAGGCACACAGGGGAAAGG

>HYQZ1MD01A2W7G

GTGTGCGTGGTGGCGTCTCTCAAGGCACACAGGGGAAAGG

>HYQZ1MD01C4CCD

GTGTGCGTGGTCGGCGTCTCTCAAGGCACACAGGGGAAAGG

>HYQZ1MD01CLEAP

GTGTGTGCGTGGTCGGCGTCTCTCAAGGCACACAGGGG

>HYQZ1MD01DIKCN

GTGTGCGTGGTCGGCGTCTCTCAAGGCACACAGGGGAAAGG

>HYQZ1MD01CWZSP

GTGTGTGCGTGTCGCGTCTCTCAAGGCACACAGGGG

>HYQZ1MD01BK1WG

GTGTGCGTGGTCGCGTCTCTCAAGGCACACAGGGAAG

>HYQZ1MD01BE53B

GTGTGCGTGGTCGGTCGTCTCTCAACGGCACACAGGGGGTAAAA

**SPECIMEN 2 DESTRUCTIVE PREPARATION**

>HYQZ1MD01ASM42

CACAGTGCGTGTCGCGTCTCTCAGCACACAGGAGAG

>HYQZ1MD01B3IKI

CACAGTGCGTGTCGCGTCTCTCAAGCACACAGGAGTAG

>HYQZ1MD01C6RRK

CACAGTGCGTGTCGCGTCTCTCAGCACACAGGAGAG

>HYQZ1MD01BU6ED

CACAGTGCGTGTCGCGTCTCTCAGCACACAGGAGAG

>HYQZ1MD01CGX3X

CACAGTGCGTGTCGCGTCTCTCAGCACACAGGAGTAG

>HYQZ1MD01B60ZI

CACAGTGCGTGTCGTGTCTCTCAGCACACAGGAGTAG

>HYQZ1MD01BNJDZ

CACAGTGCGTGTCGCGTCTCTCAGCACACAGGAGAG

>HYQZ1MD01CZXE6

CACAGTGCGTGTCGCGTCTCTCAGCACACAGGAGAG

>HYQZ1MD01CJIEZ

CACAGTGCGTGTCGCGTCTCTCAGCACACAGGAGAG

>HYQZ1MD01B17KT

CACAGTGCGTGTCGCGTCTCTCAGCACACAGGAGAG

>HYQZ1MD01B6L5O

CACAGTGCGTGTCGCGTCTCTCAGCACACAGGAGAG

>HYQZ1MD01B9K7T

CACAGTGCGTGTCGCGTCTCTCAGCACACAGGAGAG

>HYQZ1MD01DAPQ5

CACAGTGCGTGTCGCGTCTCTCAGCACACAGGAGAG

>HYQZ1MD01AQ8DS

CACAGTGCGTGTCGCGTCTCTCAGCACACAGGAGAG

>HYQZ1MD01CFWYP

CACAGTGCGTGTCGCGTCTCTCAGCACACAGGAGTAG

>HYQZ1MD01CMD5F

CACAGTGCGTGTCGCGTCTCTCAGCACACAGGAGAG

>HYQZ1MD01BOB9Z

CACAGTGCGTGTCGCGTCTCTCAGCACACAGGAGAG

>HYQZ1MD01AMLSD

CACAGTGCGTGTCGCGTCTCTCAGCACACAGGAGAG

>HYQZ1MD01CVRJA

CACAGTGCGTGTCGCGTCTCTCAGCACACAGGAGAG

>HYQZ1MD01A1BX4

CACAGTGCGTGTCGCGTCTCTCAGCACACAGGAGAG

>HYQZ1MD01A857N

ACACAGTGCGTGTCGCGTCTCTCAGCACACAGGAGTAG

>HYQZ1MD01B0902

CACAGTGCGTGTCGCGTCTCTCAGCACACAGGAGAG

>HYQZ1MD01BQ1WY

CACAGTGCGTGTCGCGTCTCTCAGCACACAGGAGTAG

>HYQZ1MD01CYCWX

CACAGTGCGTGTCGCGTCTCTCAGCACACAGGAGAG

>HYQZ1MD01DAE4L

CACAGTGCGTGTCGCGTCTCTCAGCACACAGGAGAG

>HYQZ1MD01BZDJ5

CACAGTGCGTGTCGCGTCTCTCAGCACACAGGAGAG

>HYQZ1MD01CSEWE

CAGTGTCGTGGTCGTCGTCTCTCTACGCACACAGGAGAG

>HYQZ1MD01BWQ9O

CACAGTGCGTGTCGCGTCTCTCAGCACACAGGAGAG

>HYQZ1MD01BGL75

ACACAGTGCGTGTCGCGTCTCTCAGCACACAGGAGAG

>HYQZ1MD01A0WRG

CACAGTGCGTGTCGCGTCTCTCAGCACACAGGAGAG

>HYQZ1MD01CCQ30

CAGTGCGTGGTCGTCGTCTCTCACGCACACAGGAGAG

>HYQZ1MD01DM6U3

CACAGTGCGTGTCGCGTCTCTCAGCACACAGGAGAG

>HYQZ1MD01B5KM8

CACAGTGCGTGTCGCGTCTCTCAGCACACAGGAGAG

>HYQZ1MD01DKGO4

CACAGTGCGTGTCGCGTCTCTCAGCACACAGGAGTAG

>HYQZ1MD01A06F1

CACAGTGCGTGTCGCGTCTCTCAGCACACAGGAGAG

>HYQZ1MD01CWUTV

CACAGTGCGTGTCGCGTCTCTCAGCACACAGGAGAG

>HYQZ1MD01B52UU

CACAGTGCGTGTCGCGTCTCTCAGCACACAGGAGAG

>HYQZ1MD01DSWWN

CACAGTGCGTGTCGCGTCTCTCAGCACACAGGAGAG

>HYQZ1MD01CLTBY

CACAGTGCGTGTCGCGTCTCTCAGCACACAGGAGAG

>HYQZ1MD01CV6UM

CACAGTGCGTGTCGCGTCTCTCAGCACACAGGAGAG

>HYQZ1MD01DQCLX

ACACAGTGCGTGTCGCGTCTCTCAGCACACAGGAGAG

>HYQZ1MD01B4UK5

CACAGTGCGTGTCGCGTCTCTCAGCACACAGGAGAG

>HYQZ1MD01CI9OK

CACAGTGCGTGTCGCGTCTCTCAGCACACAGGAGTAG

>HYQZ1MD01A9XNN

CACAGTGCGTGTCGCGTCTCTCAGCACACAGGAGTAG

>HYQZ1MD01AIL08

CACAGTGCGTGGTCGGCGTCTCTCAGCACACAGGGAAGAG

>HYQZ1MD01CNMGF

CACAGTGCGTGTCGCGTCTCTCAGCACACAGGAGAG

>HYQZ1MD01BV1XM

CACAGTGCGTGTCGCGTCTCTCAGCACACAGGAGAG

>HYQZ1MD01CQO6G

CACAGTGCGTGTCGCGTCTCTCAGCACACAGGAGAG

>HYQZ1MD01CTDG5

CACAGTGCGTGTCGCGTCTCTCAGCACACAGGAGAG

>HYQZ1MD01AD3O9

CACAGTGCGTGTCGCGTCTCTCAGCACACAGGAGAG

>HYQZ1MD01B40XT

CACAGTGCGTGTCGCGTCTCTCAGCACACAGGAGAG

>HYQZ1MD01APKCP

CACAGTGCGTGTCGCGTCTCTCAGCACACAGGAGAG

>HYQZ1MD01B20RY

CAGTGCGTGGTCGGGTCTCTCAACGGCACACAGGGAAGGAG

>HYQZ1MD01C94BW

CACAGTGCGTGGTCGGCGTCTCTCAAGCACACAGGGAAGG

>HYQZ1MD01BFNKL

CACAGTGCGTGTCGCGTCTCTCAGCACACAGGAGAG

>HYQZ1MD01DN51O

CACAGTGCGTGTCGCGTCTCTCAGCACACAGGAGAG

>HYQZ1MD01DKGSM

CAGTGCGTGGTCGTCGTCTCTCACGCACACAGGAGAG

>HYQZ1MD01BXJ5H

CACAGTGCGTGTCGGCGTCTCTCAAGCACACAGGGAAGG

>HYQZ1MD01AA1JH

CACAGTGCGTGGTCGGCGTCTCTCAAGGCACACAGGGGAAAGG

>HYQZ1MD01AY38D

CACAGTGCGTGGTCGGCGTCTCTCAAGCACACAGGGAAGG

>HYQZ1MD01BOX23

CACAGTGCGTGTCGCGTCTCTCAGCACACAGGAGAG

>HYQZ1MD01C9ZD5

ACAGTGCGTGGTCGGCGTCTCTCAAGGCACACAGGGG

>HYQZ1MD01CPKVW

CACAGTGCGTGTCGCGTCTCTCAGCACACAGGAGAG

>HYQZ1MD01C0SRW

CACAGTGCGTGGTCGGCGTCTCTCAAGCACACAGGGAAAGG

>HYQZ1MD01C1LNV

CACAGTGCGTGTCGCGTCTCTCAGCACACAGGAGAG

>HYQZ1MD01ALT9Z

CACAGTGCGTGGTCGGCGTCTCTCAAGCACACAGGG

>HYQZ1MD01CCWQD

CACAGTGCGTGGTCGGCGTCTCTCAAGCACACAGGGAAAGG

>HYQZ1MD01BMQ67

CACAGTGCGTGTCGCGTCTCTCAGCACACAGGAGAG

>HYQZ1MD01AP9AT

CACAGTGCGTGTCGGCGTCTCTCAAGCACACAGGGAAGG

>HYQZ1MD01A8LS6

CACAGTGCGTGGTCGGCGTCTCTCAAGCACACAGGGAAA

>HYQZ1MD01AJSTW

CACAGTGCGTGTCGCGTCTCTCAGCACACAGGAGTAG

>HYQZ1MD01DF2EA

CACAGTGCGTGGTCGGCGTCTCTCAAGCACACAGGG

>HYQZ1MD01A7AL0

CACAGTGCGTGGTCGGCGTCTCTCAAGCACACAGGG

>HYQZ1MD01DQCZ8

CACAGTGCGTGTCGCGTCTCTCAGCACACAGGAGAG

>HYQZ1MD01A4GXQ

CACAGTGCGTGGTCGGCGTCTCTCAAGCACACAGGGAAAGG

>HYQZ1MD01C7I53

CACAGTGCGTGGTCGGCGTCTCTCAGCACACAGGGGAAAGG

>HYQZ1MD01BP79B

CACAGTGCGTGTCGGCGTCTCTCAAGCACACAGGG

>HYQZ1MD01BGBFL

CACAGTGCGTGGTCGGCGTCTCTCAAGCACACAGGG

>HYQZ1MD01CJ98P

CACAGTGCGTGTCGCGTCTCTCAAGCACACAGGAGAG

>HYQZ1MD01ANIDB

CACAGTGCGTGGTCGGCGTCTCTCAAGGCACACAGGGGAAAGG

>HYQZ1MD01B2PER

CACAGTGCGTGGTCGGCGTCTCTCAAGGCACACAGGGG

>HYQZ1MD01ANOGK

CACAGTGCGTGGTCGGCGTCTCTCAAGCACACAGGGAAG

>HYQZ1MD01ATKPN

CACAGTGCGTGGTCGCGTCTCTCAAGGCACACAGGGG

>HYQZ1MD01AK0T4

ACAGTGCGTGGTCGCGTCTCTCAAGGCACACAGGGGAAAGG

>HYQZ1MD01DO1FQ

CACAGTGCGTGTCGCGTCTCTCAGCACACAGGAGAG

>HYQZ1MD01CXJMS

ACACAGTGCGTGTCGCGTCTCTCAGCACACAGGAGAG

>HYQZ1MD01BUTPG

CACAGTGCGTGTCGCGTCTCTCAGCACACAGGAGAG

>HYQZ1MD01AE6RN

CACAGTGCGTGGTCGGCGTCTCTCAAGCACACAGGGAA

>HYQZ1MD01AHSY0

CACAGTGCGTGGTCGGCGTCTCTCAAGCACACAGGGAAG

>HYQZ1MD01CFZCH

CACAGTGCGTGGTCGCGTCTCTCAAGCACACAGGGAAG

>HYQZ1MD01B6ZZQ

CACAGTGCGTGGTCGGCGTCTCTCAAGCACACAGGG

>HYQZ1MD01DLI4Q

CACAGTGCGTGGTCGGCGTCTCTCAAGCACACAGGGAAA

>HYQZ1MD01CM8N6

CACAGTGCGTGGTCGGCGTCTCTCAAGCACACAGGG

>HYQZ1MD01A5V5V

CACAGTGCGTGGTCGGCGTCTCTCAAGGCACACAGGGGAAAGG

>HYQZ1MD01CQ913

CACAGTGCGTGGTCGGCGTCTCTCAAGCACACAGGG

>HYQZ1MD01BBL6V

CACAGTGCGTGGTCGGCGTCTCTCAAGCACACAGGGAAAGG

>HYQZ1MD01DAQWK

CACAGTGCGTGGTCGGCGTCTCTCAAGCACACAGGGAAAGG

>HYQZ1MD01BKLZH

CACAGTGCGTGTCGGCGTCTCTCAAGCACACAGGGAA

>HYQZ1MD01DWFC6

CACAGTGCGTGGTCGGCGTCTCTCAAGCACACAGGGAAA

>HYQZ1MD01CZ6AX

CACAGTGCGTGGTCGGCGTCTCTCAAGGCACACAGGGGAAAGG

>HYQZ1MD01BCY2L

CACAGTGCGTGGTCGGCGTCTCTCAAGCACACAGGG

>HYQZ1MD01A62H8

CACAGTGCGTGGTCGGCGTCTCTCAAGGCACACAGGGGAAA

>HYQZ1MD01C7TPM

CACAGTGCGTGGTCGGCGTCTCTCAAGGCACACAGGGGAAAGG

>HYQZ1MD01DACEE

CACAGTGCGTGGTCGGCGTCTCTCAAGCACACAGGGAAGG

>HYQZ1MD01A71M2

CACAGTGCGTGTCGCGTCTCTCAGCACACAGGAGAG

>HYQZ1MD01CIY4L

CACAGTGCGTGGTCGGCGTCTCTCAAGGCACACAGGGGAAAGG

>HYQZ1MD01A6Y5M

CACAGTGCGTGGTCGGCGTCTCTCAAGCACACAGGGAA

>HYQZ1MD01CZX4L

CACAGTGCGTGTCGGCGTCTCTCAAGGCACACAGGGGAAAGG

>HYQZ1MD01CS7TR

CACAGTGCGTGGTCGGCGTCTCTCAAGCACACAGGGAA

>HYQZ1MD01CZD9F

CACAGTGCGTGGTCGGCGTCTCTCAAGGCACACAGGGGAAAGG

>HYQZ1MD01B1POI

CACAGTGCGTGTCGGCGTCTCTCAAGCACACAGGGAAAGG

>HYQZ1MD01DKI1I

CACAGTGCGTGGTCGGCGTCTCTCAAGCACACAGGGAAGG

>HYQZ1MD01AOFP8

CACAGTGCGTGTCGCGTCTCTCAGCACACAGGAGAG

>HYQZ1MD01CWH0H

CACAGTGCGTGGTCGGCGTCTCTCAAGGCACACAGGGGAAA

>HYQZ1MD01CRYWA

CACAGTGCGTGGTCGGCGTCTCTCAAGGCACACAGGGG

>HYQZ1MD01BUPQ7

CACAGTGCGTGGTCGGCGTCTCTCAAGCACACAGGGAA

>HYQZ1MD01C5J4A

CACAGTGCGTGGTCGCGTCTCTCAAGGCACACAGGGG

>HYQZ1MD01DOOUT

CACAGTGCGTGGTCGGCGTCTCTCAAGCACACAGGG

>HYQZ1MD01C147E

CACAGTGCGTGGTCGGCGTCTCTCAAGCACACAGGGAAAGG

>HYQZ1MD01A4HUF

CACAGTGCGTGGTCGCGTCTCTCAAGGCACACAGGGGAAAG

>HYQZ1MD01BN3GG

CACAGTGCGTGGTCGCGTCTCTCAAGGCACACAGGGGAAAGG

>HYQZ1MD01C95H5

CACAGTGCGTGGTCGGCGTCTCTCAAGCACACAGGGAAGG

>HYQZ1MD01DL2MM

CACAGTGCGTGGTCGCGTCTCTCAAGGCACACAGGGGAAAGG

>HYQZ1MD01B8C64

CACAGTGCGTGGTCCGTCTCTCAAGGCACACAGGGGAAAGG

>HYQZ1MD01AYETJ

CACAGTGCGTGGTCGGCGTCTCTCAAGGCACACAGGGG

>HYQZ1MD01C6TH3

CACAGTGCGTGGTCGGCGTCTCTTAAGGCACACAGGGGAAAGG

>HYQZ1MD01CB5ZZ

CACAGTGCGTGTCGCGTCTCTCAGCACACAGGAGAG

>HYQZ1MD01AUWUJ

CACAGTGCGTGGTCGGCGTCTCTCAAGGCACACAGGGG

>HYQZ1MD01AUCAB

CACAGTGCGTGGTCGGCGTCTCTCAAGGCACACAGGGGAAAGG

>HYQZ1MD01CEWSM

CACAGTGCGTGGTCGGCGTCTCTCAAGCACACAGGG

>HYQZ1MD01DB2Y3

CACAGTGCGTGGTCGCGTCTCTCAAGGCACACAGGGG

>HYQZ1MD01CMDU4

CACAGTGCGTGGTCGGCGTCTCTTAAGGCACACAGGGGAAAGG

>HYQZ1MD01BFMR6

CACAGTGCGTGGTCGGCGTCTCTCAGGCACACAGGGG

>HYQZ1MD01B0V85

CACAGTGCGTGTCGCGTCTCTCAAGGCACACAGGGAAAG

>HYQZ1MD01AARFE

ACACAGTGCGTGGTCGGCGTCTCTNCAACGGCACACAGGGAA

>HYQZ1MD01A906B

CACAGTGCGTGGTCGGCGTCTCTCAAGGCACACAGGGGAAAGG

>HYQZ1MD01DS23V

CACAGTGCGTGGTCGCGTCTCTCAAGGCACACAGGGGAAAGG

>HYQZ1MD01BKFQS

CACAGTGCGTGGTCGGCGTCTCTCAAGCACACAGGGAAA

>HYQZ1MD01AO465

CACAGTGCGTGGTCGGCGTCTCTCAAGGCACACAGGGGAAAGG

>HYQZ1MD01C8S8S

ACAGTGCGTGGTCGGCGTCTCTCAAGGCACACAGGGGAAAG

>HYQZ1MD01AL2G7

CACAGTGCGTGTCGGCGTCTCTCAAGGCACACAGGGGAAAGG

>HYQZ1MD01CJCIB

CACAGTGCGTGTCGGCGTCTCTCAAGGCACACAGGGGAAAG

>HYQZ1MD01ATZ39

CACAGTGCGTGTCGGCGTCTCTCAAGCACACAGGGAAG

>HYQZ1MD01AIIXF

CACAGTGCGTGGTCGGCGTCTCTCAAGGCACACAGGGGAAA

>HYQZ1MD01AID8X

CACAGTGCGTGGTCGGCGTCTCTCAAGGCACACAGGGGAAAGG

>HYQZ1MD01AG2DC

CACAGTGCGTGGTCGCGTCTCTCAAGGCACACAGGGG

>HYQZ1MD01AFJJM

CACAGTGCGTGGTCGGCGTCTCTCAAGGCACACAGGGG

>HYQZ1MD01DUJIR

CACAGTGCGTGTCGCGTCTCTCAAGCACACAGGGG

>HYQZ1MD01A0RQK

CACAGTGCGTGTCGCGTCTCTCAAGGCACACAGGGG

>HYQZ1MD01CTRKJ

CACAGTGCGTGGTCGGCGTCTCTCAAGGCACACAGGGGTAAACGG

>HYQZ1MD01AMDKB

CACAGTGCGTGGTCGCGTCTCTCAAGGCACACAGGGGAAAG

>HYQZ1MD01CRKJD

CACAGTGCGTGGTCGGCGTCTCTCAAGGCACACAGGGAAAGG

>HYQZ1MD01BCFED

CACAGTGCGTGGTCGGCGTCTCTCAAGCACACAGGGAAA

>HYQZ1MD01DDC2T

CACAGTGCGTGGTCGCGTCTCTCAAGGCACACAGGGGAAAG

>HYQZ1MD01DECF3

CACAGTGCGTGGTCGGCGTCTCTCAAGCACACAGGGAAGG

>HYQZ1MD01C7ZZU

CACAGTGCGTGGTCGGCGTCTCTCAAGGCACACAGGGGAAAGG

>HYQZ1MD01BAPAR

CACAGTGCGTGGTCGGCGTCTCTCAAGGCACACAGGGG

>HYQZ1MD01CN15L

CACAGTGCGTGGTCGGCGTCTCTCAAGGCACACAGGGGAAAGG

>HYQZ1MD01A5LNL

CACAGTGCGTGGTCGGCGTCTCTCAAGGCACACAGGGG

>HYQZ1MD01B02KR

CACAGTGCGTGGTCGCGTCTCTCAAGGCACACAGGGGAAAG

>HYQZ1MD01B47RT

CACAGTGCGTGGTCGGCGTCTCTCAAGCACACAGGGAAA

>HYQZ1MD01DAJ85

CACAGTGCGTGGTCGGCGTCTCTCAAGCACACAGGGAAGG

>HYQZ1MD01DCIFO

CACAGTGCGTGTCGCGTCTCTCAAGGCACACAGGGG

>HYQZ1MD01A8J7E

CACAGTGCGTGGTCGGCGTCTCTCAAGGCACACAGGGG

>HYQZ1MD01DQ02I

CACAGTGCGTGGTCGGCGTCTCTCAAGGCACACAGGGG

>HYQZ1MD01C6INU

CACAGTGCGTGGTCGGCGTCTCTCAAGGCACACAGGGGAAAGG

>HYQZ1MD01A1XL9

CACAGTGCGTGGTCGGCGTCTCTCAAGGCACACAGGGG

>HYQZ1MD01BNZ9T

CACAGTGCGTGGTCGGCGTCTCTCAAGCACACAGGG

>HYQZ1MD01DJZL7

CACAGTGCGTGGTCGGCGTCTCTCAAGGCACACAGGGG

>HYQZ1MD01CQKRS

CACAGTGCGTGGTCGGCGTCTCTCAAGGCACACAGGGG

>HYQZ1MD01C0KC0

CACAGTGCGTGGTCGGCGTCTCTCAAGCACACAGGGAAA

>HYQZ1MD01AHWE6

CACAGTGCGTGGTCGCGTCTCTCAAGGCACACAGGGG

>HYQZ1MD01BP55G

CACAGTGCGTGGTCGGCGTCTCTCAAGGCACACAGGGGAAAGG

>HYQZ1MD01C7B5H

CACAGTGCGTGTCGCGTCTCTCAAGCACACAGGAGAG

>HYQZ1MD01DJ5ZQ

CACAGTGCGTGTCGCGTCTCTCAAGGCACACAGGGGAAAG

>HYQZ1MD01CLFDV

CACAGTGCGTGGTCGGCGTCTCTCAAGGCACACAGGGGAAAGG

>HYQZ1MD01C7Q4R

CACAGTGCGTGGTCGGCGTCTCTCAAGGCACACAGGGGAAAGG

>HYQZ1MD01DBUTV

CACAGTGCGTGGTCGGCGTCTCTCAAGGCACACAGGGGAAAGG

>HYQZ1MD01AVAU8

CACAGTGCGTGGTCGGCGTCTCTCAAGGCACACAGGGGAAAGG

>HYQZ1MD01BX6Q3

CACAGTGCGTGGTCGGCGTCTCTCAAGGCACACAGGGG

>HYQZ1MD01ALXHA

CACAGTGCGTGGTCGGCGTCTCTCAAGGCACACAGGGGAAAGG

>HYQZ1MD01A5MTF

CACAGTGCGTGGTCGGCGTCTCTCAAGGCACACAGGGGAAAGG

>HYQZ1MD01B95ON

CACAGTGCGTGGTCGGCGTCTCTCAAGGCACACAGGGG

>HYQZ1MD01AKCS9

CACAGTGCGTGGTCGGCGTCTCTCAAGGCACACAGGGGAAAGG

>HYQZ1MD01BJ28A

CACAGTGCGTGGTCGGCGTCTCTTAAGGCACACAGGGGAAAGG

>HYQZ1MD01AYVWB

CACAGTGCGTGGTCGGCGTCTCTCAAGGCACACAGGGGAAAGG

>HYQZ1MD01BLS0U

CACAGTGCGTGGTCGGCGTCTCTCAAGGCACACAGGGGAAAGG

>HYQZ1MD01C2QGB

CACAGTGCGTGTCGGCGTCTCTCAAGGCACACAGGGGAAAG

>HYQZ1MD01AR2YW

CACAGTGCGTGGTCGGCGTCTCTCAAGGCACACAGGGG

>HYQZ1MD01DCFAM

CACAGTGCGTGTCGGCGTCTCTCAAGGCACACAGGGG

>HYQZ1MD01ATAAQ

CACAGTGCGTGTCGGCGTCTCTCAAGGCACACAGGGG

>HYQZ1MD01DLPSY

CACAGTGCGTGGTCGGCTCTCTCAAGGCACACAGGGGAAAGG

>HYQZ1MD01BYWYO

CACAGTGCGTGGTCGGCGTCTCTCAAGGCACACAGGGG

>HYQZ1MD01DBGAG

CACAGTGCGTGGTCGGCGTCTCTCAAGGCACACAGGGGAAAGG

>HYQZ1MD01AIHDB

CACAGTGCGTGGTCGGCGTCTCTCAAGCACACAGGG

>HYQZ1MD01AJNZK

CACAGTGCGTGGTCGGCGTCTCTCAAGGCACACAGGGG

>HYQZ1MD01BGHA5

CACAGTGCGTGGTCGGCGTCTCTCAAGCACACAGGGAAA

>HYQZ1MD01CTMCO

CACAGTGCGTGGTCGGCGTCTCTCAAGCACACAGGGAAA

>HYQZ1MD01DIADZ

CACAGTGCGTGGTCGGCGTCTCTCAAGGCACACAGGGGAAAG

>HYQZ1MD01AHN4V

CACAGTGCGTGGTCGGCGTCTCTCAAGGCACACAGGGG

>HYQZ1MD01BT5YE

CACAGTGCGTGTCGCGTCTCTCAGCACACAGGAGAG

>HYQZ1MD01C468Z

CACAGTGCGTGGTCGCGTCTCTCAAGGCACACAGGGGAAAGG

>HYQZ1MD01C7Q0K

CACAGTGCGTGGTCGGCGTCTCTCAAGGCACACAGGGGAAAGG

>HYQZ1MD01C82NV

CACAGTGCGTGGTCGGCGTCTCTCAAGGCACACAGGGGAAAGG

>HYQZ1MD01DAGN5

CACAGTGCGTGGTCGGCGTCTCTCAAGCACACAGGGAAAGG

>HYQZ1MD01AQT5P

CACAGTGCGTGGTCGGCGTCTCTCAAGCACACAGGGGAAA

>HYQZ1MD01BDAZS

CACAGTGCGTGGTCGGCGTCTCTCAAGGCACACAGGGGAAA

>HYQZ1MD01DG1JV

CACAGTGCGTGGTCGCGTCTCTCAAGGCACACAGGGGAAAGG

>HYQZ1MD01DNJ5K

CACAGTGCGTGGTCGGCGTCTCTCAAGCACACAGGGAAAGG

>HYQZ1MD01DC927

CACAGTGCGTGGTCGGCGTCTCTCAAGGCACACAGGGGAAAGG

>HYQZ1MD01DBZ2N

CACAGTGCGTGGTCGGCGTCTCTCAAGGCACACAGGGG

>HYQZ1MD01CUKPK

CACAGTGCGTGGTCGGCGTCTCTCAAGGCACACAGGGGAAAGG

>HYQZ1MD01CYIVV

CACAGTGCGTGGTCGGCGTCTCTCAAGGCACACAGGGG

>HYQZ1MD01AVCTF

ACAGTGCGTGGTCGGCGTCTCTCAAGGCACACAGGGGAAA

>HYQZ1MD01DJT0K

CACAGTGCGTGGTCGCGTCTCTCAAGGCACACAGGGG

>HYQZ1MD01CL83F

CACAGTGCGTGGTCGGCGTCTCTCAAGGCACACAGGGGAAAGG

>HYQZ1MD01AR6TT

CACAGTGCGTGGTCGGCGTCTCTCAAGGCACACAGGGG

>HYQZ1MD01AXWY4

CACAGTGCGTGGTCGGCGTCTCTCAAGGCACACAGGGGAAAGG

>HYQZ1MD01AOCN8

ACAGTGCGTGGTCGGCGTCTCTCAAGGCACACAGGGGAAAGG

>HYQZ1MD01AL4RS

ACAGTGCGTGGTCGGCGTCTCTCAAGGCACACAGGGG

>HYQZ1MD01BFPED

CACAGTGCGTGGTCGGCGTCTCTCAAGGCACACAGGGGAAAGG

>HYQZ1MD01DJDU9

CACAGTGCGTGGTCGGCGTCTCTCAAGGCACACAGGGGAAAGG

>HYQZ1MD01CO38I

CACAGTGCGTGGTCGGCGTCTCTCAAGGCACACAGGGGAAAGG

>HYQZ1MD01BHXGK

CACAGTGCGTGGTCGGCGTCTCTCAAGGCACACAGGGGAAAGG

>HYQZ1MD01DS6A2

CACAGTGCGTGGTCGCGTCTCTCAAGGCACACAGGGGAAAGG

>HYQZ1MD01BTIVA

CACAGTGCGTGGTCGGCGTCTCTCAAGGCACACAGGGGAAAGG

>HYQZ1MD01BT1UA

CACAGTGCGTGGTCGCGTCTCTCAAGGCACACAGGGGAAAGG

>HYQZ1MD01AOE0E

CACAGTGCGTGGTCGGCGTCTCTCAAGGCACACAGGGGAAAGG

>HYQZ1MD01DPLHN

CACAGTGCGTGGTCGGCGTCTCTCAAGGCACACAGGGGAAA

>HYQZ1MD01DQGIG

CACAGTGCGTGGTCGGCGTCTCTCAAGGCACACAGGGGAAA

>HYQZ1MD01DOZON

CACAGTGCGTGGTCGGCGTCTCTCAAGGCACACAGGGGAAAGG

>HYQZ1MD01DKKGA

CACAGTGCGTGTCGGCGTCTCTCAAGGCACACAGGGG

>HYQZ1MD01BJJQ8

ACAGTGCGTGGTCGGCGTCTCTCAAGGCACACAGGGG

>HYQZ1MD01AL7IC

CACAGTGCGTGTCGCGTCTCTCAAGGCACACAGGGG

>HYQZ1MD01DJBXT

CACAGTGCGTGGTCGGCGTCTCTCAAGGCACACAGGGGAAAGG

>HYQZ1MD01A7VG9

CACAGTGCGTGTCGCGTCTCTCAAGCACACAGGGGAAAG

>HYQZ1MD01A7FUF

CACAGTGCGTGGTCGGCGTCTCTCAAGGCACACAGGGG

>HYQZ1MD01CQEMD

CACAGTGCGTGGTCGGCGTCTCTCAAGGCACACAGGGG

>HYQZ1MD01CRBGY

CACAGTGCGTGGTCGGCGTCTCTCAAGGCACACAGGGGAAAGG

>HYQZ1MD01DJ82W

CACAGTGCGTGGTCGGCGTCTCTCAAGCACACAGGGAAAGG

>HYQZ1MD01DWDXC

CACAGTGCGTGGTCGGCGTCTCTCAAGGCACACAGGGGAAAGG

>HYQZ1MD01CP2ZQ

CACAGTGCGTGGTCGGCGTCTCTCAAGGCACACAGGGGAAAGG

>HYQZ1MD01DR52D

CACAGTGCGTGGTCGGCGTCTCTCAAGGCACACAGGGG

>HYQZ1MD01AOMR7

ACAGTGCGTGGTCGCGTCTCTCAAGGCACACAGGGGAAAG

>HYQZ1MD01DC6G8

CACAGTGCGTGGTCGGCGTCTCTCAAGCACACAGGGAAAGG

>HYQZ1MD01CBXNI

CACAGTGCGTGTCGGCGTCTCTCAAGGCACACAGGGGAAAGG

>HYQZ1MD01A75IH

CACAGTGCGTGGTCGGCGTCTCTCAAGGCACACAGGGG

>HYQZ1MD01C9MSE

CACAGTGCGTGGTCGGCGTCTCTCAAGGCACACAGGGGAAA

>HYQZ1MD01DCI8H

CACAGTGCGTGGTCGGCGTCTCTCAAGGCACACAGGGGAAAGG

>HYQZ1MD01BM0G1

CACAGTGCGTGGTCGGCGTCTCTCAAGCACACAGGGAAGG

>HYQZ1MD01B2IYO

CACAGTGCGTGGTCGGCGTCTCTCAAGGCACACAGGGG

>HYQZ1MD01BXRV4

CACAGTGCGTGGTCGGCGTCTCTCAAGGCACACAGGGGAAAGG

>HYQZ1MD01AJHSH

CACAGTGCGTGGCCGGCGTCTCTCAAGGCACACAGGGGAAAGG

>HYQZ1MD01AJ98W

CACAGTGCGTGGTCGGCGTCTCTCAAGGCACACAGGGG

>HYQZ1MD01CCIAP

CACAGTGCGTGTCGCGTCTCTCAAGGCACACAGGGGAAA

>HYQZ1MD01AFLZ0

CACAGTGCGTGGTCGGCGTCTCTCAAGCACACAGGGAAA

>HYQZ1MD01AFCB9

CACAGTGCGTGGTCGGCGTCTCTCAAGGCACACAGGGG

>HYQZ1MD01A1FF4

CACAGTGCGTGGTCGGCGTCTCTCAAGGCACACAGGGGAAAGG

>HYQZ1MD01DL8WG

CACAGTGCGTGGTCGGCGTCTCTCAAGCACACAGGGAAAGG

>HYQZ1MD01DIGD8

CACAGTGCGTGTCGCGTCTCTCAAGGCACACAGGGGAAAG

>HYQZ1MD01BUX48

CACAGTGCGTGGTCGCGTCTCTCAAGGCACACAGGGGAAAG

>HYQZ1MD01C5S7V

CACAGTGCGTGGTCGGCGTCTCTCAAGGCACACAGGGGAAAGG

>HYQZ1MD01BY5M3

CACAGTGCGTGTCGCGTCTCTCAAGGCACACAGGGG

>HYQZ1MD01AIYIM

ACAGTGCGTGGTCGGCGTCTCTCAAGGCACACAGGGG

>HYQZ1MD01DQUHE

CACAGTGCGTGGTCGGCGTCTCTCAAGGCACACAGGGGAAAGG

>HYQZ1MD01ATLNM

CACAGTGCGTGGTCGGCGTCTCTCAAGGCACACAGGGGAAA

>HYQZ1MD01AVEN1

CACAGTGCGTGGTCGGCGTCTCTCAAGGCACACAGGGGAAAGG

>HYQZ1MD01AZTX3

CACAGTGCGTGGTCGGCGTCTCTCAAGGCACACAGGGG

>HYQZ1MD01AVXS3

CACAGTGCGTGGTCGGCGTCTCTCAAGGCACACAGGGG

>HYQZ1MD01ABCAH

CACAGTGCGTGGTCGGCGTCTCTCAAGCACACAGGGAAAGG

>HYQZ1MD01AY48Z

CACAGTGCGTGTCGCGTCTCTCAAGCACACAGGGGAAAG

>HYQZ1MD01CBMAL

CACAGTGCGTGGTCGGCGTCTCTCAAGGCACACAGGGGAAAGG

>HYQZ1MD01CQKZM

CACAGTGCGTGGTCGGCGTCTCTCAAGGCACACAGGGG

>HYQZ1MD01AQW4T

CACAGTGCGTGGTCGGCGTCTCTTAAGGCACACAGGGGAAAGG

>HYQZ1MD01BT1QE

CACAGTGCGTGGTCGGCGTCTCTCAAGCACACAGGGAAA

>HYQZ1MD01A3X34

CACAGTGCGTGGTCGGCGTCTCTCAAGGCACACAGGGGAAA

>HYQZ1MD01DN5B6

CACAGTGCGTGGTCGGCGTCTCTCAAGGCACACAGGGGAAAGG

>HYQZ1MD01BB2CI

CACAGTGCGTGGTCGGCGTCTCTCAAGGCACACAGGGGAAAGG

>HYQZ1MD01C4XF0

CACAGTGCGTGGTCGGCGTCTCTCAAGGCACACAGGGGAAAGG

>HYQZ1MD01BO0XO

CACAGTGCGTGGTCGCGTCTCTCAAGGCACACAGGGG

>HYQZ1MD01B7IJP

CACAGTGCGTGTCGCGTCTCTCAAGCACACAGGGGAAAG

>HYQZ1MD01AGLYJ

CACAGTGCGTGGTCGCGTCTCTCAAGGCACACAGGGGAAAGG

>HYQZ1MD01ARIXX

CACAGTGCGTGGTCGGCGTCTCTCAAGGCACACAGGGGAAAGG

>HYQZ1MD01C9Q9J

CACAGTGCGTGGTCGGCGTCTCTCAAGGCACACAGGGGAAAGG

>HYQZ1MD01CH9P5

CACAGTGCGTGTCGGCGTCTCTCAAGCACACAGGGAAGG

>HYQZ1MD01B0QTW

CACAGTGCGTGGTCGGCGTCTCTCAAGGCACACAGGGG

>HYQZ1MD01BRKUL

CACAGTGCGTGGTCGGCGTCTCTCAAGGCACACAGGGGAAAGG

>HYQZ1MD01CXHD6

CACAGTGCGTGTCGCGTCTCTCAAGGCACACAGGGGAAA

>HYQZ1MD01AJ9YP

CACAGTGCGTGGTCGCGTCTCTCAAGGCACACAGGGG

>HYQZ1MD01C0M39

CACAGTGCGTGGTCGGCGTCTCTCAAGGCACACAGGGGAAAGG

>HYQZ1MD01AJYOT

CACAGTGCGTGGTCGGCGTCTCTCAAGGCACACAGGGGAAAGG

>HYQZ1MD01BTZ3I

CACAGTGCGTGGTCGGCGTCTCTCAAGCACACAGGGAAGG

>HYQZ1MD01BNZV8

CACAGTGCGTGGTCGGCGTCTCTCAAGGCACACAGGGGAAA

>HYQZ1MD01DKLF9

CACAGTGCGTGGTCGGCGTCTCTCAAGGCACACAGGGGAAAGG

>HYQZ1MD01C7H0G

CACAGTGCGTGTCGCGTCTCTCAAGGCACACAGGGGAAA

>HYQZ1MD01BKG1N

CACAGTGCGTGGTCGGCGTCTCTCAAGGCACACAGGGGAAAGG

>HYQZ1MD01BW5F7

CACAGTGCGTGGTCGGCGTCTCTCAAGGCACACAGGGGAAAGG

>HYQZ1MD01AQ9GP

CACAGTGCGTGGTCGGCGTCTCTCAAGGCACACAGGGGAAAGG

>HYQZ1MD01B3NHJ

CACAGTGCGTGGTCGGCGTCTCTCAAGGCACACAGGGG

>HYQZ1MD01CKLH6

CACAGTGCGTGGTCGGCGTCTCTCAAGGCACACAGGGGAAA

>HYQZ1MD01CMW2I

CACAGTGCGTGGTCGGCGTCTCTCAAGGCACACAGGGGAAAGG

>HYQZ1MD01C01LQ

CACAGTGCGTGGTCGGCGTCTCTCAAGGCACACAGGGG

>HYQZ1MD01BWWGC

CACAGTGCGTGGTCGGCGTCTCTCAAGGCACACAGGGGAAAGG

>HYQZ1MD01CSTZX

CACAGTGCGTGGTCGGCGTCTCTCAAGGCACACAGGGGAAAGG

>HYQZ1MD01AWBI0

CACAGTGCGTGGTCGGCGTCTCTCAAGGCACACAGGGGTAAA

>HYQZ1MD01DI50D

CACAGTGCGTGGTCGGCGTCTCTCAAGCACACAGGGG

>HYQZ1MD01BROOD

CACAGTGCGTGGTCGGCGTCTCTCAAGGCACACAGGGGAAA

>HYQZ1MD01B4VUV

CACAGTGCGTGGTCGGCGTCTCTCAAGGCACACAGGGGAAAGG

>HYQZ1MD01DDQXG

CACAGTGCGTGGTCGGCGTCTCTCAAGGCACACAGGGGAAAGG

>HYQZ1MD01BQ19N

CACAGTGCGTGGTCGGCGTCTCTCAAGCACACAGGG

>HYQZ1MD01DBUAF

CACAGTGCGTGGTCGGCGTCTCTAACGGCACACAGGGAAGGAG

>HYQZ1MD01CBG3C

CACAGTGCGTGGTCGGCGTCTCTCAAGGCACACAGGGG

>HYQZ1MD01DAU9I

CACAGTGCGTGGTCGGCGTCTCTCAAGGCACACAGGGG

>HYQZ1MD01APBR9

CACAGTGCGTGGTCGGCGTCTCTCAAGGCACACAGGGG

>HYQZ1MD01CQFJ2

CACAGTGCGTGGTCGGCGTCTCTCAAGGCACACAGGGG

>HYQZ1MD01BYJN9

CACAGTGCGTGGTCGGCGTCTCTCAAGCACACAGGGAAA

>HYQZ1MD01AUK90

CACAGTGCGTGGTCGGCGTCTCTCAAGCACACAGGGAAAGG

>HYQZ1MD01BT6GO

CACAGTGCGTGGTCGGCGTCTCTCAAGGCACACAGGGG

>HYQZ1MD01ACKQM

CACAGTGCGTGGTCGGCGTCTCTCAAGGCACACAGGGGAAAGG

>HYQZ1MD01DV8SG

CACAGTGCGTGGTCGGCGTCTCTCAAGGCACACAGGGG

>HYQZ1MD01AKLKC

CACAGTGCGTGGTCGGCGTCTCTCAAGCACACAGGGG

>HYQZ1MD01B2WZF

CACAGTGCGTGGTCGGCGTCTCTCAAGGCACACAGGGG

>HYQZ1MD01C446Z

CACAGTGCGTGGTCGGCGTCTCTCAAGGCACACAGGGGAAAGG

>HYQZ1MD01B3XAL

CACAGTGCGTGGTCGGCGTCTCTCAAGGCACACAGGGGAAAGG

>HYQZ1MD01BKQI2

CACAGTGCGTGGTCGGCGTCTCTCAAGGCACACAGGGGAAAGG

>HYQZ1MD01DBBTV

CACAGTGCGTGGTCGGCGTCTCTCAAGGCACACAGGGGAAAGG

>HYQZ1MD01C7H5A

CACAGTGCGTGGTCGGCGTCTCTCAAGCACACAGGGAAG

>HYQZ1MD01CTVGR

CACAGTGCGTGGTCGGCGTCTCTCAAGGCACACAGGGG

>HYQZ1MD01ALHW4

CACAGTGCGTGGTCGCGTCTCTCAAGGCACACAGGGG

>HYQZ1MD01CLV0M

CACAGTGCGTGGTCGGCGTCTCTCAAGGCACACAGGGG

>HYQZ1MD01DA8O4

CACAGTGCGTGGTCGGCGTCTCTCAAGGCACACAGGGGAAAGG

>HYQZ1MD01B48OP

CACAGTGCGTGGTCGGCGTCTCTCAAGGCACACAGGGG

>HYQZ1MD01BSJSC

CACAGTGCGTGGTCGGCGTCTCTCAAGGCACACAGGGGAAAGG

>HYQZ1MD01DS4JO

CACAGTGCGTGTCGCGTCTCTCAAGGCACACAGGGGAAA

>HYQZ1MD01C625F

CACAGTGCGTGTCGGCGTCTCTCAAGGCACACAGGGG

>HYQZ1MD01C4U50

CACAGTGCGTGGTCGGCGTCTCTCAAGGCACACAGGGGAAAGG

>HYQZ1MD01BHDT5

CACAGTGCGTGGTCGGCGTCTCTCAAGCACACAGGG

>HYQZ1MD01DJAO7

ACAGTGCGTGGTCGGCGTCTCTCAAGGCACACAGGGGAAA

>HYQZ1MD01DMON6

CACAGTGCGTGGTCGGCGTCTCTCAAGCACACAGGGAAAGG

>HYQZ1MD01AQSHZ

CACAGTGCGTGGTCGGCGTCTCTCAAGGCACACAGGGGAAAGG

>HYQZ1MD01DHYKU

CACAGTGCGTGGTCGGCGTCTCTCAAGGCACACAGGGGAAA

>HYQZ1MD01AKJYR

CACAGTGCGTGGTCGGCGTCTCTCAAGGCACACAGGGG

>HYQZ1MD01AKRWO

CACAGTGCGTGGTCGGCGTCTCTCAAGGCACACAGGGG

>HYQZ1MD01ANMLR

CACAGTGCGTGGTCGGCGTCTCTCAAGGCACACAGGGGAAAGG

>HYQZ1MD01BKAY4

CACAGTGCGTGGTCGCGTCTCTCAAGGCACACAGGGGAAAGG

>HYQZ1MD01CNJKE

CACAGTGCGTGGTCGGCGTCTCTCAAGGCACACAGGGGAAAGG

>HYQZ1MD01A27X5

CACAGTGCGTGGTCGGCGTCTCTCAAGGCACACAGGGGAAAGG

>HYQZ1MD01CWYGB

CACAGTGCGTGGTCGGCGTCTCTCAAGGCACACAGGGGAAAGG

>HYQZ1MD01DAMDT

CACAGTGCGTGGTCGGCGTCTCTCAAGGCACACAGGGGAAAGG

>HYQZ1MD01CJB1Q

CACAGTGCGTGGTCGGCGTCTCTCAAGGCACACAGGGGAAAGG

>HYQZ1MD01AKQAE

CACAGTGCGTGTCGCGTCTCTCAAGCACACAGGGG

>HYQZ1MD01ATXSF

CACAGTGCGTGGTCGGCGTCTCTCAAGGCACACAGGGGAAAGG

>HYQZ1MD01C8468

CACAGTGCGTGGTCGGCGTCTCTCAAGGCACACAGGGGAAAGG

>HYQZ1MD01BKQOK

CACAGTGCGTGGTCGGCGTCTCTCAAGGCACACAGGGGAAAGG

>HYQZ1MD01C4Q5T

CACAGTGCGTGGTCGGCGTCTCTCAAGGCACACAGGGGAAAGG

>HYQZ1MD01C3NM0

CACAGTGCGTGGTCGGCGTCTCTCAAGGCACACAGGGGAAAGG

>HYQZ1MD01DLN7F

CACAGTGCGTGGTCGGCGTCTCTCAAGGCACACAGGGGAAAGG

>HYQZ1MD01ARGTG

CACAGTGCGTGGTCGGCGTCTCTCAAGGCACACAGGGGAAAGG

>HYQZ1MD01AWCJA

CACAGTGCGTGGTCGGCGTCTCTCAAGCACACAGGGAAAGG

>HYQZ1MD01BQU9R

ACAGTGCGTGGTCGGCGTCTCTCAAGGCACACAGGGGAAAGG

>HYQZ1MD01DG71G

CACAGTGCGTGGTCGCGTCTCTCAAGGCACACAGGGGAAAGG

>HYQZ1MD01AUSMT

CACAGTGCGTGGTCGGCGTCTCTCAAGCACACAGGG

>HYQZ1MD01BS74I

CACAGTGCGTGTCGGCGTCTCTCAAGGCACACAGGGGAAAGG

>HYQZ1MD01ARJPY

CACAGTGCGTGGTCGGCGTCTCTCAAGGCACACAGGGGAAAG

>HYQZ1MD01CY5CB

CACAGTGCGTGGTCGGCGTCTCTCAAGGCACACAGGGGAAAGG

>HYQZ1MD01ATB6E

CACAGTGCGTGGTCGGCGTCTCTCAAGGCACACAGGGGAAA

>HYQZ1MD01A6RCV

CACAGTGCGTGGTCGCGTCTCTCAAGGCACACAGGGGAAAG

>HYQZ1MD01DOSQI

CACAGTGCGTGGTCGGCGTCTCTCAAGGCACACAGGGGAAAGG

>HYQZ1MD01AMWEA

CACAGTGCGTGGTCGGCGTCTCTCAAGGCACACAGGGGAAAGG

>HYQZ1MD01A98ZV

CACAGTGCGTGGTCGGCGTCTCTCAAGGCACACAGGGGAAAGG

>HYQZ1MD01BH6TP

CACAGTGCGTGGTCGGCGTCTCTCAAGCACACAGGGAAAGG

>HYQZ1MD01CCIAQ

CACAGTGCGTGTCGCGTCTCTCAAGGCACACAGGGGAAA

>HYQZ1MD01C6YEQ

CACAGTGCGTGGTCGGCGTCTCTCAAGGCACACAGGGGAAAGG

>HYQZ1MD01DR8IP

CACAGTGCGTGGTCGGCGTCTCTCAAGGCACACAGGGGAAAGG

>HYQZ1MD01AXNQB

CACAGTGCGTGGTCGGCGTCTCTTAAGGCACACAGGGGAAAGG

>HYQZ1MD01AW17U

CACAGTGCGTGGTCGGCGTCTCTCAAGGCACACAGGGGAAAGG

>HYQZ1MD01A0VVC

CACAGTGCGTGGTCGGCGTCTCTCAAGGCACACAGGGGTAAA

>HYQZ1MD01CS5ZK

CACAGTGCGTGGTCGGCGTCTCTCAAGGCACACAGGGGAAAGG

>HYQZ1MD01BYPDQ

CACAGTGCGTGGTCGGCGTCTCTCAAGGCACACAGGGGAAAGG

>HYQZ1MD01DNO74

CACAGTGCGTGGTCGGCGTCTCTCAAGGCACACAGGGGAAA

>HYQZ1MD01DAWT2

CACAGTGCGTGGTCGGCGTCTCTCAAGGCACACAGGGGAAAGG

>HYQZ1MD01CTWJY

CACAGTGCGTGGTCGGCGTCTCTCAAGGCACACAGGGG

>HYQZ1MD01B6GYZ

CACAGTGCGTGGTCGCGTCTCTCAAGGCACACAGGGGAAAGG

>HYQZ1MD01BLFKJ

CACAGTGCGTGGTCGCGTCTCTCAAGGCACACAGGGGAAAG

>HYQZ1MD01BY44G

CACAGTGCGTGGTCGGCGTCTCTCAAGGCACACAGGGGAAAGG

>HYQZ1MD01BZCLK

CACAGTGCGTGGTCGGCGTCTCTCAAGGCACACAGGGGAAAGG

>HYQZ1MD01CITBL

CACAGTGCGTGGTCGGCGTCTCTCAAGGCACACAGGGGAAAGG

>HYQZ1MD01AWCM5

CACAGTGCGTGGTCGGCGTCTCTCAAGGCACACAGGGGAAAGG

>HYQZ1MD01DDRPA

CACAGTGCGTGGTCGGCGTCTCTCAAGGCACACAGGGGAAA

>HYQZ1MD01B4XUM

CACAGTGCGTGGTCGGCGTCTCTCAAGGCACACAGGGGAAAGG

>HYQZ1MD01BZWYM

CACAGTGCGTGGTCGGCGTCTCTCAAGGCACACAGGGGAAAGG

>HYQZ1MD01BR22J

CACAGTGCGTGGTCGGCGTCTCTCAAGGCACACAGGGGAAAGG

>HYQZ1MD01DUQ4T

CACAGTGCGTGGTCGGCGTCTCTCAAGGCACACAGGGGAAAGG

>HYQZ1MD01DF44P

CACAGTGCGTGGTCGGCGTCTCTCAAGGCACACAGGGGAAAGG

>HYQZ1MD01DCJTF

CACAGTGCGTGGTCGGCGTCTCTCAAGGCACACAGGGG

>HYQZ1MD01AFYFU

CACAGTGCGTGGTCGGCGTCTCTCAAGGCACACAGGGGAAAGG

>HYQZ1MD01BOBHM

CACAGTGCGTGTCGGCGTCTCTCAAGGCACACAGGGGAAAGG

>HYQZ1MD01DJOVP

CACAGTGCGTGGTCGCGTCTCTCAAGGCACACAGGGGAAAGG

>HYQZ1MD01AXTMU

CACAGTGCGTGGTCGGCGTCTCTCAAGGCACACAGGGGAAAGG

>HYQZ1MD01BANH4

CACAGTGCGTGGTCGGCGTCTCTCAAGGCACACAGGGGAAAGG

>HYQZ1MD01DOF3E

CACAGTGCGTGGTCGGCGTCTCTCAAGGCACACAGGGGAAAGG

>HYQZ1MD01CQ4LU

CACAGTGCGTGGTCGGCGTCTCTCAAGCACACAGGG

>HYQZ1MD01AYRVL

CACAGTGCGTGGTCGGCGTCTCTCAAGCACACAGGGAAGG

>HYQZ1MD01CL2W8

CACAGTGCGTGTCGGCGTCTCTCAAGGCACACAGGGGAAA

>HYQZ1MD01CE1XZ

CACAGTGCGTGGTCGGCGTCTCTCAAGGCACACAGGGGAAAGG

>HYQZ1MD01AVVYF

CACAGTGCGTGGTCGGCGTCTCTCAAGGCACACAGGGGAAAGG

>HYQZ1MD01CSJNR

ACAGTGCGTGTCGCGTCTCTCAAGGCACACAGGGGAAAGG

>HYQZ1MD01CW6NW

CACAGTGCGTGGTCGGCGTCTCTCAAGGCACACAGGGGAAAGG

>HYQZ1MD01CMCZ2

CACAGTGCGTGGTCGGCGTCTCTCAAGGCACACAGGGG

>HYQZ1MD01CFPU1

ACAGTGCGTGGTCGGCGTCTCTCAAGGCACACAGGGG

>HYQZ1MD01AWR1T

CACAGTGCGTGGTCGGCGTCTCTCAAGGCACACAGGGGG

>HYQZ1MD01BSRG7

CACAGTGCGTGGTCGGCGTCTCTCAAGGCACACAGGGG

>HYQZ1MD01A0I9A

CACAGTGCGTGTCGGCGTCTCTCAAGGCACACAGGGGTAAA

>HYQZ1MD01AJR3I

CACAGTGCGTGGTCGGCGTCTCTCAAGGCACACAGGGG

>HYQZ1MD01BOCWX

CACAGTGCGTGTCGCGTCTCTCAAGGCACACAGGGGAAAG

>HYQZ1MD01CS5BU

CACAGTGCGTGGTCGGCGTCTCTCAGGCACACAGGGG

>HYQZ1MD01ANYXN

ACAGTGCGTGGTCGGCGTCTCTCAAGGCACACAGGGG

>HYQZ1MD01AG0IL

CACAGTGCGTGGTCGGCGTCTCTCAAGGCACACAGGGGAAAGG

>HYQZ1MD01DAMZT

CACAGTGCGTGGTCGGCGTCTCTCAAGGCACACAGGGGAAAGG

>HYQZ1MD01CH5EX

CACAGTGCGTGGTCGGCGTCTCTCAAGGCACACAGGGG

>HYQZ1MD01BBMLE

CACAGTGCGTGTCGCGTCTCTCAAGGCACACAGGGG

>HYQZ1MD01CMEXL

CACAGTGCGTGGTCGGCGTCTCTCAAGGCACACAGGGGAAAGG

>HYQZ1MD01DS9UV

CACAGTGCGTGGTCGGCGTCTCTCAAGGCACACAGGGGAAAGG

>HYQZ1MD01ARIJQ

CACAGTGCGTGGTCGGCGTCTCTCAAGGCACACAGGGGAAAGG

>HYQZ1MD01AJCK2

CACAGTGCGTGGTCGGCGTCTCTCAAGGCACACAGGGGAAAGG

>HYQZ1MD01AKGZ1

CACAGTGCGTGGTCGGCGTCTCTCAAGGCACACAGGGGAAAGG

>HYQZ1MD01BY7O8

CACAGTGCGTGGTCGGCGTCTCTCAAGGCACACAGGGGAAAGG

>HYQZ1MD01BPOB0

CACAGTGCGTGTCGGCGTCTCTCAAGGCACACAGGGGAAA

>HYQZ1MD01BJSQG

CACAGTGCGTGGTCGGCGTCTCTCAAGGCACACAGGGGAAAGG

>HYQZ1MD01CPZOF

CACAGTGCGTGGTCGGCGTCTCTCAAGGCACACAGGGG

>HYQZ1MD01DFIOD

CACAGTGCGTGGTCGGCGTCTCTCAAGGCACACAGGGGAAAGG

>HYQZ1MD01DUF65

CACAGTGCGTGGTCGGCGTCTCTCAAGGCACACAGGGG

>HYQZ1MD01C34Z0

CACAGTGCGTGGTCGGCGTCTCTCAAGGCACACAGGGGAAAGG

>HYQZ1MD01ACV66

CACAGTGCGTGGTCGGCGTCTCTCAAGGCACACAGGGGAAAGG

>HYQZ1MD01CAGU8

ACAGTGCGTGGTCGGCGTCTCTCAAGGCACACAGGGGAAAGG

>HYQZ1MD01AVIO9

CACAGTGCGTGGTCGGCGTCTCTCAAGGCACACAGGGGAAAGG

>HYQZ1MD01CLU55

CACAGTGCGTGGTCGGCGTCTCTCAAGGCACACAGGGGAAAGG

>HYQZ1MD01BGW3U

CACAGTGCGTGGTCGGCGTCTCTCAAGCACACAGGG

>HYQZ1MD01C2RL1

CACAGTGCGTGGTCGGCGTCTCTCAAGGCACACAGGGG

>HYQZ1MD01DTU99

CACAGTGCGTGGTCGGCGTCTCTCAAGGCACACAGGGGAAAGG

>HYQZ1MD01AI3EG

CACAGTGCGTGGTCGCGTCTCTCAAGGCACACAGGGGAAAG

>HYQZ1MD01AOOJM

CACAGTGCGTGGTCGGCGTCTCTCAAGGCACACAGGGG

>HYQZ1MD01AH92M

CACAGTGCGTGGTCGGCGTCTCTCAAGGCACACAGGGGTAAA

>HYQZ1MD01DSZU2

CACAGTGCGTGGTCGGCGTCTCTCAAGGCACACAGGGGAAAGG

>HYQZ1MD01AX996

CACAGTGCGTGGTCGGCGTCTCTCAAGGCACACAGGGGAAAGG

>HYQZ1MD01DH655

CACAGTGCGTGGTCGGCGTCTCTCAAGGCACACAGGGGAAAGG

>HYQZ1MD01BX9SC

CACAGTGCGTGGTCGGCGTCTCTCAAGGCACACAGGGG

>HYQZ1MD01C0R50

CACAGTGCGTGGTCGGCGTCTCTCAAGGCACACAGGGGAAAGG

>HYQZ1MD01B7L4W

CACAGTGCGTGGTCGGCGTCTCTCAAGGCACACAGGGGAAAGG

>HYQZ1MD01AGUXB

CACAGTGCGTGGTCGGCGTCTCTCAAGGCACACAGGGGAAAGG

>HYQZ1MD01A57SJ

CACAGTGCGTGGTCGGCGTCTCTCAAGGCACACAGGGGAAAGG

>HYQZ1MD01CBJQ3

CACAGTGCGTGGTCGGCGTCTCTCAAGCACACAGGGAAAGG

>HYQZ1MD01AU7PY

CACAGTGCGTGGTCGCGTCTCTCAAGGCACACAGGGGAAAG

>HYQZ1MD01CIT93

CACAGTGCGTGGTCGGCGTCTCTCAAGGCACACAGGGG

>HYQZ1MD01AF1OJ

CACAGTGCGTGGTCGGCGTCTCTCAAGGCACACAGGGGAAAGG

>HYQZ1MD01DU3SR

CACAGTGCGTGGTCGGCGTCTCTCAAGCACACAGGGAAAGG

>HYQZ1MD01CIDBG

CACAGTGCGTGGTCGGCGTCTCTCAAGCACACAGGGAAGG

>HYQZ1MD01DDRXK

CACAGTGCGTGGTCGGCGTCTCTCAAGCACACAGGGAAAGG

>HYQZ1MD01CZH8V

CACAGTGCGTGGTCGGCGTCTCTCAAGGCACACAGGGG

>HYQZ1MD01BU65Z

CACAGTGCGTGGTCGGCGTCTCTCAAGGCACACAGGGGAAAGG

>HYQZ1MD01AJKRX

CACAGTGCGTGGTCGGCGTCTCTCAAGGCACACAGGGG

>HYQZ1MD01AC1KQ

CACAGTGCGTGGTCGGCGTCTCTCAAGGCACACAGGGG

>HYQZ1MD01DBIH3

CACAGTGCGTGGTCGGCGTCTCTCAAGGCACACAGGGGAAAGG

>HYQZ1MD01DHG6N

CACAGTGCGTGGTCGCGTCTCTCAAGGCACACAGGGGAAAG

>HYQZ1MD01DFP6Y

CACAGTGCGTGGTCGGCGTCTCTCAAGGCACACAGGGG

>HYQZ1MD01CRF21

CACAGTGCGTGGTCGGCGTCTCTCAAGGCACACAGGGGAAAGG

>HYQZ1MD01DIILH

CACAGTGCGTGGTCGGCGTCTCTCAAGGCACACAGGGG

>HYQZ1MD01BMXAU

CACAGTGCGTGGTCGGCGTCTCTCAAGGCACACAGGGGAAAGG

>HYQZ1MD01CM6OE

CACAGTGCGTGGTCGGCGTCTCTCAAGGCACACAGGGGAAAGG

>HYQZ1MD01BTPMY

CACAGTGCGTGGTCGGCGTCTCTCAAGGCACACAGGGGAAAGG

>HYQZ1MD01A9UT2

CACAGTGCGTGTCGGCGTCTCTCAAGGCACACAGGGGAAAGG

>HYQZ1MD01DT3NX

CACAGTGCGTGGTCGGCGTCTCTCAAGCACACAGGGAAGG

>HYQZ1MD01DAMDL

CACAGTGCGTGGTCGGCGTCTCTCAAGGCACACAGGGGAAAGG

>HYQZ1MD01BNAB5

CACAGTGCGTGGTCGGCGTCTCTCAAGGCACACAGGGGAAAGG

>HYQZ1MD01B0W5X

CACAGTGCGTGGTCGGCGTCTCTCAAGGCACACAGGGGAAAGG

>HYQZ1MD01CRA1P

CACAGTGCGTGGTCGGCGTCTCTCTAGGCACACAGGGGAAAGG

>HYQZ1MD01DSYDF

CACAGTGCGTGGTCGGCGTCTCTCAAGGCACACAGGGGAAAGG

>HYQZ1MD01CSGZU

CACAGTGCGTGGTCGGCGTCTCTCAAGCACACAGGGAAA

>HYQZ1MD01B13Q0

CACAGTGCGTGGTCGGCGTCTCTCAAGGCACACAGGGG

>HYQZ1MD01B3MEG

CACAGTGCGTGGTCGGCGTCTCTCAAGCACACAGGGAAAGG

>HYQZ1MD01BOPU2

CACAGTGCGTGGTCGGCGTCTCTCAAGGCACACAGGGG

>HYQZ1MD01BI3DG

CACAGTGCGTGTCGGCGTCTCTCAAGGCACACAGGGG

>HYQZ1MD01B1YB1

CACAGTGCGTGGTCGGCGTCTCTCAAGGCACACAGGGGAAAGG

>HYQZ1MD01BHHKE

CACAGTGCGTGGTCGGCGTCTCTCAAGGCACACAGGGGAAAGG

>HYQZ1MD01BMCR6

CACAGTGCGTGGTCGGCGTCTCTCAAGGCACACAGGGGAAAGG

>HYQZ1MD01AZJP4

CACAGTGCGTGGTCGGCGTCTCTCAAGGCACACAGGGGAAAGG

>HYQZ1MD01CRIIL

CACAGTGCGTGGTCGGCGTCTCTCAAGGCACACAGGGGAAA

>HYQZ1MD01BCH6L

CACAGTGCGTGGTCGGCGTCTCTCAAGGCACACAGGGG

>HYQZ1MD01AC6L2

CACAGTGCGTGGTCGGCGTCTCTCAAGGCACACAGGGG

>HYQZ1MD01BQ5MN

CACAGTGCGTGGTCGGCGTCTCTCAAGGCACACAGGGGAAAGG

>HYQZ1MD01AOKK7

CACAGTGCGTGGTCGGCGTCTCTCAAGGCACACAGGGG

>HYQZ1MD01A2EXD

CACAGTGCGTGGTCGGCGTCTCTCAAGGCACACAGGGG

>HYQZ1MD01BAQUR

CACAGTGCGTGGTCGCGTCTCTCAAGGCACACAGGGGAAAGG

>HYQZ1MD01ALOOY

CACAGTGCGTGGTCGGCGTCTCTCAAGGCACACAGGGGAAAGG

>HYQZ1MD01AT95E

ACAGTGCGTGGTCGGCGTCTCTCAAGGCACACAGGGG

>HYQZ1MD01AG5RV

CACAGTGCGTGGTCGGCTTCTCTCAAGGCACACAGGGGAAAGG

>HYQZ1MD01AFLXX

ACAGTGCGTGGTCGGCGTCTCTCAAGGCACACAGGGGG

>HYQZ1MD01C6P9J

CACAGTGCGTGGTCGCGTCTCTCAAGGCACACAGGGGAAAGG

>HYQZ1MD01C2KNP

CACAGTGCGTGTCGGCGTCTCTCAAGGCACACAGGGGAAAGG

>HYQZ1MD01CN8T7

CACAGTGCGTGGTCGGCGTCTCTCAAGGCACACAGGGGAAAGG

>HYQZ1MD01AN4SI

CACAGTGCGTGGTCGGCGTCTCTCAAGGCACACAGGGGAAAGG

>HYQZ1MD01BHZDS

CACAGTGCGTGGTCGGCGTCTCTCAAGGCACACAGGGG

>HYQZ1MD01AMOYX

CACAGTGCGTGGTCGGCGTCTCTCAAGGCACACAGGGGAAA

>HYQZ1MD01BIW1T

CACAGTGCGTGGTCGGCGTCTCTCAAGGCACACAGGGGAAA

>HYQZ1MD01AKTCL

CACAGTGCGTGGTCGGCGTCTCTCAAGGCACACAGGGG

>HYQZ1MD01A7Q9H

CACAGTGCGTGGTCGGCGTCTCTCAAGGCACACAGGGGAAAGG

>HYQZ1MD01DIQ61

CACAGTGCGTGGTCGGCGTCTCTCAAGGCACACAGGGGAAAGG

>HYQZ1MD01CI2RE

CACAGTGCGTGGTCGGCGTCTCTCAAGGCACACAGGGGAAAGG

>HYQZ1MD01DB6GK

CACAGTGCGTGGTCGGCGTCTCTCAAGGCACACAGGGGAAAGG

>HYQZ1MD01B2AFL

CACAGTGCGTGGTCGCGTCTCTCAAGGCACACAGGGGAAAG

>HYQZ1MD01AEN1M

CACAGTGCGTGTCGGCGTCTCTCAAGGCACACAGGGGAAAGG

>HYQZ1MD01A21BD

CACAGTGCGTGGTCGGCGTCTCTCAAGGCACACAGGGG

>HYQZ1MD01DPG9S

CACAGTGCGTGGTCGGCGTCTCTCAAGGCACACAGGGGAAAGG

>HYQZ1MD01BY6AC

CACAGTGCGTGGTCGGCGTCTCTCAAGGCACACAGGGGAAAGG

>HYQZ1MD01A3KNQ

CACAGTGCGTGGTCGGCGTCTCTCAAGGCACACAGGGG

>HYQZ1MD01BFWRN

CACAGTGCGTGGTCGGCGTCTCTCAAGGCACACAGGGGAAAGG

>HYQZ1MD01ALV8A

CACAGTGCGTGGTCGGCGTCTCTCAAGGCACACAGGGG

>HYQZ1MD01DEHB2

ACAGTGCGTGGTCGGCGTCTCTCAAGGCACACAGGGG

>HYQZ1MD01DNIJ3

CACAGTGCGTGGTCGGCGTCTCTCAAGGCACACAGGGG

>HYQZ1MD01ASJOB

CACAGTGCGTGGTCGGCGTCTCTCAAGCACACAGGGAAAGG

>HYQZ1MD01DTPLX

CACAGTGCGTGGTCGGCGTCTCTCAAGGCACACAGGGGAAAGG

>HYQZ1MD01DOZHP

CACAGTGCGTGGTCGGCGTCTCTCAAGGCACACAGGGG

>HYQZ1MD01DDWVX

CACAGTGCGTGGTCGGCGTCTCTCAAGGCACACAGGGGAAAGG

>HYQZ1MD01B6J1P

CACAGTGCGTGGTCGGCGTCTCTCAAGGCACACAGGGGAAAGG

>HYQZ1MD01DHNEJ

CACAGTGCGTGGTCGGCGTCTCTCAAGGCACACAGGGGAAAGG

>HYQZ1MD01CTFH1

CACAGTGCGTGGTCGGCGTCTCTCAAGGCACACAGGGGAAAGG

>HYQZ1MD01CBLRR

CACAGTGCGTGGTCGGCGTCTCTCAAGGCACACAGGGGAAAGG

>HYQZ1MD01BNW5Z

CACAGTGCGTGGTCGGCGTCTCTCAAGGCACACAGGGG

>HYQZ1MD01CSD0M

CAGTGCGTGGTCGGCGTCTCTCAAGGCACACAGGGGAAAGG

>HYQZ1MD01BMLQ4

CACAGTGCGTGGTCGCGTCTCTCAAGGCACACAGGGG

>HYQZ1MD01C72P5

CACAGTGCGTGGTCGGCGTCTCTCAAGGCACACAGGGG

>HYQZ1MD01B2M76

CACAGTGCGTGGTCGGCGTCTCTCAAGGCACACAGGGGAAAGG

>HYQZ1MD01CQALV

CACAGTGCGTGGTCGGCGTCTCTCAAGGCACACAGGGG

>HYQZ1MD01C041Y

CACAGTGCGTGGTCGGCGTCTCTCAAGGCACACAGGGG

>HYQZ1MD01BYU6M

CACAGTGCGTGGTCGCGTCTCTCAAGGCACACAGGGGAAAGG

>HYQZ1MD01AHZRJ

CACAGTGCGTGGTCGGCGTCTCTCAAGGCACACAGGGGAAAGG

>HYQZ1MD01C0JP8

CACAGTGCGTGGTCGGCGTCTCTCAAGGCACACAGGGGAAAGG

>HYQZ1MD01CICKQ

CACAGTGCGTGGTCGGCGTCTCTCAAGGCACACAGGGGAAAGG

>HYQZ1MD01BMKKT

CACAGTGCGTGGTCGGCGTCTCTCAAGGCACACAGGGGAAAGG

>HYQZ1MD01DJTPT

CACAGTGCGTGGTCGGCGTCTCTCAAGGCACACAGGGG

>HYQZ1MD01AYYH6

CACAGTGCGTGGTCGGCGTCTCTCAAGGCACACAGGGGAAAGG

>HYQZ1MD01B6WYA

ACAGTGCGTGGTCGGCGTCTCTCAAGGCACACAGGGG

>HYQZ1MD01DISPE

CACAGTGCGTGGTCGGCGTCTCTCAAGGCACACAGGGGAAAGG

>HYQZ1MD01DD579

CACAGTGCGTGGTCGGCGTCTCTCAAGCACACAGGGAAA

>HYQZ1MD01COA06

CACAGTGCGTGGTCGGCGTCTCTCAAGGCACACAGGGG

>HYQZ1MD01BJZTH

CACAGTGCGTGGTCGGCGTCTCTCAAGGCACACAGGGG

>HYQZ1MD01BI8LJ

CACAGTGCGTGGTCGGCGTCTCTCAAGGCACACAGGGGAAAGG

>HYQZ1MD01C4YJ9

CACAGTGCGTGGTCGGCGTCTCTCAAGGCACACAGGGGAAAGG

>HYQZ1MD01B26OG

CACAGTGCGTGGTCGGCGTCTCTCAAGGCACACAGGGGAAAGG

>HYQZ1MD01AAQGK

CACAGTGCGTGGTCGGCGTCTCTCAAGGCACACAGGGG

>HYQZ1MD01B2LHA

CACAGTGCGTGGTCGGCGTCTCTCAAGGCACACAGGGG

>HYQZ1MD01BVE1X

ACAGTGCGTGGTCGGCGTCTCTCAAGGCACACAGGGGG

>HYQZ1MD01CRMOF

CACAGTGCGTGGTCGGCGTCTCTCAAGGCACACAGGGG

>HYQZ1MD01BOTCT

CACAGTGCGTGGTCGCGTCTCTCAAGGCACACAGGGGAAAG

>HYQZ1MD01B1BTE

CACAGTGCGTGGTCGGCGTCTCTCAAGGCACACAGGGGAAAGG

>HYQZ1MD01CW6RG

CACAGTGCGTGGTCGGCGTCTCTCAAGGCACACAGGGG

>HYQZ1MD01BF1EF

CACAGTGCGTGGTCGGCGTCTCTCAAGGCACACAGGGGAAAGG

>HYQZ1MD01AONSS

CACAGTGCGTGGTCGGCGTCTCTCAAGGCACACAGGGGAAAGG

>HYQZ1MD01CRXI1

CACAGTGCGTGTCGCGTCTCTCAAGCACACAGGGGAAAGG

>HYQZ1MD01A5R2W

CACAGTGCGTGGTCGGCGTCTCTCAAGGCACACAGGGGAAAGG

>HYQZ1MD01BQGR8

CACAGTGCGTGGTCGGCGTCTCTCAAGGCACACAGGGGAAAGG

>HYQZ1MD01BO7FK

CACAGTGCGTGGTCGGCGTCTCTCAAGGCACACAGGGGAAAGG

>HYQZ1MD01BY6U9

CACAGTGCGTGGTCGGCGTCTCTCAAGGCACACAGGGG

>HYQZ1MD01B2KAX

CACAGTGCGTGGTCGGCGTCTCTCAAGGCACACAGGGGAAAGG

>HYQZ1MD01AT033

CACAGTGCGTGGTCGCGTCTCTCAAGGCACACAGGGG

>HYQZ1MD01A22K8

CACAGTGCGTGGTCGGCGTCTCTCAAGGCACACAGGGG

>HYQZ1MD01AQU6U

CACAGTGCGTGGTCGGCGTCTCTCAAGGCACACAGGGG

>HYQZ1MD01B1ATI

CACAGTGCGTGGTCGGCGTCTCTCAAGGCACACAGGGG

>HYQZ1MD01CTI5T

CACAGTGCGTGGTCGGCGTCTCTCAAGGCACACAGGGG

>HYQZ1MD01DASR3

CACAGTGCGTGGTCGGCGTCTCTCAAGGCACACAGGGGAAAGG

>HYQZ1MD01C5MMJ

CACAGTGCGTGGTCGGCGTCTCTCAAGGCACACAGGGGAAAG

>HYQZ1MD01AQ996

CACAGTGCGTGGTCGGCGTCTCTCAAGGCACACAGGGG

>HYQZ1MD01C91MG

CACAGTGCGTGGTCGGCGTCTCTCAAGGCACACAGGGGAAAGG

>HYQZ1MD01CXTD1

CACAGTGCGTGGTCGGCGTCTCTCAAGGCACACAGGGGAAAGG

>HYQZ1MD01A51N0

CACAGTGCGTGGTCGGCGTCTCTCAAGGCACACAGGGG

>HYQZ1MD01DHIA4

CACAGTGCGTGGTCGGCGTCTCTCAAGGCACACAGGGG

>HYQZ1MD01CDFQ6

CACAGTGCGTGGTCGGCGTCTCTCAAGGCACACAGGGGAAAGG

>HYQZ1MD01DL8IG

CACAGTGCGTGGTCGGCGTCTCTCAAGGCACACAGGGGAAAGG

>HYQZ1MD01AFKD3

CACAGTGCGTGGTCGGCGTCTCTCAAGGCACACAGGGGAAAGG

>HYQZ1MD01CPQWK

CACAGTGCGTGGTCGGCGTCTCTCAAGGCACACAGGGGAAAGG

>HYQZ1MD01AZEFW

CACAGTGCGTGGTCGGCGTCTCTCAAGGCACACAGGGGAAAGG

>HYQZ1MD01BTRJO

CACAGTGCGTGGTCGGCGTCTCTCAAGGCACACAGGGG

>HYQZ1MD01C38DX

CACAGTGCGTGGTCGGCGTCTCTCAAGGCACACAGGGGAAAGG

>HYQZ1MD01CUT2J

ACAGTGCGTGGTCGGCGTCTCTCAAGGCACACAGGGGAAAG

>HYQZ1MD01B36RU

CACAGTGCGTGGTCGGCGTCTCTCAAGGCACACAGGGGAAAGG

>HYQZ1MD01C13GG

CACAGTGCGTGGTCGCGTCTCTCAAGGCACACAGGGG

>HYQZ1MD01B7WD3

CACAGTGCGTGGTCGGCGTCTCTCAAGGCACACAGGGG

>HYQZ1MD01CCQSF

CACAGTGCGTGGTCGCGTCTCTCAAGGCACACAGGGGAAAG

>HYQZ1MD01B5E93

CACAGTGCGTGGTCGGCGTCTCTCAAGGCACACAGGGG

>HYQZ1MD01B2ISB

ACAGTGCGTGGTCGGCGTCTCTCAAGGCACACAGGGG

>HYQZ1MD01BJARZ

CACAGTGCGTGGTCGGCGTCTCTCAAGGCACACAGGGGTAAA

>HYQZ1MD01AWIKK

CAGTGCGTGGTCGGCGTCTCTCAAGGCACACAGGGGAAA

>HYQZ1MD01BPJ5P

CACAGTGCGTGTCGGCGTCTCTCAAGGCACACAGGGGAAA

>HYQZ1MD01AP6JJ

CACAGTGCGTGGTCGGCGTCTCTCAAGGCACACAGGGGAAAGG

>HYQZ1MD01B8SZ8

CACAGTGCGTGGTCGGCGTCTCTCAAGGCACACAGGGGAAAGG

>HYQZ1MD01CCPHS

CACAGTGCGTGGTCGGCGTCTCTCAAGGCACACAGGGG

>HYQZ1MD01C2TPE

CACAGTGCGTGTCGGCGTCTCTCAAGGCACACAGGGGAAAGG

>HYQZ1MD01ANL6B

CACAGTGCGTGGTCGGCGTCTCTCAAGGCACACAGGGGAAAGG

>HYQZ1MD01DGZQV

CACAGTGCGTGGTCGGCGTCTCTCAAGGCACACAGGGGAAAGG

>HYQZ1MD01AT3VL

CACAGTGCGTGGTCGGCGTCTCTCAAGGCACACAGGGG

>HYQZ1MD01CTSWO

CACAGTGCGTGGTCGGCGTCTCTCAAGGCACACAGGGGAAAGG

>HYQZ1MD01DVTPT

CACAGTGCGTGGTCGGCGTCTCTCAAGGCACACAGGGGAAA

>HYQZ1MD01BXS86

CACAGTGCGTGGTCGGCGTCTCTCAAGGCACACAGGGGAAAGG

>HYQZ1MD01DMHXA

CACAGTGCGTGGTCGGCGTCTCTCAAGCACACA

>HYQZ1MD01BHMMU

CACAGTGCGTGTCGGCGTCTCTCAAGGCACACAGGGG

>HYQZ1MD01AGKPW

CACAGTGCGTGGTCGGCGTCTCTCAAGGCACACAGGGGAAAGG

>HYQZ1MD01A0YM8

CACAGTGCGTGGTCGGCGTCTCTCAAGGCACACAGGGG

>HYQZ1MD01A2VA1

CACAGTGCGTGGTCGGCGTCTCTCAAGGCACACAGGGGAAAGG

>HYQZ1MD01BQY1C

CACAGTGCGTGGTCGGCGTCTCTCAAGGCACACAGGGGAAAGG

>HYQZ1MD01DSXXE

CACAGTGCGTGGTCGGCGTCTCTCAAGGCACACAGGGGTAAAGG

>HYQZ1MD01CI3CI

CACAGTGCGTGGTCGGCGTCTCTCAAGGCACACAGGGG

>HYQZ1MD01C286K

CACAGTGCGTGGTCGGCGTCTCTCAAGGCACACAGGGGAAAGG

>HYQZ1MD01AV8CD

CACAGTGCGTGGTCGCGTCTCTCAAGGCACACAGGGGAAAG

>HYQZ1MD01BXALY

CACAGTGCGTGGTCGGCGTCTCTCAAGGCACACAGGGG

>HYQZ1MD01CP9CR

CACAGTGCGTGGTCGGCGTCTCTCAAGGCACACAGGGGAAAGG

>HYQZ1MD01A2PSW

CACAGTGCGTGGTCGGCGTCTCTCAAGGCACACAGGGG

>HYQZ1MD01C0ZFQ

CACAGTGCGTGGTCGGCGTCTCTCAAGGCACACAGGGGAAAGG

>HYQZ1MD01DQ2QB

CACAGTGCGTGGTCGCGTCTCTCAAGGCACACAGGGGAAAGG

>HYQZ1MD01DQM58

CACAGTGCGTGGTCGGCGTCTCTCAAGGCACACAGGGGAAAGG

>HYQZ1MD01C6YE5

CACAGTGCGTGGTCGGCGTCTCTCAAGGCACACAGGGGAAAGG

>HYQZ1MD01A4KDG

CACAGTGCGTGGTCGGCGTCTCTCAAGGCACACAGGGG

>HYQZ1MD01BHZCA

CACAGTGCGTGGTCGGCGTCTCTCAAGGCACACAGGGG

>HYQZ1MD01DPF8P

ACAGTGCGTGGTCGGCGTCTCTCAAGGCACACAGGGGAAAGG

>HYQZ1MD01A34AC

CACAGTGCGTGGTCGGCGTCTCTCAAGGCACACAGGGG

>HYQZ1MD01DCCHX

CACAGTGCGTGGTCGGCGTCTCTCAAGGCACACAGGGGAAAGG

>HYQZ1MD01BV6Z1

CACAGTGCGTGGTCGGCGTCTCTCAAGGCACACAGGGG

>HYQZ1MD01DFAMG

CACAGTGCGTGGTCGGCGTCTCTCAGGCACACAGGGGAAAGG

>HYQZ1MD01CX9XY

CACAGTGCGTGGTCGGCGTCTCTCAAGGCACACAGGGGAAAGG

>HYQZ1MD01C7NIR

CACAGTGCGTGGTCGGCGTCTCTCAAGGCACACAGGGG

>HYQZ1MD01B62X9

CACAGTGCGTGGTCGGCGTCTCTCAAGGCACACAGGGGAAAGG

>HYQZ1MD01B5GDL

CACAGTGCGTGGTCGGCGTCTCTCAAGGCACACAGGGGAAA

>HYQZ1MD01AJO4E

ACAGTGCGTGGTCGGCGTCTCTCAAGGCACACAGGGGAAAGG

>HYQZ1MD01AA9M0

CACAGTGCGTGTCGGCGTCTCTCAAGGCACACAGGGG

>HYQZ1MD01AWDIR

CACAGTGCGTGGTCGGCGTCTCTCAAGGCACACAGGGG

>HYQZ1MD01DF7N6

CACAGTGCGTGGTCGGCGTCTCTCAAGGCACACAGGGGAAA

>HYQZ1MD01CFD0Q

CACAGTGCGTGGTCGGCGTCTCTCAAGGCACACAGGGGAAAGG

>HYQZ1MD01BXB8V

ACAGTGCGTGGTCGGCGTCTCTCAAGGCACACAGGGGGAAAGG

>HYQZ1MD01A2RHS

CACAGTGCGTGGTCGGCGTCTCTCAAGGCACACAGGGGAAAGG

>HYQZ1MD01BKI48

CACAGTGCGTGGTCGGCGTCTCTCAAGGCACACAGGGG

>HYQZ1MD01BVKIQ

CACAGTGCGTGGTCGGCGTCTCTCAAGGCACACAGGGG

>HYQZ1MD01B78JE

CACAGTGCGTGGTCGGCGTCTCTCAAGGCACACAGGGG

>HYQZ1MD01BDOS7

CACAGTGCGTGGTCGGCGTCTCTCAAGGCACACAGGGGAAAGG

>HYQZ1MD01BMNB3

CACAGTGCGTGGTCGGCGTCTCTCAAGGCACACAGGGGAAAGG

>HYQZ1MD01CQ30D

CACAGTGCGTGGTCGGCGTCTCTCAAGGCACACAGGGG

>HYQZ1MD01BWYM6

CACAGTGCGTGGTCGGCGTCTCTCAAGGCACACAGGGGAAAGG

>HYQZ1MD01AVMAU

CACAGTGCGTGGTCGGCGTCTCTCAAGGCACACAGGGGAAAGG

>HYQZ1MD01ABWXA

CACAGTGCGTGGTCGGCGTCTCTCAAGGCACACAGGGG

>HYQZ1MD01B6JXA

CACAGTGCGTGGTCGGCGTCTCTCAAGGCACACAGGGGAAAGG

>HYQZ1MD01BHIZZ

CACAGTGCGTGGTCGGCGTCTCTCAAGGCACACAGGGGAAAGG

>HYQZ1MD01CHV0W

CACAGTGCGTGGTCGGCGTCTCTCAAGGCACACAGGGGGTAAA

>HYQZ1MD01DO036

CACAGTGCGTGGTCGGCGTCTCTCAAGGCACACAGGGGAAAGG

>HYQZ1MD01DHCDY

CAGTGCGTGGTCGGCGTCTCTCAAGGCACACAGGGGAAAGG

>HYQZ1MD01BLJZA

CACAGTGCGTGGTCGGCGTCTCTCAAGGCACACAGGGGAAAGG

>HYQZ1MD01DM1XA

CACAGTGCGTGGTCGGCGTCTCTCAAGGCACACAGGGGAAAGG

>HYQZ1MD01APZEA

CACAGTGCGTGGTCGGCGTCTCTCAAGGCACACAGGGGAAAGG

>HYQZ1MD01BSTZG

CACAGTGCGTGGTCGGCGTCTCTCAAGGCACACAGGGGAAAGG

>HYQZ1MD01BG922

CACAGTGCGTGGTCGGCGTCTCTCAAGGCACACAGGGG

>HYQZ1MD01AATLZ

CACAGTGCGTGGTCGCGTCTCTCAAGGCACACAGGGGAAAGG

>HYQZ1MD01BVJQR

CACAGTGCGTGGTCGGCGTCTCTCAAGGCACACAGGGGAAAGG

>HYQZ1MD01A64QA

CACAGTGCGTGGTCGGCGTCTCTCAAGGCACACAGGGGAAAGG

>HYQZ1MD01DJ7UR

CACAGTGCGTGGTCGGCGTCTCTCAAGGCACACAGGGG

>HYQZ1MD01AD3Y5

CACAGTGCGTGGTCGGCGTCTCTCAAGGCACACAGGGG

>HYQZ1MD01DJQP3

CACAGTGCGTGGTCGGCGTCTCTCAAGGCACACAGGGGAAAGG

>HYQZ1MD01CIW4P

CACAGTGCGTGGTCGGCGTCTCTCAAGGCACACAGGGG

>HYQZ1MD01A6FEE

CACAGTGCGTGGTCGGCGTCTCTCAAGGCACACAGGGGAAAGG

>HYQZ1MD01BG13U

CACAGTGCGTGGTCGGCGTCTCTCAAGGCACACAGGGGAAAGG

>HYQZ1MD01A2E26

CACAGTGCGTGGTCGGCGTCTCTCAAGGCACACAGGGG

>HYQZ1MD01CW97X

CACAGTGCGTGGTCGGCGTCTCTCAAGGCACACAGGGGAAAGG

>HYQZ1MD01AWTXB

CACAGTGCGTGGTCGGCGTCTCTCAAGGCACACAGGGG

>HYQZ1MD01C29CT

CACAGTGCGTGGTCGGCGTCTCTCAAGGCACACAGGGGAAAGG

>HYQZ1MD01BA37V

CACAGTGCGTGGTCGGCGTCTCTCAAGGCACACAGGGG

>HYQZ1MD01DHR70

CACAGTGCGTGGTCGGCGTCTCTCAAGGCACACAGGGG

>HYQZ1MD01CEVBV

CACAGTGCGTGGTCGGCGTCTCTCAAGGCACACAGGGGAAAGG

>HYQZ1MD01B3UFM

CACAGTGCGTGGTCGGCGTCTCTCAAGGCACACAGGGGTAAA

>HYQZ1MD01DE3ZC

CACAGTGCGTGGTCGGCGTCTCTCAAGGCACACAGGGGAAAGG

>HYQZ1MD01BJAIJ

CACAGTGCGTGGTCGCGTCTCTCAAGGCACACAGGGGTAAA

>HYQZ1MD01DHLXC

CACAGTGCGTGGTCGGCGTCTCTCAAGGCACACAGGGGAAAGG

>HYQZ1MD01BGV2W

CACAGTGCGTGGTCGGCGTCTCTCAAGGCACACAGGGGAAAGG

>HYQZ1MD01DOH22

CACAGTGCGTGGTCGGCGTCTCTCAAGGCACACAGGGGAAAGG

>HYQZ1MD01C1V67

CACAGTGCGTGGTCGCGTCTCTCAAGGCACACAGGGGAAAGG

>HYQZ1MD01B6S5C

CACAGTGCGTGGTCGCGTCTCTCAAGGCACACAGGGG

>HYQZ1MD01CAUP4

CACAGTGCGTGGTCGGCGTCTCTCAAGGCACACAGGGG

>HYQZ1MD01C8NNX

CACAGTGCGTGGTCGGCGTCTCTCAAGGCACACAGGGG

>HYQZ1MD01BDQAV

CACAGTGCGTGGTCGGCGTCTCTCAAGGCACACAGGGGAAAGG

>HYQZ1MD01A7806

CACAGTGCGTGGTCGGCGTCTCTCAAGGCACACAGGGGAAAGG

>HYQZ1MD01BEC3Q

CACAGTGCGTGGTCGCGTCTCTCAAGGCACACAGGGGAAAGG

>HYQZ1MD01DVVSJ

CACAGTGCGTGGTCGGCGTCTCTCAAGGCACACAGGGGAAAGG

>HYQZ1MD01CSFCU

CACAGTGCGTGGTCGGCGTCTCTCAAGGCACACAGGGG

>HYQZ1MD01DM52K

ACAGTGCGTGGTCGGCGTCTCTCAAGGCACACAGGGG

>HYQZ1MD01AHL98

CACAGTGCGTGGTCGCGTCTCTCAAGGCACACAGGGGAAAGG

>HYQZ1MD01B4B5G

CACAGTGCGTGGTCGGCGTCTCTCAAGGCACACAGGGGAAAGG

>HYQZ1MD01AX8VB

CACAGTGCGTGGTCGGCGTCTCTCAAGGCACACAGGGGAAAGG

>HYQZ1MD01CBLFJ

CACAGTGCGTGGTCGGCGTCTCTCAAGGCACACAGGGGAAAG

>HYQZ1MD01AEV1B

CACAGTGCGTGGTCGGCGTCTCTCAAGGCACACAGGGG

>HYQZ1MD01AVPA8

CACAGTGCGTGGTCGGCGTCTCTCAAGGCACACAGGGG

>HYQZ1MD01BH115

CACAGTGCGTGGTCGGCGTCTCTCAAGGCACACAGGGG

>HYQZ1MD01CTMNM

CACAGTGCGTGGTCGGCGTCTCTCAGGCACACAGGGGAAAGG

>HYQZ1MD01AL0TO

CACAGTGCGTGGTCGGCGTCTCTCAAGGCACACAGGGG

>HYQZ1MD01BI86U

CACAGTGCGTGGTCGGCGTCTCTCAAGGCACACAGGGGAAAGG

>HYQZ1MD01DKIUL

ACAGTGCGTGGTCGGCGTCTCTCAAGGCACACAGGGGAAA

>HYQZ1MD01DJZ9R

ACAGTGCGTGGTCGGCGTCTCTCAAGGCACACAGGGGAAA

>HYQZ1MD01AV5DK

CACAGTGCGTGGTCGGCGTCTCTCAAGGCACACAGGGGAAAGG

>HYQZ1MD01C67AX

CACAGTGCGTGGTCGGCGTCTCTCAAGGCACACAGGGG

>HYQZ1MD01CZ9YY

CACAGTGCGTGGTCGGCGTCTCTCAAGGCACACAGGGGAAAGG

>HYQZ1MD01ALI1G

CACAGTGCGTGGTCGGCGTCTCTCAAGGCACACAGGGGAAAGG

>HYQZ1MD01C8PZ9

CACAGTGCGTGGTCGGCGTCTCTCAAGGCACACAGGGGAAAGG

>HYQZ1MD01AH3MM

CACAGTGCGTGGTCGGCGTCTCTCAAGGCACACAGGGGAAAGG

>HYQZ1MD01C8QM7

CACAGTGCGTGGTCGGCGTCTCTCAAGGCACACAGGGG

>HYQZ1MD01CE3PE

CACAGTGCGTGGTCGGCGTCTCTCAAGGCACACAGGGG

>HYQZ1MD01DE6YO

CACAGTGCGTGGTCGGCGTCTCTCAAGGCACACAGGGGAAAGG

>HYQZ1MD01BVZLT

CACAGTGCGTGGTCGGCGTCTCTCAAGGCACACAGGGGAAAGG

>HYQZ1MD01C9SRY

CACAGTGCGTGGTCGGCGTCTCTCAAGGCACACAGGGG

>HYQZ1MD01CKS7G

CACAGTGCGTGGTCGCGTCTCTCAAGGCACACAGGGGAAAGG

>HYQZ1MD01ANQ4T

CACAGTGCGTGGTCGGCGTCTCTCAAGGCACACAGGGG

>HYQZ1MD01BDI16

CACAGTGCGTGGTCGGCGTCTCTCAAGGCACACAGGGGAAAGG

>HYQZ1MD01BCGFV

CACAGTGCGTGGTCGGCGTCTCTCAAGGCACACAGGGGAAA

>HYQZ1MD01C0MFG

CACAGTGCGTGGTCGGCGTCTCTCAAGGCACACAGGGGAAA

>HYQZ1MD01CELGO

CACAGTGCGTGGTCGGCGTCTCTCAAGGCACACAGGGGAAAGG

>HYQZ1MD01CS4OV

CACAGTGCGTGGTCGGCGTCTCTCAAGGCACACAGGGGAAAGG

>HYQZ1MD01BITTU

CACAGTGCGTGGTCGGCGTCTCTCAAGGCACACAGGGGAAAGG

>HYQZ1MD01C2QHY

CACAGTGCGTGGTCGGCGTCTCTCAAGGCACACAGGGG

>HYQZ1MD01AKXAP

CACAGTGCGTGGTCGGCGTCTCTCAAGGCACACAGGGGAAAGG

>HYQZ1MD01CPHA0

CACAGTGCGTGGTCGGCGTCTCTCAAGGCACACAGGGGAAAGG

>HYQZ1MD01DR1HC

CACAGTGCGTGGTCGGCGTCTCTCAAGGCACACAGGGG

>HYQZ1MD01BX95H

CACAGTGCGTGGTCGGCGTCTCTCAAGGCACACAGGGGAAAGG

>HYQZ1MD01A18WM

CACAGTGCGTGGTCGGCGTCTCTCAAGGCACACAGGGGAAAGG

>HYQZ1MD01A0CU1

CACAGTGCGTGGTCGGCGTCTCTCAAGGCACACAGGGG

>HYQZ1MD01CTMC3

CACAGTGCGTGGTCGGCGTCTCTCAAGGCACACAGGGGAAAGG

>HYQZ1MD01CH5TS

CACAGTGCGTGGTCGGCGTCTCTCAAGGCACACAGGGG

>HYQZ1MD01BYGLG

CACAGTGCGTGGTCGGCGTCTCTCAAGGCACACAGGGG

>HYQZ1MD01CL5SS

CACAGTGCGTGGTCGCGTCTCTCAAGGCACACAGGGGAAAG

>HYQZ1MD01DCNYN

CACAGTGCGTGGTCGGCGTCTCTCAAGGCACACAGGGGAAAGG

>HYQZ1MD01A93R1

CACAGTGCGTGGTCGGCGTCTCTCAAGGCACACAGGGG

>HYQZ1MD01BK996

CACAGTGCGTGGTCGCGTCTCTCAAGGCACACAGGGGAAAG

>HYQZ1MD01DQ34C

CACAGTGCGTGGTCGGCGTCTCTCAAGGCACACAGGGGAAAGG

>HYQZ1MD01BCV75

CACAGTGCGTGGTCGGCGTCTCTCAAGGCACACAGGGG

>HYQZ1MD01C39BQ

CACAGTGCGTGGTCGGCGTCTCTCAAGGCACACAGGGG

>HYQZ1MD01DPQAJ

CACAGTGCGTGGTCGGCGTCTCTCAAGGCACACAGGGGAAAGG

>HYQZ1MD01BMPSR

CACAGTGCGTGGTCGGCGTCTCTCAAGGCACACAGGGGAAAGG

>HYQZ1MD01COTQA

CACAGTGCGTGGTCGGCGTCTCTCAAGGCACACAGGGG

>HYQZ1MD01A1CZB

CACAGTGCGTGGTCGGCGTCTCTCAAGGCACACAGGGGAAAGG

>HYQZ1MD01AIJDY

CACAGTGCGTGGTCGGCGTCTCTCAAGGCACACAGGGGAAAGG

>HYQZ1MD01CLFIS

CACAGTCGTGGTCGGCGTCTCTCAAGGCACACAGGGGAAAGG

>HYQZ1MD01A06CY

CACAGTGCGTGGTCGGCGTCTCTCAAGGCACACAGGGGAAAGG

>HYQZ1MD01BDK0U

CACAGTGCGTGGTCGGCGTCTCTCAAGGCACACAGGGGAAAGG

>HYQZ1MD01DPWNN

CACAGTGCGTGGTCGCGTCTCTCAAGGCACACAGGGGAAAG

>HYQZ1MD01BHHFC

CACAGTGCGTGTCGGCGTCTCTCAAGGCACACAGGGGAAAGG

>HYQZ1MD01DU5BV

CACAGTGCGTGTCCGCGTCTCTCAAGGCACACAGGGG

>HYQZ1MD01DCVET

CACAGTGCGTGGTCGGCGTCTCTCAAGGCACACAGGGGTAAAGG

>HYQZ1MD01C9V1H

CACAGTGCGTGGTCGGCGTCTCTCAAGGCACACAGGGG

>HYQZ1MD01A1HZQ

CACAGTGCGTGGTCGGCGTCTCTCAAGGCACACAGGGGTAAAGG

>HYQZ1MD01CDJDC

CACAGTGCGTGGTCGGCGTCTCTCAAGGCACACAGGGGAAAGG

>HYQZ1MD01CN85X

CACAGTGCGTGGTCGGCGTCTCTCAAGGCACACAGGGGAAAGG

>HYQZ1MD01DHXE9

CACAGTGCGTGGTCGGCGTCTCTCAAGGCACACAGGGGAAAGG

>HYQZ1MD01BPNS7

CACAGTGCGTGGTCGGCGTCTCTCAAGGCACACAGGGGAAAGG

>HYQZ1MD01DBCKR

CACAGTGCGTGGTCGGCGTCTCTCAAGGCACACAGGGGG

>HYQZ1MD01CLP6S

CACAGTGCGTGTCGGCGTCTCTCAAGCACACAGGG

>HYQZ1MD01CKZAK

CACAGTGCGTGGTCGGCGTCTCTCAAGGCACACAGGGGAAAGG

>HYQZ1MD01DRTYJ

CACAGTGCGTGGTCGCGTCTCTCAAGGCACACAGGGGAAAG

>HYQZ1MD01C60QN

ACAGTGCGTGTCGGCGTCTCTCAAGGCACACAGGGGAAAG

>HYQZ1MD01AIGI4

CACAGTGCGTGGTCGGCGTCTCTCAAGGCACACAGGGG

>HYQZ1MD01AR1GZ

CACAGTGCGTGGTCGGCGTCTCTCAAGGCACACAGGGG

>HYQZ1MD01BNUU3

CACAGTGCGTGGTCGGCGTCTCTCAAGGCACACAGGGGAAA

>HYQZ1MD01DI5ZJ

CACAGTGCGTGGTCGGCGTCTCTCAAGGCACACAGGGGAAAGG

>HYQZ1MD01DVIDQ

CACAGTGCGTGGTCGCGTCTCTCAAGGCACACAGGGG

>HYQZ1MD01A7IGM

CACAGTGCGTGGTCGGCGTCTCTCAAGGCACACAGGGGAAAGG

>HYQZ1MD01DI6NR

CACAGTGCGTGGTCGGCGTCTCTCAAGGCACACAGGGG

>HYQZ1MD01A7Q24

CACAGTGCGTGGTCGGCGTCTCTCAAGGCACACAGGGGAAAGG

>HYQZ1MD01BZZ37

CACAGTGCGTGGTCGGCGTCTCTCAAGGCACACAGGGGAAAGG

>HYQZ1MD01DBEYI

CACAGTGCGTGGTCGGCGTCTCTCAAGGCACACAGGGGAAAGG

>HYQZ1MD01CZYY8

CACAGTGCGTGGTCGGCGTCTCTCAAGGCACACAGGGG

>HYQZ1MD01C5EBZ

CACAGTGCGTGGTCGGCGTCTCTCAAGGCACACAGGGG

>HYQZ1MD01A8WQS

CACAGTGCGTGGTCGGCGTCTCTCAAGGCACACAGGGGAAA

>HYQZ1MD01DBUIB

CACAGTGCGTGGTCGGCGTCTCTCAAGGCACACAGGGG

>HYQZ1MD01A3K8B

CACAGTGCGTGGTCGGCGTCTCTCAAGGCACACAGGGG

>HYQZ1MD01C1821

CACAGTGCGTGGTCGGCGTCTCTCAAGGCACACAGGGG

>HYQZ1MD01DVWGV

CACAGTGCGTGGTCGGCGTCTCTCAAGGCACACAGGGG

>HYQZ1MD01CEP0W

CACAGTGCGTGGTCGGCGTCTCTCAAGGCACACAGGGGAAA

>HYQZ1MD01DM3LV

CACAGTGCGTGGTCGGCGTCTCTCAAGGCACACAGGGGAAAGG

>HYQZ1MD01AM9U1

CACAGTGCGTGGTCGGCGTCTCTCAAGGCACACAGGGG

>HYQZ1MD01B7QWT

CACAGTGCGTGGTCGGCGTCTCTCAAGGCACACAGGGG

>HYQZ1MD01ATOA7

CACAGTGCGTGGTCGGCGTCTCTCAAGGCACACAGGGGAAAGG

>HYQZ1MD01AXQZ1

CACAGTGCGTGGTCGGCGTCTCTCAAGGCACACAGGGG

>HYQZ1MD01AE2RH

CACAGTGCGTGTCGGCGTCTCTCAAGGCACACAGGGG

>HYQZ1MD01C4TZG

CACAGTGCGTGGTCGGCGTCTCTCAAGGCACACAGGGGAAAGG

>HYQZ1MD01B78H8

CACAGTGCGTGGTCGGCGTCTCTCAAGGCACACAGGGGAAAGG

>HYQZ1MD01BMTUL

CACAGTGCGTGGTCGGCGTCTCTCAAGGCACACAGGGG

>HYQZ1MD01ANIQW

CACAGTGCGTGGTCGGCGTCTCTCAAGGCACACAGGGG

>HYQZ1MD01CMIG3

CACAGTGCGTGGTCGGCGTCTCTCAAGGCACACAGGGGAAAGG

>HYQZ1MD01CA4DK

CACAGTGCGTGGTCGGCGTCTCTCAAGGCACACAGGGGAAAGG

>HYQZ1MD01AUZ99

CACAGTGCGTGGTCGCGTCTCTCAAGGCACACAGGGGAAAG

>HYQZ1MD01DROHQ

CACAGTGCGTGGTCGGCGTCTCTCAAGGCACACAGGGGAAAG

>HYQZ1MD01DML1K

CACAGTGCGTGGTCGGCGTCTCTCAAGGCACACAGGGGAAAGG

>HYQZ1MD01DVDZW

CACAGTGCGTGGTCGCGTCTCTCAAGCACACAGGGG

>HYQZ1MD01CX5QN

CACAGTGCGTGGTCGGCGTCTCTCAAGGCACACAGGGGAAAGG

>HYQZ1MD01CSUE9

CACAGTGCGTGGCGGCGTCTCTCAAGGCACACAGGGGAAA

>HYQZ1MD01C6DFV

CACAGTGCGTGGTCGGCGTCTCTCAAGGCACACAGGGG

>HYQZ1MD01DVS99

CACAGTGCGTGGTCGGCGTCTCTCAAGGCACACAGGGGAAAGG

>HYQZ1MD01AN7MB

CACAGTGCGTGGTCGGCGTCTCTCAAGGCACACAGGGG

>HYQZ1MD01B3BLH

CACAGTGCGTGGTCGGCGTCTCTCAAGGCACACAGGGG

>HYQZ1MD01BRDWD

CACAGTGCGTGGTCGGCGTCTCTCAAGGCACACAGGGGAAA

>HYQZ1MD01CPECH

CACAGTGCGTGGTCGGCGTCTCTCAAGGCACACAGGGGAAAGG

>HYQZ1MD01BVEUO

CACAGTGCGTGGTCGGCGTCTCTCAAGGCACACAGGGGTAAA

>HYQZ1MD01CBJPW

CACAGTGCGTGGTCGGCGTCTCTCAAGGCACACAGGGG

>HYQZ1MD01BJNT5

CACAGTGCGTGGTCGGCGTCTCTCAAGGCACACAGGGG

>HYQZ1MD01ANM2N

CACAGTGCGTGGTCGGCGTCTCTCAAGGCACACAGGGGAAAGG

>HYQZ1MD01CCD2J

CACAGTGCGTGGTCGGCGTCTCTCAAGGCACACAGGGGAAAGG

>HYQZ1MD01DT9AW

CACAGTGCGTGGTCGGCGTCTCTCAAGGCACACAGGGG

>HYQZ1MD01B94MQ

CACAGTGCGTGGTCGGCGTCTCTCAAGGCACACAGGGGAAAGG

>HYQZ1MD01AJZXJ

CACAGTGCGTGGTCGGCGTCTCTCAAGGCACACAGGGGAAAG

>HYQZ1MD01BAA4D

CACAGTGCGTGGTCGGCGTCTCTCAAGGCACACAGGGGG

>HYQZ1MD01ANECL

CACAGTGCGTGGTCGGCGTCTCTCAAGGCACACAGGGGAAA

>HYQZ1MD01AFQQQ

CACAGTGCGTGGTCGGCGTCTCTCAAGGCACACAGGGG

>HYQZ1MD01AS0T3

CACAGTGCGTGGTCGGCGTCTCTCAAGGCACACAGGGG

>HYQZ1MD01A4D5E

CACAGTGCGTGGTCGGCGTCTCTCAAGGCACACAGGGGAAAGG

>HYQZ1MD01DSL7D

CACAGTGCGTGGTCGGCGTCTCTCAAGGCACACAGGGG

>HYQZ1MD01B5WFP

CACAGTGCGTGGTCGGCGTCTCTCAAGGCACACAGGGGAAAGG

>HYQZ1MD01DHVX8

CACAGTGCGTGGTCGGCGTCTCTCAAGGCACACAGGGGAAAGG

>HYQZ1MD01BWNEO

CACAGTGCGTGGTCGGCGTCTCTCAAGGCACACAGGGGAAAGG

>HYQZ1MD01AMQIZ

CACAGTGCGTGGTCGGCGTCTCTCAAGGCACACAGGGGAAAGG

>HYQZ1MD01DG2Y5

CACAGTGCGTGGTCGGCGTCTCTCAAGGCACACAGGGG

>HYQZ1MD01CW4QV

CACAGTGCGTGGTCGGCGTCTCTCAAGGCACACAGGGG

>HYQZ1MD01AA6CZ

CACAGTGCGTGGTCGGCGTCTCTCAAGGCACACAGGGGAAA

>HYQZ1MD01C9X5M

CACAGTGCGTGGTCGGCGTCTCTCAAGGCACACAGGGG

>HYQZ1MD01CRH11

CACAGTGCGTGGTCGGCGTCTCTCAAGGCACACAGGGGAAAGG

>HYQZ1MD01CSL2T

CACAGTGCGTGGTCGGCGTCTCTCAAGGCACACAGGGG

>HYQZ1MD01C81RN

CACAGTGCGTGGTCGCGTCTCTCAAGGCACACAGGGGAAAGG

>HYQZ1MD01B7RBW

CACAGTGCGTGGTCGGCGTCTCTCAAGGCACACAGGGGAAAGG

>HYQZ1MD01DJFPO

CACAGTGCGTGGTCGGCGTCTCTCAAGGCACACAGGGG

>HYQZ1MD01BMRHJ

CACAGTGCGTGGTCGGCGTCTCTCAAGGCACACAGGGG

>HYQZ1MD01CTL0U

CACAGTGCGTGGTCGGCGTCTCTCAAGGCACACAGGGGAAAGG

>HYQZ1MD01AH66K

CACAGTGCGTGGTCGGCGTCTCTCAAGGCACACAGGGG

>HYQZ1MD01B3EVC

CACAGTGCGTGGTCGGCGTCTCTCAAGGCACACAGGGGAAAGG

>HYQZ1MD01BRYOC

CACAGTGCGTGGTCGGCGTCTCTCAAGGCACACAGGGG

>HYQZ1MD01DELOC

CACAGTGCGTGGTCGGCGTCTCTCAAGGCACACAGGGG

>HYQZ1MD01CMQ44

CACAGTGCGTGGTCGGCGTCTCTCAAGGCACACAGGGGAAAGG

>HYQZ1MD01A9E70

CACAGTGCGTGTCGCGTCTCTCAAGGCACACAGGGG

>HYQZ1MD01C09W5

ACAGTGCGTGGTCGGCGTCTCTCAAGGCACACAGGGGAAAGG

>HYQZ1MD01BUT1X

CACAGTGCGTGTCGGCGTCTCTCAAGGCACACAGGGGAAAGG

>HYQZ1MD01ADPVN

CACAGTGCGTGGTCGGCGTCTCTCAAGGCACACAGGGGAAAGG

>HYQZ1MD01A4LRM

CACAGTGCGTGGTCGGCGTCTCTCAAGGCACACAGGGG

>HYQZ1MD01AFRNJ

CACAGTGCGTGTCGGCGTCTCTCAAGGCACACAGGGG

>HYQZ1MD01C2J0B

CACAGTGCGTGGTCGGCGTCTCTCAAGGCACACAGGGGAAAGG

>HYQZ1MD01AHSNB

CACAGTGCGTGGTCGGCGTCTCTCAAGGCACACAGGGGAAA

>HYQZ1MD01CSO9Y

ACAGTGCGTGGTCGGCGTCTCTCAAGGCACACAGGGGAAAGG

>HYQZ1MD01B2SFP

CACAGTGCGTGGTCGGCGTCTCTCAAGGCACACAGGGGAAAGG

>HYQZ1MD01BK13A

CACAGTGCGTGGTCGGCGTCTCTCAAGGCACACAGGGG

>HYQZ1MD01C3FFC

CACAGTGCGTGGTCGGCGTCTCTCAAGGCACACAGGGGAAAGG

>HYQZ1MD01BH5VZ

CACAGTGCGTGGTCGGCGTCTCTCAAGGCACACAGGGG

>HYQZ1MD01DEEGO

CACAGTGCGTGGTCGGCGTCTCTCAAGGCACACAGGGGAAAGG

>HYQZ1MD01DN9TJ

CACAGTGCGTGGTCGGCGTCTCTCAAGGCACACAGGGG

>HYQZ1MD01CMVY5

CACAGTGCGTGGTCGGCGTCTCTCAAGGCACACAGGGG

>HYQZ1MD01DC57A

CACAGTGCGTGGTCGGCGTCTCTCAAGGCACACAGGGGAAAGG

>HYQZ1MD01B0MCM

CACAGTGCGTGGTCGGCGTCTCTCAAGGCACACAGGGGAAA

>HYQZ1MD01ARGE7

CACAGTGCGTGGTCGGCGTCTCTCAAGGCACACAGGGGAAAGG

>HYQZ1MD01ARC99

CACAGTGCGTGGTCGGCGTCTCTCAAGGCACACAGGGGAAAGG

>HYQZ1MD01BI1W8

CACAGTGCGTGGTCGGCGTCTCTCAAGGCACACAGGGGAAAGG

>HYQZ1MD01BI922

CACAGTGCGTGGTCGGCGTCTCTCAAGGCACACAGGGGAAA

>HYQZ1MD01AJQG6

CACAGTGCGTGGTCGGCGTCTCTCAAGGCACACAGGGG

>HYQZ1MD01BX2TQ

CACAGTGCGTGGTCGGCGTCTCTCAAGGCACACAGGGGAAAGG

>HYQZ1MD01B3MLK

CACAGTGCGTGGTCGGCGTCTCTCAAGGCACACAGGGGAAAGG

>HYQZ1MD01CH8E4

CACAGTGCGTGGTCGGCGTCTCTCAAGGCACACAGGGGG

>HYQZ1MD01BO47K

CACAGTGCGTGGTCGGCGTCTCTCAAGGCACACAGGGG

>HYQZ1MD01CYE84

CACAGTGCGTGGTCGGCGTCTCTCAAGGCACACAGGGGAAAGG

>HYQZ1MD01A8HIB

CACAGTGCGTGGTCGGCGTCTCTCAAGGCACACAGGGGAAAGG

>HYQZ1MD01A2DRR

CACAGTGCGTGGTCGGCGTCTCTCAAGGCACACAGGGGAAAGG

>HYQZ1MD01BE8TU

CACAGTGCGTGGTCGGCGTCTCTCAAGGCACACAGGGG

>HYQZ1MD01A18K4

CACAGTGCGTGGTCGGCGTCTCTCAAGGCACACAGGGGAAAGG

>HYQZ1MD01CRCOF

CACAGTGCGTGGTCGGCGTCTCTCAAGGCACACAGGGG

>HYQZ1MD01BZZWP

CACAGTGCGTGGTCGGCGTCTCTCAAGGCACACAGGGG

>HYQZ1MD01A0ZDW

CACAGTGCGTGGTCGGCGTCTCTCAAGGCACACAGGGGAAAGG

>HYQZ1MD01BNA80

CACAGTGCGTGGTCGGCGTCTCTCAAGGCACACAGGGGAAAGG

>HYQZ1MD01AVWCX

CACAGTGCGTGGTCGGCGTCTCTCAAGGCACACAGGGGAAAGG

>HYQZ1MD01CY1JC

CACAGTGCGTGGTCGGCGTCTCTCAAGGCACACAGGGGAAAGG

>HYQZ1MD01BOLWJ

CACAGTGCGTGGTCGGCGTCTCTCAAGGCACACAGGGGAAAGG

>HYQZ1MD01CXLPE

CACAGTGCGTGGTCGGCGTCTCTCAAGGCACACAGGGG

>HYQZ1MD01DVS91

CACAGTGCGTGGTCGGCGTCTCTCAAGGCACACAGGGG

>HYQZ1MD01B60IV

CACAGTGCGTGGTCGGCGTCTCTCAAGGCACACAGGGG

>HYQZ1MD01CYFES

CACAGTGCGTGGTCGGCGTCTCTCAAGGCACACAGGGGAAAGG

>HYQZ1MD01ARJSS

CACAGTGCGTGGTCGGCGTCTCTCAAGGCACACAGGGGAAAGG

>HYQZ1MD01DMGWW

CACAGTGCGTGGTCGGCGTCTCTCAAGCACACAGGGAAAGG

>HYQZ1MD01DB3PC

CACAGTGCGTGGTCGGCGTCTCTCAAGGCACACAGGGGAAAGG

>HYQZ1MD01CHCUU

ACAGTGCGTGGTCGGCGTCTCTCAAGGCACACAGGGG

>HYQZ1MD01ADRLM

CACAGTGCGTGGTCGGCGTCTCTCAAGGCACACAGGGGG

>HYQZ1MD01CIUXO

CACAGTGCGTGGTCGGCGTCTCTCAAGGCACACAGGGG

>HYQZ1MD01AO3MZ

CACAGTGCGTGGTCGGCGTCTCTCAAGGCACACAGGGG

>HYQZ1MD01CULO9

CACAGTGCGTGGTCGGCGTCTCTCAAGGCACACAGGGG

>HYQZ1MD01A021G

CACAGTGCGTGTCGGCGTCTCTCAAGCACACAGGGGAAA

>HYQZ1MD01AY67X

CACAGTGCGTGTCGCGTCTCTCAAGCACACAGGGGAAAGG

>HYQZ1MD01CDRW1

CACAGTGCGTGGTCGGCGTCTCTCAAGGCACACAGGGG

>HYQZ1MD01BYZB0

CACAGTGCGTGGTCGGCGTCTCTCAAGGCACACAGGGG

>HYQZ1MD01C9KE4

CACAGTGCGTGGTCGGCGTCTCTCAAAGGCACACAGGGGGAAAAGG

>HYQZ1MD01AV9Y9

CACAGTGCGTGGTCGGCGTCTCTCAAGGCACACAGGGG

>HYQZ1MD01BSDQR

ACAGTGCGTGGTCGGCGTCTCTCAAGGCACACAGGGGAAAGG

>HYQZ1MD01C8Y92

CACAGTGCGTGGTCGGCGTCTCTCAAGGCACACAGGGG

>HYQZ1MD01CJWZI

CACAGTGCGTGGTCGGCGTCTCTCAAGGCACACAGGGG

>HYQZ1MD01CTR8D

CACAGTGCGTGGTCGGCGTCTCTCAAGGCACACAGGGGAAAGG

>HYQZ1MD01AA9CJ

CACAGTGCGTGGTCGGCGTCTCTCAAGGCACACAGGGG

>HYQZ1MD01CKZ0S

CACAGTGCGTGGTCGGCGTCTCTCAAGGCACACAGGGG

>HYQZ1MD01AO5NZ

CACAGTGCGTGGTCGGCGTCTCTCAAGGCACACAGGGGAAAGG

>HYQZ1MD01C4BFQ

CACAGTGCGTGGTCGGCGTCTCTCAAGGCACACAGGGGAAAGG

>HYQZ1MD01AW2S2

CACAGTGCGTGGTCGGCGTCTCTCAAGGCACACAGGGG

>HYQZ1MD01CHIWN

CACAGTGCGTGTCGGCGTCTCTCAAGGCACACAGGGG

>HYQZ1MD01DJM89

CACAGTGCGTGGTCGGCGTCTCTCAAGGCACACAGGGGTAAA

>HYQZ1MD01CVDV5

CACAGTGCGTGGTCGGCGTCTCTCAAGGCACACAGGGGAAAGG

>HYQZ1MD01BFJ1R

CACAGTGCGTGGTCGGCGTCTCTCAAGGCACACAGGGG

>HYQZ1MD01BAD8U

CACAGTGCGTGGTCGGCGTCTCTCAAGGCACACAGGGGAAAGG

>HYQZ1MD01ATG55

ACAGTGCGTGGTCGGCGTCTCTCAAGGCACACAGGGGAAAGG

>HYQZ1MD01ASIEA

CACAGTGCGTGGTCGGCGTCTCTCAAGGCACACAGGGGAAA

>HYQZ1MD01BSN4Q

CACAGTGCGTGGTCGCGTCTCTCAAGGCACACAGGGGAAAGG

>HYQZ1MD01CCWMI

CACAGTGCGTGGTCGGCGTCTCTCAAGGCACACAGGGGAAAGG

>HYQZ1MD01BF7AU

CACAGTGCGTGGTCGGCGTCTCTCAAGGCACACAGGGGAAAG

>HYQZ1MD01A2IRM

CACAGTGCGTGGTCGGCGTCTCTCAAGGCACACAGGGG

>HYQZ1MD01CJ4U4

CACAGTGCGTGGTCGGCGTCTCTCAAGGCACACAGGGGG

>HYQZ1MD01C215D

CACAGTGCGTGTCGCGTCTCTCAAGCACACAGGGG

>HYQZ1MD01BHDH3

CACAGTGCGTGTCGCGTCTCTCAAGGCACACAGGGG

>HYQZ1MD01CYF88

CACAGTGCGTGGTCGGCGTCTCTCAAGGCACACAGGGG

>HYQZ1MD01CCT3Q

CACAGTGCGTGGTCGCGTCTCTCAAGGCACACAGGGG

>HYQZ1MD01B0ER3

CACAGTGCGTGGTCGGCGTCTCTCAAGGCACACAGGGGAAAGG

>HYQZ1MD01C6Z7Y

CACAGTGCGTGGTCGGCGTCTCTCAAGGCACACAGGGG

>HYQZ1MD01BVUU2

CACAGTGCGTGGTCGGCGTCTCTCAAGGCACACAGGGG

>HYQZ1MD01A7L1V

CACAGTGCGTGGTCGGCGTCTCTCAAGGCACACAGGGG

>HYQZ1MD01B72R5

CACAGTGCGTGGTCGGCGTCTCTCAAGGCACACAGGGG

>HYQZ1MD01C5684

CACAGTGCGTGGTCGGCGTCTCTCAAGGCACACAGGGG

>HYQZ1MD01BNBVJ

CACAGTGCGTGGTCGGCGTCTCTCAAGGCACACAGGGGG

>HYQZ1MD01C4FRH

CACAGTGCGTGGTCGGCGTCTCTCAAGGCACACAGGGGAAA

>HYQZ1MD01BTE21

CACAGTGCGTGGTCGCGTCTCTCAAGGCACACAGGGGAAAGG

>HYQZ1MD01APSUB

CACAGTGCGTGGTCGGCGTCTCTCAAGGCACACAGGGG

>HYQZ1MD01CJ1Q2

CACAGTGCGTGGTCGGCGTCTCTCAAGGCACACAGGGG

>HYQZ1MD01AKXWZ

CACAGTGCGTGGTCGGCGTCTCTCAAGGCACACAGGGG

>HYQZ1MD01BAFXE

CACAGTGCGTGGTCGGCGTCTCTCAAGGCACACAGGGG

>HYQZ1MD01B1KDB

ACAGTGCGTGGTCGGCGTCTCTCAAGGCACACAGGGG

>HYQZ1MD01AKNWG

CACAGTGCGTGGTCGGCGTCTCTCAAGGCACACAGGGGAAAGG

>HYQZ1MD01C85UW

CACAGTGCGTGGTCGGCGTCTCTCAAGGCACACAGGGG

>HYQZ1MD01A0AIW

ACAGTGCGTGGTCGGCGTCTCTCAAGGCACACAGGGGAAAGG

>HYQZ1MD01B3GL7

ACAGTGCGTGGTCGGCGTCTCTCAAGGCACACAGGGGAAAG

>HYQZ1MD01BAW5Q

CACAGTGCGTGGTCGGCGTCTCTCAAGGCACACAGGGG

>HYQZ1MD01DFPSF

CACAGTGCGTGGTCGGCGTCTCTCAAGGCACACAGGGGAAAGG

>HYQZ1MD01DD0SU

CACAGTGCGTGGTCGGCGTCTCTCAAGGCACACAGGGGAAAGG

>HYQZ1MD01BPR6N

CACAGTGCGTGGTCGGCGTCTCTCAAGGCACACAGGGGAAAGG

>HYQZ1MD01BYLU0

CACAGTGCGTGGTCGGCGTCTCTCAAGGCACACAGGGGAAAGG

>HYQZ1MD01C25KI

CACAGTGCGTGGTCGGCGTCTCTCAAGGCACACAGGGG

>HYQZ1MD01BSHD9

CACAGTGCGTGGTCGGCGTCTCTCAAGGCACACAGGGG

>HYQZ1MD01A6KSA

CACAGTGCGTGGTCGGCGTCTCTCAAGGCACACAGGGG

>HYQZ1MD01DQFQL

CACAGTGCGTGGTCGGCGTCTCTCAAGGCACACAGGGGAAAG

>HYQZ1MD01DNHEW

CACAGTGCGTGGTCGGCGTCTCTCAAGGCACACAGGGGAAA

>HYQZ1MD01CEBR9

CACAGTGCGTGGTCGGCGTCTCTCAAGGCACACAGGGGAAAGG

>HYQZ1MD01A9IEW

CACAGTGCGTGGTCGGCGTCTCTCAAGGCACACAGGGGAAAGG

>HYQZ1MD01BOB8I

CACAGTGCGTGGTCGGCGTCTCTCAAGGCACACAGGGGAAAGG

>HYQZ1MD01B79M7

CACAGTGCGTGGTCGGCGTCTCTCAAGGCACACAGGGGAAAGG

>HYQZ1MD01DJXPV

CACAGTGCGTGGTCGCGTCTCTCAAGGCACACAGGGGAAAG

>HYQZ1MD01DKAKN

CACAGTGCGTGGTCGGCGTCTCTCAAGGCACACAGGGGAAA

>HYQZ1MD01BMFZV

CACAGTGCGTGGTCGGCGTCTCTCAAGGCACACAGGGGAAA

>HYQZ1MD01DFAID

CACAGTGCGTGGTCGGCGTCTCTCAAGGCACACAGGGGAAAGG

>HYQZ1MD01BQQRD

CACAGTGCGTGGTCGGCGTCTCTCAAGGCACACAGGGGTAAACG

>HYQZ1MD01BLRGB

CACAGTGCGTGGTCGGCGTCTCTCAAGGCACACAGGGG

>HYQZ1MD01A5YCZ

CACAGTGCGTGGTCGGCGTCTCTCAAGGCACACAGGGGAAAGG

>HYQZ1MD01DDN1L

CACAGTGCGTGGTCGGCGTCTCTCAAGGCACACAGGGG

>HYQZ1MD01BS40L

CACAGTGCGTGGTCGGCGTCTCTCAAGGCACACAGGGG

>HYQZ1MD01AZRAX

CACAGTGCGTGGTCGGCGTCTCTCAAGGCACACAGGGGGAAAAGG

>HYQZ1MD01C3XQY

CACAGTGCGTGGTCGGCGTCTCTCAAGGCACACAGGGG

>HYQZ1MD01AOEGP

CACAGTGCGTGGTCGGCGTCTCTCAAGGCACACAGGGG

>HYQZ1MD01BKEJ3

CACAGTGCGTGGTCGGCGTCTCTCAAGGCACACAGGGGAAAGG

>HYQZ1MD01A6A78

ACAGTGCGTGGTCGGCGTCTCTCAAGGCACACAGGGG

>HYQZ1MD01BS8W0

CACAGTGCGTGGTCGCGTCTCTCAAGGCACACAGGGGAAA

>HYQZ1MD01BR9BW

CACAGTGCGTGGTCGGCGTCTCTCAAGGCACACAGGGG

>HYQZ1MD01AOXIR

CACAGTGCGTGGTCGGCGTCTCTCAAGGCACACAGGGG

>HYQZ1MD01B3LSU

ACAGTGCGTGGTCGGCGTCTCTCAAGGCACACAGGGGAAAGG

>HYQZ1MD01DFYA5

CACAGTGCGTGGTCGGCGTCTCTCAAGGCACACAGGGGAAAGG

>HYQZ1MD01B15Z3

CACAGTGCGTGGTCGCGTCTCTCAAGGCACACAGGGG

>HYQZ1MD01A0PVV

CACAGTGCGTGGTCGGCGTCTCTCAAGGCACACAGGGGAAAGG

>HYQZ1MD01C88XQ

CACAGTGCGTGTCGCGTCTCTCAAGGCACACAGGGG

>HYQZ1MD01BLVGU

CACAGTGCGTGGTCGGCGTCTCTCAAGGCACACAGGGGAAAGG

>HYQZ1MD01DCI7Q

CACAGTGCGTGGTCGGCGTCTCTCAAGGCACACAGGGGAAAGG

>HYQZ1MD01DBNRG

ACAGTGCGTGGTCGGCGTCTCTCAAGGCACACAGGGGAAAGG

>HYQZ1MD01BH0LX

ACAGTGCGTGGTCGGCGTCTCTCAAGGCACACAGGGGAAAGG

>HYQZ1MD01A84M6

CACAGTGCGTGGTCGGCGTCTCTCAAGGCACACAGGGG

>HYQZ1MD01BP10D

CACAGTGCGTGGTCGGCGTCTCTCAAGGCACACAGGGG

>HYQZ1MD01DGDQQ

CACAGTGCGTGGTCGGCGTCTCTCAAGGCACACAGGGGAAAGG

>HYQZ1MD01B2A91

CACAGTGCGTGGTCGGCGTCTCTCAAGGCACACAGGGGAAAGG

>HYQZ1MD01CYCYR

CACAGTGCGTGGTCGGCGTCTCTCAAGGCACACAGGGGAAAGG

>HYQZ1MD01BC8LW

CACAGTGCGTGGTCGGCGTCTCTCAAGGCACACAGGGGG

>HYQZ1MD01A5W7L

CACAGTGCGTGGTCGGCGTCTCTCAAGGCACACAGGGG

>HYQZ1MD01DA71O

CACAGTGCGTGGTCGGCGTCTCTCAAGGCACACAGGGGGTAAA

>HYQZ1MD01CQQVE

CACAGTGCGTGGTCGGCGTCTCTCAAGGCACACAGGGG

>HYQZ1MD01B4F89

CACAGTGCGTGGTCGGCGTCTCTCAAGGCACACAGGGGAAAGG

>HYQZ1MD01DNLR4

CACAGTGCGTGGTCGGCGTCTCTCAAGGCACACAGGGG

>HYQZ1MD01BUB6K

CACAGTGCGTGGTCGGCGTCTCTCAAGGCACACAGGGGAAAGG

>HYQZ1MD01AVOLU

CACAGTGCGTGGTCGGCGTCTCTCAAGGCACACAGGGGG

>HYQZ1MD01AB54O

CACAGTGCGTGTCGCGTCTCTCAAGGCACACAGGGG

>HYQZ1MD01CCY3G

CACAGTGCGTGGTCGGCGTCTCTCAAGGCACACAGGGG

>HYQZ1MD01B5SD7

ACAGTGCGTGGTCGGCGTCTCTCAAGGCACACAGGGGAAAGG

>HYQZ1MD01DQ96F

CACAGTGCGTGGTCGGCGTCTCTCAAGGCACACAGGGG

>HYQZ1MD01DO85W

ACAGTGCGTGGTCGGCGTCTCTCAAGGCACACAGGGGAAAGG

>HYQZ1MD01C1FZX

CACAGTGCGTGGTCGGCGTCTCTCAAGGCACACAGGGG

>HYQZ1MD01ASXOS

CACAGTGCGTGTCGCGTCTCTCAAGCACACAGGGG

>HYQZ1MD01C0WZQ

CACAGTGCGTGGTCGGCGTCTCTCAAGGCACACAGGGGAAAGG

>HYQZ1MD01DQH1U

CACAGTGCGTGGTCGGCGTCTCTCAAGGCACACAGGGGAAAGG

>HYQZ1MD01BW53I

CACAGTGCGTGGTCGGCGTCTCTCAAGGCACACAGGGGG

>HYQZ1MD01B6Q26

CACAGTGCGTGGTCGGCGTCTCTCAAGGCACACAGGGG

>HYQZ1MD01CAQ9L

CACAGTGCGTGGTCGGCGTCTCTCAAGGCACACAGGGGG

>HYQZ1MD01CZFT7

CACAGTGCGTGGTCGGCGTCTCTCAAGGCACACAGGGG

>HYQZ1MD01DA360

CACAGTGCGTGGTCGGCGTCTCTCAAGGCACACAGGGG

>HYQZ1MD01A16QU

CACAGTGCGTGGTCGGCGTCTCTCAAGGCACACAGGGG

>HYQZ1MD01DPQFT

CACAGTGCGTGGTCGGCGTCTCTCAAGGCACACAGGGG

>HYQZ1MD01BN3XE

CACAGTGCGTGTCGCGTCTCTCAAGGCACACAGGGG

>HYQZ1MD01B0JBA

CACAGTGCGTGGTCGGCGTCTCTCAAGGCACACAGGGGAAAGG

>HYQZ1MD01CXWPH

CACAGTGCGTGTCGCGTCTCTCAAGCACACAGGGAAAG

>HYQZ1MD01AVEBH

CACAGTGCGTGGTCGGCGTCTCTCAAGGCACACAGGGGAAAGG

>HYQZ1MD01DWM8E

CACAGTGCGTGGTCGGCGTCTCTCAAGGCACACAGGGG

>HYQZ1MD01CSHMD

CACAGTGCGTGGTCGGCGTCTCTCAAGGCACACAGGGGAAAGG

>HYQZ1MD01AF6AI

CACAGTGCGTGGTCGGCGTCTCTCAAGGCACACAGGGG

>HYQZ1MD01C09FJ

CACAGTGCGTGGTCGGCGTCTCTCAAGGCACACAGGGG

>HYQZ1MD01B2M2L

CACAGTGCGTGGTCGGCGTCTCTCAAGGCACACAGGGGAAAGG

>HYQZ1MD01CU330

ACAGTGCGTGGTCGGCGTCTCTCAAGGCACACAGGGGGAAAGG

>HYQZ1MD01BHCUD

CACAGTGCGTGGTCGGCGTCTCTCAAGGCACACAGGGG

>HYQZ1MD01C0IVA

CACAGTGCGTGGTCGCGTCTCTCAAGGCACACAGGGG

>HYQZ1MD01BM3Y6

ACAGTGCGTGGTCGGCGTCTCTCAAGGCACACAGGGGAAAGG

>HYQZ1MD01C1PMO

CACAGTGCGTGGTCGGCGTCTCTCAAGGCACACAGGGGAAAGG

>HYQZ1MD01BXTWO

CACAGTGCGTGGTCGGCGTCTCTCAAGGCACACAGGGGAAAGG

>HYQZ1MD01C6J4Z

CACAGTGCGTGGTCGGCGTCTCTCAAGGCACACAGGGG

>HYQZ1MD01DR0WC

CACAGTGCGTGGTCGGCGTCTCTCAAGGCACACAGGGGAAAGG

>HYQZ1MD01A20FN

CACAGTGCGTGGTCGGCGTCTCTCAAGGCACACAGGGGAAAGG

>HYQZ1MD01A3367

CACAGTGCGTGGTCGGCGTCTCTCAAGGCACACAGGGGG

>HYQZ1MD01CRLAM

CACAGTGCGTGGTCGGCGTCTCTCAAGGCACACAGGGG

>HYQZ1MD01BN5QF

CACAGTGCGTGGTCGGCGTCTCTCAAGGCACACAGGGGAAAGG

>HYQZ1MD01B68SR

CACAGTGCGTGGTCGGCGTCTCTCAAGGCACACAGGGGAAAGG

>HYQZ1MD01DOXQT

CACAGTGCGTGGTCGGCGTCTCTCAAGGCACACAGGGGAAAGG

>HYQZ1MD01CX43L

CACAGTGCGTGGTCGGCGTCTCTCAAGGCACACAGGGG

>HYQZ1MD01C6E93

CACAGTGCGTGGTCGGCGTCTCTCAAGGCACACAGGGG

>HYQZ1MD01DS2JG

CACAGTGCGTGGTCGGCGTCTCTCAAGGCACACAGGGG

>HYQZ1MD01BD2ML

CACAGTGCGTGGTCGGCGTCTCTCAAGGCACACAGGGGAAAGG

>HYQZ1MD01C5CU8

CACAGTGCGTGGTCGGCGTCTCTCAAGGCACACAGGGGAAAGG

>HYQZ1MD01BR1JG

CACAGTGCGTGGTCGGCGTCTCTCAAGGCACACAGGGGAAA

>HYQZ1MD01C0FWW

CACAGTGCGTGGTCGGCGTCTCTCAAGGCACACAGGGG

>HYQZ1MD01ACHCV

CACAGTGCGTGGTCGGCGTCTCTCAAGGCACACAGGGGAAA

>HYQZ1MD01CXGFK

CACAGTGCGTGGTCGGCGTCTCTCAAGGCACACAGGGGAAA

>HYQZ1MD01CIBOB

CACAGTGCGTGGTCGGCGTCTCTCAAGGCACACAGGGGTAAA

>HYQZ1MD01CVX0F

ACAGTGCGTGGTCGGCGTCTCTCAAGGCACACAGGGGAAAGG

>HYQZ1MD01AAP9N

CACAGTGCGTGGTCGGCGTCTCTCAAGGCACACAGGGG

>HYQZ1MD01CBPP6

CACAGTGCGTGGTCGGCGTCTCTCAAGGCACACAGGGGAAAGG

>HYQZ1MD01BG4ZC

CACAGTGCGTGGTCGGCGTCTCTCAAGGCACACAGGGG

>HYQZ1MD01AVXUO

CACAGTGCGTGGTCGGCGTCTCTCAAGGCACACAGGGG

>HYQZ1MD01CJWCE

CACAGTGCGTGGTCGGCGTCTCTCAAGGCACACAGGGGGTAAA

>HYQZ1MD01DC85K

CACAGTGCGTGGTCGGCGTCTCTCAAGGCACACAGGGGGAAAGG

>HYQZ1MD01A8QU4

CACAGTGCGTGGTCGGCGTCTCTCAAGGCACACAGGGG

>HYQZ1MD01A1XN6

ACAGTGCGTGGTCGGCGTCTCTCAAGGCACACAGGGGAAAG

>HYQZ1MD01DF3NU

CACAGTGCGTGGTCGGCGTCTCTCAAGGCACACAGGGGAAAGG

>HYQZ1MD01CDM1M

CACAGTGCGTGGTCGGCGTCTCTCAAGGCACACAGGGGAAAGG

>HYQZ1MD01DGZ9Q

CACAGTGCGTGGTCGGCGTCTCTCAAGGCACACAGGGG

>HYQZ1MD01C5QOM

CACAGTGCGTGGTCGGCGTCTCTCAAGGCACACAGGGGTAAA

>HYQZ1MD01CMA0H

ACAGTGCGTGGTCGGCGTCTCTCAAGGCACACAGGGGG

>HYQZ1MD01C3C9X

CACAGTGCGTGTCGCGTCTCTCAAGCACACAGGGGAAAGG

>HYQZ1MD01BKTIR

CACAGTGCGTGGTCGGCGTCTCTCAAGGCACACAGGGGAAAG

>HYQZ1MD01A3U4L

CACAGTGCGTGGTCGGCGTCTCTCAAGGCACACAGGGGG

>HYQZ1MD01DFX66

CACAGTGCGTGGTCGGCGTCTCTCAAGGCACACAGGGGAAAGG

>HYQZ1MD01C87I4

CACAGTGCGTGGTCGGCGTCTCTCAAGGCACACAGGGGAAA

>HYQZ1MD01CLOMQ

CACAGTGCGTGGTCGGCGTCTCTCAAGGCACACAGGGG

>HYQZ1MD01CF3T2

CACAGTGCGTGGTCGGCGTCTCTCAAGGCACACAGGGG

>HYQZ1MD01AOV5C

CACAGTGCGTGGTCGGCGTCTCTCAAGGCACACAGGGGGTAAAACGG

>HYQZ1MD01CSVJ8

CACAGTGCGTGGTCGGCGTCTCTCAAGGCACACAGGGGAAA

>HYQZ1MD01CX3FW

CACAGTGCGTGGTCGGCGTCTCTCAAGGCACACAGGGGG

>HYQZ1MD01AUDLY

CACAGTGCGTGGTCGGCGTCTCTCAAGGCACACAGGGG

>HYQZ1MD01C4AFT

CACAGTGCGTGGTCGGCGTCTCTCAAGGCACACAGGGG

>HYQZ1MD01BJA77

ACAGTGCGTGGTCGGCGTCTCTCAAGGCACACAGGGG

>HYQZ1MD01B99C0

CACAGTGCGTGGTCGGCGTCTCTCAAGGCACACAGGGGAAA

>HYQZ1MD01C1P87

CACAGTGCGTGGTCGGCGTCTCTCAAGGCACACAGGGG

>HYQZ1MD01A5IUF

CACAGTGCGTGGTCGGCGTCTCTCAAGGCACACAGGGGAAA

>HYQZ1MD01A8NGA

CACAGTGCGTGGTCGGCGTCTCTCAAGGCACACAGGGGGAAAGG

>HYQZ1MD01DU8JY

CACAGTGCGTGGTCGGCGTCTCTCAAGGCACACAGGGGG

>HYQZ1MD01BSWDK

CACAGTGCGTGGTCGGCGTCTCTCAAGGCACACAGGGGAAAGG

>HYQZ1MD01A66YI

CACAGTGCGTGGTCGGCGTCTCTCAAGGCACACAGGGG

>HYQZ1MD01CDANG

CACAGTGCGTGGTCGGCGTCTCTCAAGGCACACAGGGGAAAGG

>HYQZ1MD01A0QVZ

CACAGTGCGTGGTCGGCGTCTCTCAAGCACACAGGGGAAAG

>HYQZ1MD01AJ1KX

CACAGTGCGTGGTCGGCGTCTCTCAAGGCACACAGGGGAAAGG

>HYQZ1MD01BY9LW

CACAGTGCGTGGTCGGCGTCTCTCAAGGCACACAGGGG

>HYQZ1MD01C8YA3

ACAGTGCGTGGTCGGCGTCTCTCAAGGCACACAGGGGAAAGG

>HYQZ1MD01DTXN5

CACAGTGCGTGGTCGGCGTCTCTCAAGGCACACAGGGGGTAAAACGG

>HYQZ1MD01CFM9T

CACAGTGCGTGGTCGGCGTCTCTCAAGGCACACAGGGGAAAGG

>HYQZ1MD01CY86X

CACAGTGCGTGGTCGGCGTCTCTCAAGGCACACAGGGG

>HYQZ1MD01CCXGX

CACAGTGCGTGGTCGGCGTCTCTCAAGGCACACAGGGGAAAGG

>HYQZ1MD01ADMNV

CACAGTGCGTGGTCGGCGTCTCTCAAGGCACACAGGGGAAAGG

>HYQZ1MD01CFZEJ

CACAGTGCGTGGTCGGCGTCTCTCAAGGCACACAGGGGAAAGG

>HYQZ1MD01B3SM3

CACAGTGCGTGTCGCGTCTCTCAAGGCACACAGGGG

>HYQZ1MD01BLMJT

CACAGTGCGTGTCGGCGTCTCTCAAGGCACACAGGGGAAAGG

>HYQZ1MD01CVC5E

CACAGTGCGTGGTCGGCGTCTCTCAAGGCACACAGGGGAAAGG

>HYQZ1MD01DDFOA

CACAGTGCGTGGTCGGCGTCTCTCAAGGCACACAGGGGGAAAGG

>HYQZ1MD01AS6B0

CACAGTGCGTGGTCGGCGTCTCTCAAGGCACACAGGGGAAAGG

>HYQZ1MD01DGATO

CACAGTGCGTGGTCGGCGTCTCTCAAGGCACACAGGGG

>HYQZ1MD01CUAZY

CACAGTGCGTGGTCGGCGTCTCTCAAGGCACACAGGGGAAAGG

>HYQZ1MD01C3RZY

CACAGTGCGTGGTCGGCGTCTCTCAAGGCACACAGGGGAAAGG

>HYQZ1MD01DRY35

CACAGTGCGTGGTCGGCGTCTCTCAAGGCACACAGGGGAAA

>HYQZ1MD01ADAQX

CACAGTGCGTGGTCGGCGTCTCTCAAGGCACACAGGGGGTAAA

>HYQZ1MD01B1KU1

CACAGTGCGTGGTCGGCGTCTCTCAAGGCACACAGGGGAAAG

>HYQZ1MD01ARO2L

CACAGTGCGTGGTCGGCGTCTCTCAAGGCACACAGGGGAAAGG

>HYQZ1MD01DGJUT

CACAGTGCGTGTCGCGTCTCTCAAGGCACACAGGGG

>HYQZ1MD01AIXZA

CACAGTGCGTGGTCGGCGTCTCTCAAGGCACACAGGGG

>HYQZ1MD01C6QMB

CACAGTGCGTGGTCGGCGTCTCTCAAGGCACACAGGGGAAAGG

>HYQZ1MD01AXPUP

CACAGTGCGTGGTCGGCGTCTCTCAAGGCACACAGGGG

>HYQZ1MD01BZOKE

CACAGTGCGTGGTCGGCGTCTCTCAAGGCACACAGGGGAAAGG

>HYQZ1MD01AE4U1

CACAGTGCGTGGTCGGCGTCTCTCAAGGCACACAGGGG

>HYQZ1MD01B52L2

CACAGTGCGTGGTCGGCGTCTCTCAAGGCACACAGGGG

>HYQZ1MD01AFXV5

ACAGTGCGTGGTCGGCGTCTCTCAAGGCACACAGGGGAAAGG

>HYQZ1MD01AZ0MC

CACAGTGCGTGGTCGGCGTCTCTCAAGGCACACAGGGGAAA

>HYQZ1MD01DM2DV

CACAGTGCGTGGTCGGCGTCTCTCAAGGCACACAGGGGAAAGG

>HYQZ1MD01BXGOK

CACAGTGCGTGGTCGGCGTCTCTCAAGGCACACAGGGGAAAGG

>HYQZ1MD01CD74M

CACAGTGCGTGGTCGGCGTCTCTCAAGGCACACAGGGGAAAGG

>HYQZ1MD01C6CNG

CACAGTGCGTGGTCGGCGTCTCTCAAGGCACACAGGGGAAAGG

>HYQZ1MD01B84DZ

CACAGTGCGTGGTCGGCGTCTCTCAAGGCACACAGGGGG

>HYQZ1MD01BZRKH

CACAGTGCGTGGTCGGCGTCTCTCAAGGCACACAGGGGAAA

>HYQZ1MD01AGNQX

CACAGTGCGTGGTCGGCGTCTCTCAAGGCACACAGGGGTAAA

>HYQZ1MD01DCD5F

CACAGTGCGTGGTCGGCGTCTCTCAAGGCACACAGGGGAAAGG

>HYQZ1MD01AP99Z

CACAGTGCGTGGTCGGCGTCTCTCAAGGCACACAGGGGGAAAGG

>HYQZ1MD01AI4Z4

CACAGTGCGTGGTCGGCGTCTCTCAAGGCACACAGGGGTAAA

>HYQZ1MD01CICD9

CACAGTGCGTGGTCGGCGTCTCTCAAGGCACACAGGGGTAAA

>HYQZ1MD01BQR90

CACAGTGCGTGGTCGGCGTCTCTCAAGGCACACAGGGGAAA

>HYQZ1MD01CXPSR

CACAGTGCGTGGTCGGCGTCTCTCAAGGCACACAGGGG

>HYQZ1MD01C0E49

CACAGTGCGTGGTCGGCGTCTCTCAAGGCACACAGGGGAAAGG

>HYQZ1MD01CTPVN

CACAGTGCGTGGTCGGCGTCTCTCAAGGCACACAGGGG

>HYQZ1MD01ADDVM

CACAGTGCGTGGTCCGCGTCTCTCAAGGCACACAGGGGAAAGG

>HYQZ1MD01AKRI3

CACAGTGCGTGGTCGGCGTCTCTCAAGGCACACAGGGGG

>HYQZ1MD01CA2UU

CACAGTGCGTGGTCGGCGTCTCTCAAGGCACACAGGGGGAAAGG

>HYQZ1MD01AZS22

ACAGTGCGTGGTCGCGTCTCTCAAGCACACAGGGG

>HYQZ1MD01DC4XC

ACAGTGCGTGGTCGGCGTCTCTCAAGGCACACAGGGGAAAGG

>HYQZ1MD01A0OAB

CACAGTGCGTGGTCGGCGTCTCTCAAGGCACACAGGGG

>HYQZ1MD01C1QRF

CACAGTGCGTGGTCGGCGTCTCTCAAGGCACACAGGGG

>HYQZ1MD01ASS2K

ACAGTGCGTGGTCGGCGTCTCTCAAGGCACACAGGGG

>HYQZ1MD01B7BRA

CACAGTGCGTGGTCGGCGTCTCTCAAGGCACACAGGGGTAAA

>HYQZ1MD01BMQW3

CACAGTGCGTGGTCGGCGTCTCTCAAGGCACACAGGGGAAAGG

>HYQZ1MD01AL12W

CACAGTGCGTGGTCGGCGTCTCTCAAGGCACACAGGGGG

>HYQZ1MD01DNDOX

CACAGTGCGTGGTCGGCGTCTCTCAAGGCACACAGGGGAAAGG

>HYQZ1MD01BKETD

CACAGTGCGTGGTCGGCGTCTCTCAAGGCACACAGGGGAAA

>HYQZ1MD01AYYJO

CACAGTGCGTGGTCGGCGTCTCTCAAGCACACAGGGGAAAG

>HYQZ1MD01B9B29

CACAGTGCGTGGTCGGCGTCTCTCAAGGCACACAGGGGAAAGG

>HYQZ1MD01CO1LB

CACAGTGCGTGGTCGGCGTCTCTCAAGGCACACAGGGG

>HYQZ1MD01BJCIG

ACAGTGCGTGTCGCGTCTCTCAAGGCACACAGGGGAAAG

>HYQZ1MD01CA1K9

CACAGTGCGTGGTCGGCGTCTCTCAAGGCACACAGGGGTAAAGG

>HYQZ1MD01BZ4BX

CACAGTGCGTGGTCGGCGTCTCTCAAGGCACACAGGGG

>HYQZ1MD01BB6FR

CACAGTGCGTGGTCGCGTCTCTCAAGGCACACAGGGGAAAGG

>HYQZ1MD01DTJOU

ACAGTGCGTGGTCGGCGTCTCTCAAGGCACACAGGGGAAAGG

>HYQZ1MD01BO7IW

CACAGTGCGTGGTCGGCGTCTCTCAAGGCACACAGGGGAAA

>HYQZ1MD01DBYSC

CACAGTGCGTGGTCGGCGTCTCTCAAGGCACACAGGGG

>HYQZ1MD01CJM2I

CACAGTGCGTGGTCGGCGTCTCTCAAGGCACACAGGGGTAAA

>HYQZ1MD01B4SH7

CACAGTGCGTGGTCGGCGTCTCTCAAGGCACACAGGGG

>HYQZ1MD01CGITZ

CACAGTGCGTGGTCGGCGTCTCTCAAGGCACACAGGGG

>HYQZ1MD01CQNEK

CACAGTGCGTGGTCGGCGTCTCTCAAGGCACACAGGGG

>HYQZ1MD01BSQ0Q

CACAGTGCGTGGTCGGCGTCTCTCAAGGCACACAGGGG

>HYQZ1MD01BBCJT

CACAGTGCGTGGTCGGCGTCTCTCAAGGCACACAGGGGAAAGG

>HYQZ1MD01A2USG

CACAGTGCGTGGTCGGCGTCTCTCAAGGCACACAGGGGAAAGG

>HYQZ1MD01CS77U

CACAGTGCGTGGTCGGCGTCTCTCAAGGCACACAGGGGAAAGG

>HYQZ1MD01AN5EM

CACAGTGCGTGGTCGGCGTCTCTCAAGGCACACAGGGG

>HYQZ1MD01CMLJC

CACAGTGCGTGGTCGGCGTCTCTCAAGGCACACAGGGG

>HYQZ1MD01BAYU6

CACAGTGCGTGGTCGGCGTCTCTCAAGGCACACAGGGGGTAAAGG

>HYQZ1MD01DN7M2

CACAGTGCGTGGTCGGCGTCTCTCAAGGCACACAGGGGAAAGG

>HYQZ1MD01BELO6

CACAGTGCGTGGTCGGCGTCTCTCAAGGCACACAGGGGAAAGG

>HYQZ1MD01DWSU1

CACAGTGCGTGGTCGGCGTCTCTCAAGGCACACAGGGG

>HYQZ1MD01DMWC2

CACAGTGCGTGTCGCGTCTCTCAAGGCACACAGGGG

>HYQZ1MD01BV64D

CACAGTGCGTGGTCGGCGTCTCTCAAGGCACACAGGGG

>HYQZ1MD01A3JN7

CACAGTGCGTGGTCGGCGTCTCTCAAGGCACACAGGGGAAAGG

>HYQZ1MD01C35JW

CACAGTGCGTGGTCGGCGTCTCTCAAGGCACACAGGGG

>HYQZ1MD01B7QFI

CACAGTGCGTGGTCGGCGTCTCTCAAGGCACACAGGGGAAAGG

>HYQZ1MD01CD4JY

CACAGTGCGTGGTCGGCGTCTCTCAAGCACACAGGGG

>HYQZ1MD01BR2P8

CACAGTGCGTGGTCGGCGTCTCTCAAGGCACACAGGGGAAAGG

>HYQZ1MD01A8NRL

CACAGTGCGTGGTCGGCGTCTCTCAAGGCACACAGGGGAAA

>HYQZ1MD01C0IX4

CACAGTGCGTGGTCGGCGTCTCTCAAGGCACACAGGGG

>HYQZ1MD01C5YS3

CACAGTGCGTGGTCGGCGTCTCTCAAGGCACACAGGGG

>HYQZ1MD01BNWPQ

CACAGTGCGTGGTCGCGTCTCTCAAGGCACACAGGGGAAA

>HYQZ1MD01AGFJD

CACAGTGCGTGGTCGGCGTCTCTCAAGGCACACAGGGG

>HYQZ1MD01CLAHM

CACAGTGCGTGGTCGCGTCTCTCAAGGCACACAGGGGTAAA

>HYQZ1MD01A8UDG

CACAGTGCGTGGTCGGCGTCTCTCAAGGCACACAGGGGGAAA

>HYQZ1MD01CGR6T

CACAGTGCGTGGTCGGCGTCTCTCAAGGCACACAGGGG

>HYQZ1MD01C4XLQ

CACAGTGCGTGGTCGCGTCTCTCAAGGCACACAGGGGAAAG

>HYQZ1MD01A5W4C

CACAGTGCGTGGTCGGCGTCTCTCAAGGCACACAGGGGAAA

>HYQZ1MD01DK5QC

CACAGTGCGTGGTCGGCGTCTCTCAAGGCACACAGGGG

>HYQZ1MD01AM6I7

CACAGTGCGTGGTCCGCGTCTCTCAAGGCACACAGGGGTAAA

>HYQZ1MD01AKHPO

CACAGTGCGTGGTCGGCGTCTCTCAAGGCACACAGGGG

>HYQZ1MD01DNKAX

CACAGTGCGTGTCGGCGTCTCTCAAGGCACACAGGGG

>HYQZ1MD01AHQJ4

CACAGTGCGTGGTCGGCGTCTCTCAAGGCACACAGGGGGTAAA

>HYQZ1MD01C0TLM

CACAGTGCGTGGTCGGCGTCTCTCAAGGCACACAGGGG

>HYQZ1MD01DOFGE

CACAGTGCGTGGTCGGCGTCTCTCAAGGCACACAGGGGAAAGG

>HYQZ1MD01B61MB

CACAGTGCGTGGTCGGCGTCTCTCAAGGCACACAGGGG

>HYQZ1MD01DLR1U

CACAGTGCGTGGTCGGCGTCTCTCAAGGCACACAGGGGAAAGG

>HYQZ1MD01CZUGI

CACAGTGCGTGGTCGGCGTCTCTCAAGGCACACAGGGGG

>HYQZ1MD01CXUDR

CACAGTGCGTGGTCGGCGTCTCTCAAGGCACACAGGGGG

>HYQZ1MD01C7BJJ

CACAGTGCGTGGTCGGCGTCTCTCAAGGCACACAGGGGAAAGG

>HYQZ1MD01AP0BF

ACAGTGCGTGGTCGGCGTCTCTCAAGGCACACAGGGGAAAGG

>HYQZ1MD01CHMT5

CACAGTGCGTGGTCGGCGTCTCTCAAGGCACACAGGGG

>HYQZ1MD01ANHON

CACAGTGCGTGGTCGGCGTCTCTCAAGGCACACAGGGG

>HYQZ1MD01DMXJM

CACAGTGCGTGGTCGCGTCTCTCAAGGCACACAGGGG

>HYQZ1MD01DA1ZT

CACAGTGCGTGGTCGGCGTCTCTCAAGGCACACAGGGG

>HYQZ1MD01DN8LM

CACAGTGCGTGGTCGGCGTCTCTCAAGGCACACAGGGG

>HYQZ1MD01BCNJG

CACAGTGCGTGGTCGGCGTCTCTCAAGGCACACAGGGGAAAGG

>HYQZ1MD01A6HFP

CACAGTGCGTGGTCGGCGTCTCTCAAGGCACACAGGGG

>HYQZ1MD01CZAPH

CACAGTGCGTGGTCGGCGTCTCTCAAGGCACACAGGGG

>HYQZ1MD01COOG7

CACAGTGCGTGGTCGGCGTCTCTCAAGGCACACAGGGGAAAGG

>HYQZ1MD01BIMTS

ACAGTGCGTGGTCGGCGTCTCTCAAGGCACACAGGGGAAAGG

>HYQZ1MD01B911Y

CACAGTGCGTGGTCGGCGTCTCTCAAGGCACACAGGGG

>HYQZ1MD01BE4V6

CACAGTGCGTGGTCGGCGTCTCTCAAGGCACACAGGGGG

>HYQZ1MD01B7DSI

CACAGTGCGTGTCGGCGTCTCTCAAGCACACAGGGG

>HYQZ1MD01DIEQO

CACAGTGCGTGGTCGGCGTCTCTCAAGGCACACAGGGG

>HYQZ1MD01AWP5N

CACAGTGCGTGGTCGGCGTCTCTCAAGGCACACAGGGGG

>HYQZ1MD01ANL3O

CACAGTGCGTGGTCGGCGTCTCTCAAGGCACACAGGGGTAAAGG

>HYQZ1MD01DFMSL

CACAGTGCGTGGTCGGCGTCTCTCAAGGCACACAGGGGAAAGG

>HYQZ1MD01B01M1

CACAGTGCGTGGTCGGCGTCTCTCAAGGCACACAGGGG

>HYQZ1MD01B2BK6

CACAGTGCGTGGTCGGCGTCTCTCAAGGCACACAGGGG

>HYQZ1MD01BP7QX

CACAGTGCGTGGTCGGCGTCTCTCAAGGCACACAGGGGAAAGG

>HYQZ1MD01BJVTG

CACAGTGCGTGGTCGGCGTCTCTCAAGGCACACAGGGGAAAGG

>HYQZ1MD01CJH98

CACAGTGCGTGGTCGGCGTCTCTCAAGGCACACAGGGGAAAAGG

>HYQZ1MD01DJ232

CACAGTGCGTGGTCGGCGTCTCTCAAGGCACACAGGGG

>HYQZ1MD01CNFBV

CACAGTGCGTGGTCGGCGTCTCTCAAGGCACACAGGGG

>HYQZ1MD01CLDWS

CACAGTGCGTGTCGGCGTCTCTCAAGGCACACAGGGG

>HYQZ1MD01AR4OA

CACAGTGCGTGGTCGGCGTCTCTCAAGCACACAGGGG

>HYQZ1MD01AYBTJ

CACAGTGCGTGGTCGGCGTCTCTCAAGGCACACAGGGGGTAAAGG

>HYQZ1MD01DPVRO

CACAGTGCGTGGTCGGCGTCTCTCAAGGCACACAGGGGAAAGG

>HYQZ1MD01BYS2D

CACAGTGCGTGGTCGGCGTCTCTCAAGGCACACAGGGGTAAACGG

>HYQZ1MD01BMH2K

CACAGTGCGTGGTCGGCGTCTCTCAAGGCACACAGGGGAAA

>HYQZ1MD01CUTLZ

CACAGTGCGTGGTCGGCGTCTCTCAAGGCACACAGGGGAAAGG

>HYQZ1MD01CV9HC

CACAGTGCGTGGTCGCGTCTCTCAAGGCACACAGGGGAAAGG

>HYQZ1MD01AEMY3

CACAGTGCGTGGTCGGCGTCTCTCAAGGCACACAGGGGAAA

>HYQZ1MD01C3R54

CACAGTGCGTGGTCGCGTCTCTCAAGGCACACAGGGGAAAGG

>HYQZ1MD01A9I51

CACAGTGCGTGGTCGGCGTCTCTCAAGGCACACAGGGGAAAGG

>HYQZ1MD01DF7PS

ACAGTGCGTGGTCGGCGTCTCTCAAGGCACACAGGGGTAAA

>HYQZ1MD01CBOV9

CACAGTGCGTGGTCGGCGTCTCTCAAGGCACACAGGGG

>HYQZ1MD01B1K7W

CACAGTGCGTGGTCGGCGTCTCTCAAGGCACACAGGGGAAAGG

>HYQZ1MD01CIKBA

CACAGTGCGTGGTCGGCGTCTCTCAAGGCACACAGGGGAAA

>HYQZ1MD01A473S

CACAGTGCGTGGTCGGCGTCTCTCAAGGCACACAGGGGAAA

>HYQZ1MD01C7T4Q

CACAGTGCGTGGTCGGCGTCTCTCAAGGCACACAGGGG

>HYQZ1MD01CZIQI

CACAGTGCGTGGTCGGCGTCTCTCAAGGCACACAGGGGG

>HYQZ1MD01B0NTS

ACAGTGCGTGGTCGGCGTCTCTCAAGGCACACAGGGG

>HYQZ1MD01APIT1

CACAGTGCGTGGTCGGCGTCTCTCAAGGCACACAGGGGG

>HYQZ1MD01BNASR

CACAGTGCGTGGTCGGCGTCTCTCAAGGCACACAGGGG

>HYQZ1MD01B8UDF

ACAGTGCGTGGTCGGCGTCTCTCAAGGCACACAGGGGG

>HYQZ1MD01DEVNP

CACAGTGCGTGGTCGGCGTCTCTCAAGGCACACAGGGGAAAGG

>HYQZ1MD01CNME6

CACAGTGCGTGGTCGGCGTCTCTCAAGGCACACAGGGG

>HYQZ1MD01AZCBU

CACAGTGCGTGGTCGGCGTCTCTCAAGGCACACAGGGG

>HYQZ1MD01CWPQH

CACAGTGCGTGTCGCGTCTCTCAAGGCACACAGGGG

>HYQZ1MD01BJA8W

CACAGTGCGTGGTCGGCGTCTCTCAAGGCACACAGGGGAAA

>HYQZ1MD01B5RCM

CACAGTGCGTGGTCGGCGTCTCTCAAGGCACACAGGGGAAA

>HYQZ1MD01DFH2C

CACAGTGCGTGGTCGGCGTCTCTCAAGGCACACAGGGG

>HYQZ1MD01CIPOA

CACAGTGCGTGGTCGGCGTCTCTCAAGGCACACAGGGGAAAGG

>HYQZ1MD01DA8IN

CACAGTGCGTGGTCGGCGTCTCTCAAGGCACACAGGGGG

>HYQZ1MD01CWJKM

CACAGTGCGTGGTCGGCGTCTCTCAAGGCACACAGGGGAAAGG

>HYQZ1MD01CUZYF

CACAGTGCGTGGTCGGCGTCTCTCAAGGCACACAGGGGAAAGG

>HYQZ1MD01CD12X

CACAGTGCGTGGTCGCGTCTCTCAAGGCACACAGGGGAAAGG

>HYQZ1MD01CLL5J

CACAGTGCGTGGTCGGCGTCTCTCAAGGCACACAGGGGAAA

>HYQZ1MD01AQ0XY

CACAGTGCGTGGTCGGCGTCTCTCAAGGCACACAGGGGAAAGG

>HYQZ1MD01BBGYM

CACAGTGCGTGGTCGGCGTCTCTCAAGGCACACAGGGG

>HYQZ1MD01CR10C

CACAGTGCGTGGTCGGCGTCTCTCAAGGCACACAGGGGTAAA

>HYQZ1MD01CVXZO

CACAGTGCGTGGTCGGCGTCTCTCAAGGCACACAGGGGGTAAA

>HYQZ1MD01A659D

CACAGTGCGTGGTCGGCGTCTCTCAAGGCACACAGGGGAAAGG

>HYQZ1MD01CR0RP

CACAGTGCGTGGTCGCGTCTCTCAAGGCACACAGGGGAAAG

>HYQZ1MD01B21WS

CACAGTGCGTGGTCGGCGTCTCTCAAGGCACACAGGGG

>HYQZ1MD01AUTCD

CACAGTGCGTGGTCGGCGTCTCTCAAGGCACACAGGGGGTAAAGG

>HYQZ1MD01BRZO4

CACAGTGCGTGGTCGGCGTCTCTCAAGGCACACAGGGGG

>HYQZ1MD01CW1N3

CACAGTGCGTGGTCGGCGTCTCTCAAGGCACACAGGGGAAAGG

>HYQZ1MD01A5R05

CACAGTGCGTGGTCGGCGTCTCTCAAGGCACACAGGGGAAAGG

>HYQZ1MD01A72CI

CACAGTGCGTGGTCGGCGTCTCTCAAGGCACACAGGGGTAAACGG

>HYQZ1MD01BIS6Z

CACAGTGCGTGGTCGGCGTCTCTCAAGGCACACAGGGGGAAAGG

>HYQZ1MD01B7RFO

CACAGTGCGTGGTCGGCGTCTCTCAAGGCACACAGGGG

>HYQZ1MD01AGBZK

CACAGTGCGTGGTCGGCGTCTCTCAAGCACACAGGG

>HYQZ1MD01CI4PG

CACAGTGCGTGTCGGCGTCTCTCAAGGCACACAGGGG

>HYQZ1MD01BF1KD

CACAGTGCGTGGTCGGCGTCTCTCAAGGCACACAGGGGTAAA

>HYQZ1MD01A4K70

CACAGTGCGTGGTCGGCGTCTCTCAAGGCACACAGGGGAAA

>HYQZ1MD01AR1FL

CACAGTGCGTGGTCGCGTCTCTCAAGGCACACAGGGG

>HYQZ1MD01A1OY8

CACAGTGCGTGGTCGGCGTCTCTCAAGGCACACAGGGGAAAGG

>HYQZ1MD01BHXJF

CACAGTGCGTGGTCGGCGTCTCTCAAGGCACACAGGGGAAAGG

>HYQZ1MD01DKL1G

CACAGTGCGTGGTCGGCGTCTCTCAAGGCACACAGGGGAAAGG

>HYQZ1MD01BYRPL

CACAGTGCGTGGTCGGCGTCTCTCAAGGCACACAGGGGTAAAGG

>HYQZ1MD01B51DU

ACAGTGCGTGGTCGGCGTCTCTCAAGGCACACAGGGGGTAAA

>HYQZ1MD01BEZFV

CACAGTGCGTGGTCGGCGTCTCTCAAGGCACACAGGGGG

>HYQZ1MD01A2LR9

CACAGTGCGTGGTCGGCGTCTCTCAAGGCACACAGGGGG

>HYQZ1MD01BWQPW

CACAGTGCGTGGTCGGCGTCTCTCAAGGCACACAGGGGAAAGG

>HYQZ1MD01DSQV2

CACAGTGCGTGGTCGGCGTCTCTCAAGGCACACAGGGG

>HYQZ1MD01AYJF9

ACAGTGCGTGGTCGGCGTCTCTCAAGGCACACAGGGGAAAGG

>HYQZ1MD01DOEMN

CACAGTGCGTGGTCGGCGTCTCTCAAGGCACACAGGGGGAAAGG

>HYQZ1MD01DNI0R

CACAGTGCGTGGTCGGCGTCTCTCAAGGCACACAGGGGAAAGG

>HYQZ1MD01C0ZGC

CACAGTGCGTGGTCGGCGTCTCTCAAGGCACACAGGGG

>HYQZ1MD01BYPM8

CACAGTGCGTGGTCGGCGTCTCTCAAGGCACACAGGGGAAA

>HYQZ1MD01DXGDJ

CACAGTGCGTGGTCGGCGTCTCTCAAGGCACACAGGGGAAA

>HYQZ1MD01AFW00

CACAGTGCGTGGTCGGCGTCTCTCAAGGCACACAGGGGAAAGG

>HYQZ1MD01BBI8X

CACAGTGCGTGGTCGGCGTCTCTCAAGGCACACAGGGGAAAG

>HYQZ1MD01CVDM4

CACAGTGCGTGGTCGGCGTCTCTCAAGGCACACAGGGGAAAGG

>HYQZ1MD01B7WFL

CACAGTGCGTGGTCGGCGTCTCTCAAGGCACACAGGGGG

>HYQZ1MD01DPEBZ

CACAGTGCGTGGTCGGCGTCTCTCAAGGCACACAGGGGAAAGG

>HYQZ1MD01C4J7S

CACAGTGCGTGGTCGGCGTCTCTCAAGGCACACAGGGGGAAAGG

>HYQZ1MD01ARUBE

CACAGTGCGTGGTCGGCGTCTCTCAAGGCACACAGGGGTAAA

>HYQZ1MD01CEHQX

CACAGTGCGTGGTCGGCGTCTCTCAAGGCACACAGGGGAAAGG

>HYQZ1MD01A27A3

CACAGTGCGTGGTCGGCGTCTCTCAAGGCACACAGGGG

>HYQZ1MD01AIT9J

CACAGTGCGTGGTCGGCGTCTCTCAAGGCACACAGGGGGTAAAGG

>HYQZ1MD01CE3ND

CACAGTGCGTGGTCGGCGTCTCTCAAGGCACACAGGGGTAAA

>HYQZ1MD01AWUOA

ACAGTGCGTGGTCGCGTCTCTCAAGCACACAGGGGAAA

>HYQZ1MD01CG62W

CACAGTGCGTGGTCGGCGTCTCTCAAGGCACACAGGGG

>HYQZ1MD01BJ3L9

CACAGTGCGTGGTCGGCGTCTCTCAAGGCACACAGGGGTAAAA

>HYQZ1MD01CKIN4

CACAGTGCGTGGTCGGCGTCTCTCAAGGCACACAGGGG

>HYQZ1MD01DCFQD

CACAGTGCGTGGTCGGCGTCTCTCAAGGCACACAGGGGAAAGG

>HYQZ1MD01BI1W1

CACAGTGCGTGGTCGGCGTCTCTCAAGGCACACAGGGGTAAA

>HYQZ1MD01AJWM5

CACAGTGCGTGGTCGGCGTCTCTCAAGGCACACAGGGG

>HYQZ1MD01BFZQ8

ACAGTGCGTGGTCGGCGTCTCTCAAGCACACAGGGG

>HYQZ1MD01CQDC4

CACAGTGCGTGGTCGGCGTCTCTCAAGGCACACAGGGGG

>HYQZ1MD01DKAX5

CACAGTGCGTGGTCGGCGTCTCTCAAGGCACACAGGGG

>HYQZ1MD01DSQUO

CACAGTGCGTGGTCGGCGTCTCTCAAGGCACACAGGGGGAAA

>HYQZ1MD01CKRBI

CACAGTGCGTGGTCGGCGTCTCTCAAGGCACACAGGGGG

>HYQZ1MD01CWP3M

CACAGTGCGTGGTCGCGTCTCTCAAGGCACACAGGGG

>HYQZ1MD01BM2ZY

CACAGTGCGTGGTCGGCGTCTCTCAAGGCACACAGGGGAAAGG

>HYQZ1MD01AOWMH

CACAGTGCGTGGTCGGCGTCTCTCAAGGCACACAGGGG

>HYQZ1MD01DG2L4

CACAGTGCGTGGTCGGCGTCTCTCAAGGCACACAGGGGG

>HYQZ1MD01C9EVO

CACAGTGCGTGGTCGCGTCTCTCAAGCACACAGGGG

>HYQZ1MD01CPKND

CACAGTGCGTGTCGCGTCTCTCAAGCACACAGGGG

>HYQZ1MD01BPDWH

CACAGTGCGTGGTCGGCGTCTCTCAAGGCACACAGGGGAAAGG

>HYQZ1MD01C079V

CACAGTGCGTGGTCGGCGTCTCTCAAGGCACACAGGGGG

>HYQZ1MD01CM1VD

CACAGTGCGTGGTCGGCGTCTCTCAAGGCACACAGGGGGAAAA

>HYQZ1MD01BL5NH

CACAGTGCGTGGTCGGCGTCTCTCAAGGCACACAGGGGAAAGG

>HYQZ1MD01BAH51

CACAGTGCGTGGTCGGCGTCTCTCAAGGCACACAGGGG

>HYQZ1MD01B5C0M

CACAGTGCGTGGTCGGCGTCTCTCAAGGCACACAGGGGAAAGG

>HYQZ1MD01B86Z9

CACAGTGCGTGGTCGGCGTCTCTCAAGGCACACAGGGG

>HYQZ1MD01AXX9R

CACAGTGCGTGGTCGGCGTCTCTCAAGGCACACAGGGG

>HYQZ1MD01DDCYM

CACAGTGCGTGGTCGGCGTCTCTCAAGGCACACAGGGGAAAGG

>HYQZ1MD01BBZP6

CACAGTGCGTGGTCGGCGTCTCTCAAGGCACACAGGGG

>HYQZ1MD01DRXGB

CACAGTGCGTGGTCGGCGTCTCTCAAGGCACACAGGGGGTAAAA

>HYQZ1MD01BZD2R

CACAGTGCGTGTCGGCGTCTCTCAAGGCACACAGGGG

>HYQZ1MD01B0IRQ

CACAGTGCGTGGTCGGCGTCTCTCAAGGCACACAGGGGAAAGG

>HYQZ1MD01AGD1W

CACAGTGCGTGGTCGGCGTCTCTCAAGGCACACAGGGGGTAAAA

>HYQZ1MD01DEZOB

CACAGTGCGTGGTCGGCGTCTCTCAAGGCACACAGGGG

>HYQZ1MD01DOO78

CACAGTGCGTGGTCGGCGTCTCTCAAGGCACACAGGGGAAAGG

>HYQZ1MD01BMVT6

CACAGTGCGTGGTCGGCGTCTCTCAAGGCACACAGGGG

>HYQZ1MD01DR8YC

CACAGTGCGTGGTCGGCGTCTCTCAAGGCACACAGGGGAAAGG

>HYQZ1MD01A3NAW

CACAGTGCGTGGTCGGCGTCTCTCAAGGCACACAGGGG

>HYQZ1MD01CJZZK

CACAGTGCGTGGTCGGCGTCTCTCAAGGCACACAGGGGTAAA

>HYQZ1MD01CMGUK

CACAGTGCGTGTCGGCGTCTCTCAAGGCACACAGGGG

>HYQZ1MD01DIGO0

CACAGTGCGTGGTCGGCGTCTCTCAAGGCACACAGGGGG

>HYQZ1MD01DGOZN

CACAGTGCGTGGTCGGCGTCTCTCAAGGCACACAGGGGGTAAAA

>HYQZ1MD01DROX8

CACAGTGCGTGGTCGGCGTCTCTCAAGGCACACAGGGGAAAAGG

>HYQZ1MD01A355C

ACAGTGCGTGGTCGGCGTCTCTCAAGGCACACAGGGG

>HYQZ1MD01DM9KI

CACAGTGCGTGGTCGGCGTCTCTCAAGGCACACAGGGGAAAGG

>HYQZ1MD01DRX9K

CACAGTGCGTGGTCGGCGTCTCTCAAGGCACACAGGGG
